# Role of the BAHD1 Chromatin-Repressive Complex in Placental Development and Regulation of Steroid Metabolism

**DOI:** 10.1371/journal.pgen.1005898

**Published:** 2016-03-03

**Authors:** Goran Lakisic, Alice Lebreton, Renaud Pourpre, Olivia Wendling, Emanuele Libertini, Elizabeth J. Radford, Morwenna Le Guillou, Marie-France Champy, Marie Wattenhofer-Donzé, Guillaume Soubigou, Slimane Ait-Si-Ali, Jean Feunteun, Tania Sorg, Jean-Yves Coppée, Anne C. Ferguson-Smith, Pascale Cossart, Hélène Bierne

**Affiliations:** 1 Micalis Institute, INRA, AgroParisTech, Université Paris-Saclay, Équipe Microbiologie Cellulaire et Epigénétique, Jouy-en-Josas, France; 2 Unité des Interactions Bactéries-Cellules, Institut Pasteur, Paris, France; 3 INSERM U604, Paris, France; 4 INRA USC2020, Paris, France; 5 Institut Clinique de la Souris-ICS-MCI, PHENOMIN, CNRS UMR7104, INSERM U964, Université de Strasbourg, Illkirch, France; 6 Plateforme Transcriptome et Epigénome, Département Génomes et Génétique, Institut Pasteur, Paris, France; 7 Department of Genetics, University of Cambridge, Cambridge, United Kingdom; 8 Cambridge University Hospitals, NHS Foundation Trust, Cambridge, United Kingdom; 9 CNRS UMR8200 Stabilité génétique et oncogenèse, Université Paris-Saclay, Villejuif, France; 10 CNRS UMR7216, Université Paris Diderot-Paris 7, Paris, France; NIEHS, UNITED STATES

## Abstract

BAHD1 is a vertebrate protein that promotes heterochromatin formation and gene repression in association with several epigenetic regulators. However, its physiological roles remain unknown. Here, we demonstrate that ablation of the *Bahd1* gene results in hypocholesterolemia, hypoglycemia and decreased body fat in mice. It also causes placental growth restriction with a drop of trophoblast glycogen cells, a reduction of fetal weight and a high neonatal mortality rate. By intersecting transcriptome data from murine *Bahd1* knockout (KO) placentas at stages E16.5 and E18.5 of gestation, *Bahd1*-KO embryonic fibroblasts, and human cells stably expressing *BAHD1*, we also show that changes in BAHD1 levels alter expression of steroid/lipid metabolism genes. Biochemical analysis of the BAHD1-associated multiprotein complex identifies MIER proteins as novel partners of BAHD1 and suggests that BAHD1-MIER interaction forms a hub for histone deacetylases and methyltransferases, chromatin readers and transcription factors. We further show that overexpression of BAHD1 leads to an increase of MIER1 enrichment on the inactive X chromosome (Xi). In addition, BAHD1 and MIER1/3 repress expression of the steroid hormone receptor genes *ESR1* and *PGR*, both playing important roles in placental development and energy metabolism. Moreover, modulation of *BAHD1* expression in HEK293 cells triggers epigenetic changes at the *ESR1* locus. Together, these results identify BAHD1 as a core component of a chromatin-repressive complex regulating placental morphogenesis and body fat storage and suggest that its dysfunction may contribute to several human diseases.

## Author Summary

The importance of epigenetics in regulation and dysfunction of metabolic pathways is increasingly recognized but the underlying mechanisms and molecular actors involved remain incompletely characterized. Here, we provide evidence that the heterochromatinization factor BAHD1 cooperates with MIER proteins to assemble chromatin-repressive complexes that control a network of metabolic genes involved in placental and fetal growth and in cholesterol homeostasis.

## Introduction

Chromatin-based transcriptional repression is mediated by macromolecular complexes containing proteins involved in chromatin writing, reading, erasing and remodeling activities. The combinatorial assembly of subunits with transcription factors affects cell-specific gene expression in response to developmental, physiological or environmental stimuli. Chromatin-repressive complexes control key pathways during embryonic development and adult life; as a consequence, deregulation or abnormalities in their components can lead to a wide range of pathological processes [1, 2]. The importance of chromatin-modifiers in development, cell differentiation and disease is well illustrated for three complexes containing the histone deacetylases HDAC1 and HDAC2: NuRD [3, 4], Sin3A [5] and CoREST [6, 7] (For reviews, see [2, 8–10]). By a proteomic approach, we found that the Bromo-Adjacent-Homology domain-containing 1 (BAHD1) protein co-purifies with HDAC1/2, together with heterochromatin proteins HP1 and KAP1 (or TRIM28) in human embryonic kidney (HEK) 293 cells, suggesting that BAHD1 is a core component of a novel HDAC1/2-associated complex [11]. BAHD1 also interacts with the Methyl-CpG-binding protein MBD1 and the H3K9 methyltransferases (KMT) SETDB1 [12] and SUV39H1 [13] and acts as a repressor, pointing to a role of BAHD1 in heterochromatin-mediated transcriptional repression [12]. In agreement with this, overexpression of BAHD1 in human cells induces large-scale chromatin condensation [12] and changes in the DNA methylation landscape [14].

BAHD1-associated heterochromatic domains lack acetyl histone H4 and partially overlap with HP1α, a marker of constitutive heterochromatin, and/or with H3 trimethylated at lysine 27 (H3K27me3), a marker of facultative heterochromatin [12]. Furthermore, when overexpressed in human female cells, BAHD1 is enriched at the inactive X chromosome (Xi), a paradigm of facultative heterochromatin [12]. A study in mouse embryonic stem cells (mESCs) recently reported that BAHD1, HDAC1 and HDAC2 are pulled-down by CDYL, a transcriptional co-repressor that may play a role in the maintenance of the Xi [15]. Taken together, these data suggest that BAHD1 is a component of HDAC1/2-associated complexes involved in a variety of epigenetic mechanisms.

A single gene encodes BAHD1 in vertebrates and no ortholog is found in invertebrates or plants, suggesting that BAHD1 has vertebrate-specific functions. However, the low expression of BAHD1 in cell lines has hampered its functional characterization and, so far, the BAHD1 regulatory gene network is poorly characterized. We identified the insulin-like growth factor II (*IGF2)* transcript and its antisense transcript (*IGF2AS)* as BAHD1 targets in HEK293 embryonic cells [12]. We also demonstrated that infection by a bacterial pathogen triggers BAHD1-mediated repression of Interferon-Stimulated Genes (*ISGs*) in epithelial cells [11]. However, apart from bacterial infection, signals that control the expression and/or activity of BAHD1 are unknown.

The aim of the present study was to determine the physiological functions of BAHD1. We show that disruption of the *Bahd1* gene in the mouse leads to a placental growth defect associated with low birth weight and neonatal death, hypocholesterolemia and decreased body fat in surviving adults. Proteomic studies of BAHD1-associated proteins identify MIER proteins as novel BAHD1 partners. Our extensive characterization of the transcriptome strongly suggests that BAHD1-MIER complexes repress genes involved in the control of steroid/lipid metabolism both in mouse and human cells.

## Results

### *Bahd1*-deficient mice display hypocholesterolemia, hypoglycemia and a lower body weight

**Fig 1:**
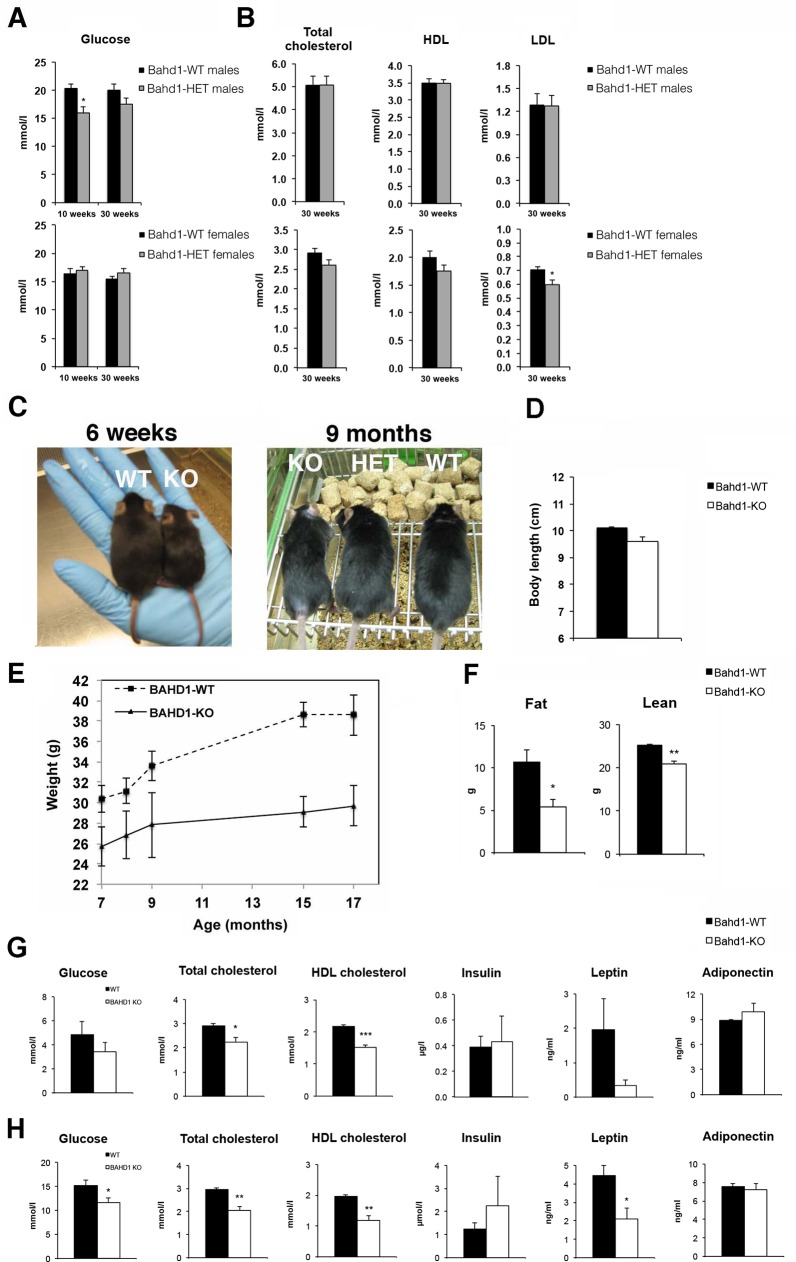
*Bahd1*-knockout mice display decreased weight and fat mass and lower cholesterol, glucose and leptin levels. **A-B.** Plasma levels of glucose (A) or of total cholesterol, HDL and LDL (B) in *Bahd1* wild-type (WT) and -heterozygous (HET) male (top) or female (bottom) mice, 10-week-old fed a chow diet (CD) or 30-week-old fed 14 weeks CD followed by 16 weeks HFHC (n = 8-10/group. **C.** Representative macroscopic images of *Bahd1*-WT and -knockout (KO) mice at 6 weeks and 9 months. **D.** Body length at 9 months. **E**. Body weight curve of *Bahd1-*WT and KO mice between the ages of 7 to 17 months (n = 5/group). **F.** Quantification of body fat mass and lean by QNMR analysis on 15 month-old animals (n = 4/group). **G.** Plasma levels of glucose, total cholesterol, HDL, insulin, leptin and adiponectin in 16-hours fasted 7-month-old WT and KO mice fed a normal diet (n = 4/group). **H.** Plasma levels of the same parameters in the same animals one year later (18 month-old). All data are expressed as the mean ± SE (* *P*<0.05; ** *P*<0.01; *** *P*< 0.005).

We searched for tissue-specific expression of the *BAHD1* gene by a survey of referenced transcriptome datasets (S1 Table) and found that *BAHD1* mRNA levels are low and do not vary much between tissues, when compared to a set of housekeeping or tissue-specific genes. This observation is consistent with the lack of detection of the endogenous BAHD1 protein in mammalian cultured cells and suggests that *BAHD1* basal levels are low. In order to identify functions of BAHD1 in biological processes, we studied the physiological consequences of *BAHD1* inactivation by performing a large-scale phenotyping of *Bahd1* haplo-deficient (*Bahd1*^+/-^) mice [11]. The analysis was carried out on 18 heterozygous (HET) and 16 wild type (WT) littermates (results are detailed in S1 Text and S2 Table). Mice were first fed a standard chow diet (CD) for 14 weeks and then a high fat/high carbohydrate diet (HFHCD) for 16 weeks. HET *Bahd1*^+/-^ mice did not show any morphological, sensory or cardiac abnormality and no change in bone density, body weight and fat, when compared to WT littermates, and the blood chemistry and hematology parameters were within the normal range. However, some slight and gender-specific modifications of glucose or cholesterol parameters were observed in HET mutants. At the age of 10 weeks, HET male mice displayed a significant hypoglycemia when compared to WT mice (Fig 1A). After the switch to HFHCD, blood levels of glucose remained lower in 30-week-old HET than in WT male mice, although difference did not then reach statistical significance (S2A Table). In contrast to males, glycemia was not affected in female HET mice whatever the diet (Fig 1A) but female HET fed with HFHCD displayed a slight decrease in blood levels of total cholesterol, high-density lipoprotein (HDL) and low-density lipoprotein (LDL) compared to WT littermates (Fig 1B). However, values were statistically significant only for LDL concentrations (S2B Table).

We next crossed *Bahd1*-heterozygous mice to evaluate potential metabolism defects in the *Bahd1-null* context. Most *Bahd1*^-/-^ (KO) pups died within the first days of life, indicating that the *Bahd1*-null mutation leads to perinatal death. Only 5 *Bahd1*^-/-^ out of 200 genotyped pups (2.5%*)* survived birth. At six weeks, these 5 survivors, all males, displayed reduced sizes and weights compared to control littermates (Fig 1C). After several months, they reached the length of *Bahd1*^+/+^ littermates (Fig 1C and 1D), but kept a significant lower body weight (Fig 1E), characterized by reduced fat and lean mass (Fig 1F). Quantification of blood parameters highlighted lower levels of total cholesterol, HDL and LDL in plasma of *Bahd1*^-/-^ mice than in *Bahd1*^+/+^ littermates at 3–5 months (S1A Fig), 7 and 18 months of age (Fig 1G and 1H). Glucose and leptin levels were also significantly lower in 18 month-old *Bahd1*^-/-^ mice than in *Bahd1*^+/+^ littermates (Fig 1H). The other measured blood parameters were not significantly different between KO and WT mice (i.e. insulin, adiponectin, Fig 1G and 1H; triglycerides, free fatty acids, glycerol, aspartate and alanine amino transferases, urea, creatinine, albumin, glucagon, GIP, S1B Fig). Together, these results demonstrate a biological function of BAHD1 in controlling lipid and carbohydrate metabolism.

### BAHD1 is required for mouse placental and fetal growth

**Fig 2:**
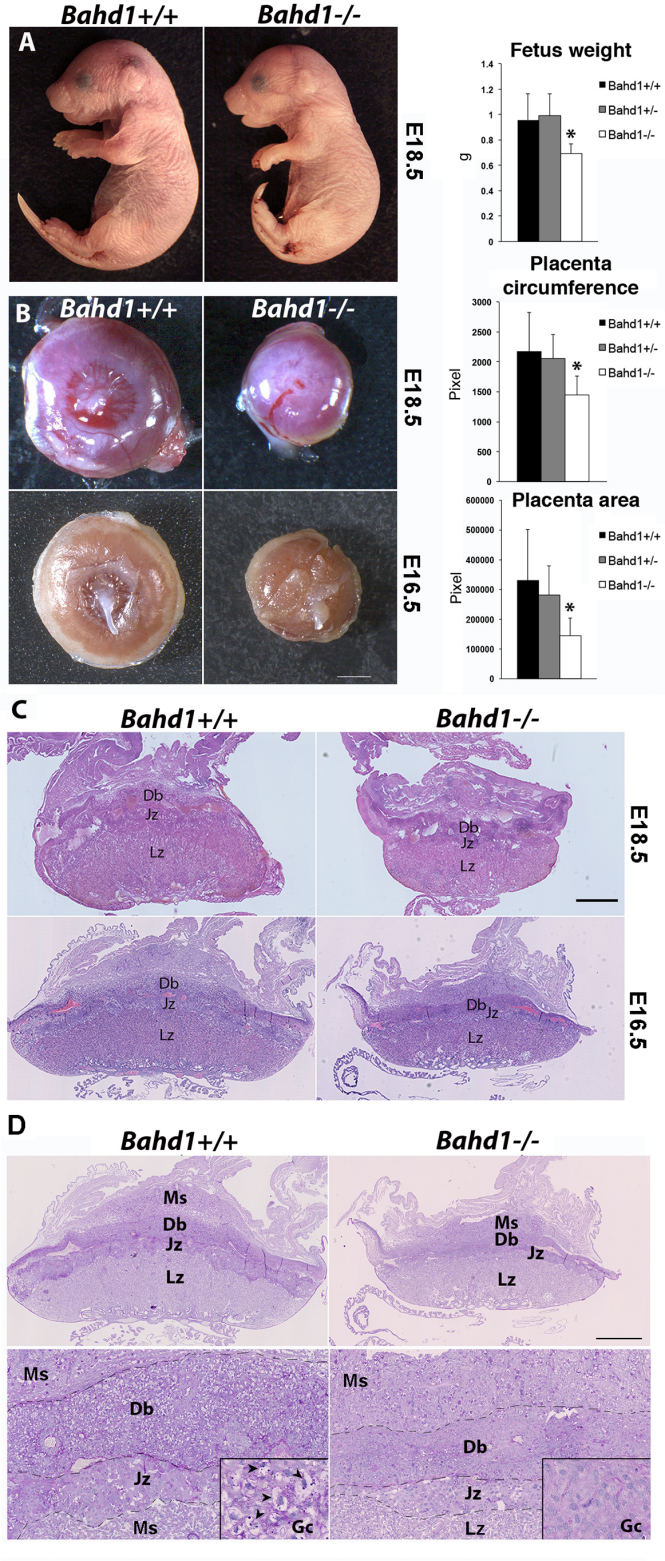
BAHD1 plays a role in fetal and placental growth. **A.** Representative images of *Bahd1*^+/+^ and *Bahd1*^−/−^ fetuses at E18.5 and quantification of fetus weight (on the right, * *P* < 0.05). **B.** Representative images of *Bahd1*^−/−^ and *Bahd1*^+/+^ placentas at E18.5 and E16.5. Scale bar, 200 mm. Quantification of areas and circumferences of E18.5 placentas are shown on the right (*Bahd1*^+/+^, n = 4; *Bahd1*^+/-^, n = 11; *Bahd1*^-/-^; n = 7). Data are expressed as mean ± SD (*P < 0.05). **C.** Histological analysis of *Bahd1*^−/−^ and *Bahd1*^+/+^ placentas at E18.5 and E16.5. Placentas were collected, fixed and subjected to hematoxylin and eosin (HE) staining. A representative image is shown. Scale bar: 1 mm. **D.** Periodic acid-Shiff (PAS) staining of the same E16.5 placentas as in C, at three different scales. Scale bar, 1 mm (top), 250μm (bottom), 60 μm (squared region). High magnification of vacuolated glycogen cells GCs (arrowheads) in the junctional zone is shown in squared regions. Ms, mesometrial triangle; Db: decidua basalis; Jz junctional zone; Lz, labyrinthine zone.

The high neonatal mortality rate of BAHD1-KO pups suggested that BAHD1 could have important functions during fetal life. In order to address this question, we examined embryos just before birth at the embryonic day 18.5 (E18.5). *Bahd1*^-/-^ fetuses were present at Mendelian frequency, had normal morphology, were alive and exhibited the breathing reflex. However, their weight was decreased by 30% (Fig 2A) when compared to WT or HET fetuses. In addition, *Bahd1*^-/-^ embryos exhibited a smaller placenta, with a reduction of 30% in the circumference and 55% of the area (Fig 2B), when compared to placentas of WT or HET embryos. At an earlier stage (E16.5), *Bahd1*^-/-^ placentas were also smaller than *Bahd1*^+/+^ ones (Fig 2B). Histology of placenta sections with hematoxylin and eosin (HE) staining showed that *Bahd1*^-/-^ placentas comprised the two fetal compartments (labyrinthine zone (Lz) and junctional zone (Jz)) and the maternal compartment (decidua basalis (Db)). However, the Lz surface area was significantly reduced and the Jz and Db were thinner in *Bahd1*^-/-^ placentas than in control littermates (Fig 2C). The junctional zone plays an important role in hormone synthesis, while the labyrinthine zone is critical for materno-foetal nutrient exchange [16, 17]. The Jz comprises fetal spongiotrophoblast cells and trophoblast glycogen cells (GCs). The exact origin and function of GCs is unknown, but they are believed to provide an important glucose supply for fetoplacental development. Evidence indicates that they differentiate early (E.6.5) in the ectoplacental cone at the origin of the Jz and migrate into the maternal decidua at about E12.5 [18, 19]. We used periodic acid-Shiff (PAS) staining to examine if GCs were altered by the *Bahd1* null mutation. PAS staining confirmed the reduced thickness of Jz and Db in *Bahd1*^-/-^ relative to *Bahd1*^+/+^ placentas at E16.5 and showed a severe reduction in the number of PAS-positive GCs both in the Jz and the Db (Fig 1D and S2A Fig). In contrast, histology of the fetal liver at E16.5 did not reveal any noticeable difference between *Bahd1*-KO and WT embryos (S2B Fig). Together, these results indicate that BAHD1 is required for a normal placental development and intra-uterine fetal growth. BAHD1 mutants may die after birth from metabolic defects, or secondary to altered placental exchange prenatally, impairing the development of energy stores.

### Expression of a set of steroid metabolism genes and imprinted genes is altered in *Bahd1*^-/-^ placentas

**Fig 3:**
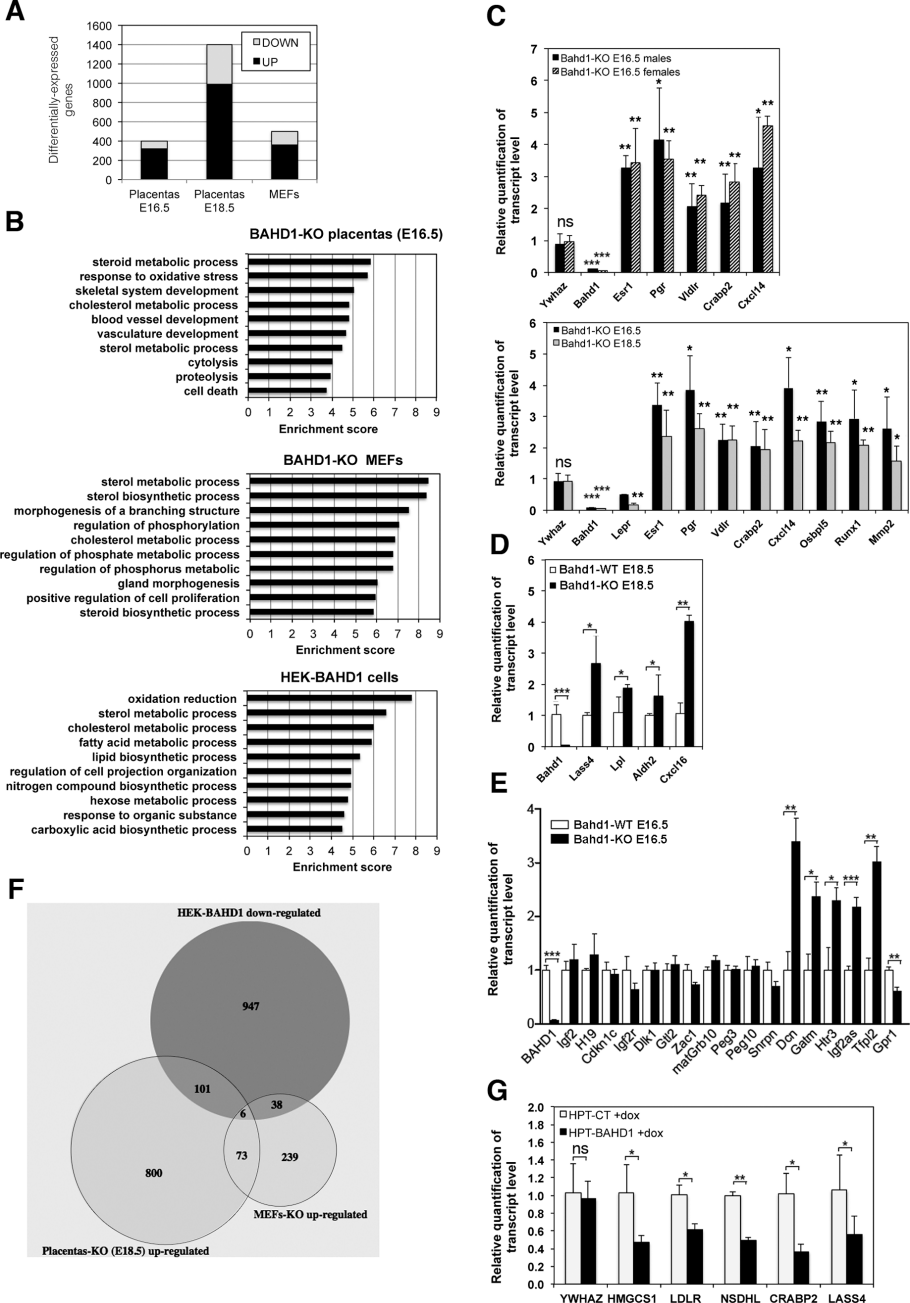
Loss or overexpression of BAHD1 alters expression of genes involved in steroid metabolism. **A.** Number of genes up- and down-regulated in *Bahd1*-KO placentas or MEFs relative to WT counterparts. **B.** Gene ontology enrichment analysis of transcripts differentially expressed in *Bahd1*-KO relative to WT placentas and MEFs, and in human HEK-BAHD1 cells relative to HEK-CT cells. The enrichment score represents the negative logarithm of the *p*-value evaluating the significance of gene ontology terms for differentially expressed RNAs. The top 10 annotation clusters are listed as derived from the DAVID bioinformatics tool. **C.** Transcripts levels in *Bahd1*^−/−^ placentas relative to *Bahd1*^+/+^ littermates at E16.5 (n = 6, with 3 males and 3 females for each genotype) or E18.5 (n = 3 for each genotype) were quantified by RT-qPCR. Values are normalized by *Gapdh*. *Ywhaz* is used as negative control. The differential expression in *Bahd1*^−/−^ placentas is shown (relative to that in *Bahd1*^+/+^ = 1). **D.** Relative transcripts levels for several genes in *Bahd1*^−/−^ relative to *Bahd1*^+/+^ placentas at E18.5 (n = 3 for each genotype). **E.** Relative transcripts levels for imprinted genes in *Bahd1*^−/−^ relative to *Bahd1*^+/+^ placentas at E16.5 (n = 4 for each genotype). Values are normalized to *Hprt*, *ActB and Tuba1a*. **F**. Venn diagram depicting shared genes that are up-regulated in *Bahd1*-KO E18.5 placentas and MEFs and down-regulated in human HEK-BAHD1 cells. **G**. Relative transcripts levels for lipid metabolism genes in HPT-BAHD1 cells relative to control HPT-CT. *BAHD1* expression was induced with tetracycline for 30h. Data are expressed as mean ± SD (ns, non-significant; * *P* < 0.05; ** *P* < 0.005; *** *P* < 0.001).

To examine the consequence of BAHD1 deficiency on the placental transcriptome, we isolated RNA from *Bahd1*^*-/-*^ placentas at stages E16.5 (n = 6 per genotype) and at stage E18.5 (n = 3 per genotype) to perform a comparative microarray analysis using Affymetrix mouse arrays. The *Bahd1*-null mutation altered expression of 397 and 1396 genes (FDR-BH < 0.05) at E16.5 and at E18.5, respectively, with a much higher proportion of up-regulated genes (70–80%) than down-regulated genes (20–30%), consistent with a role for BAHD1 in transcriptional repression (Fig 3A). 65% of genes deregulated in E16.5 *Bahd1*-KO placentas remained similarly deregulated at the E18.5 stage (214 up-regulated and 46 down-regulated genes, S3 Table). We used the DAVID software to classify genes based on Gene Ontology (GO) [20] and highlight the most significant biological processes that could be altered by BAHD1 deficiency. At the E16.5 stage, the most significant gene cluster (*P* value < 5.10^−4^) was a group of 16 genes involved in steroid metabolic processes (Fig 3B and S4A Table). Notably, 11 of these genes remained deregulated in *Bahd1*^*-/-*^ E18.5 placentas: 7 genes up-regulated (*Apoc3*, *Atp8b1*, *Cyp11A1*, *Insig2*, *Osbpl5*, *Pbx1*, *VldlR*) and 4 genes down-regulated (*Fabp6*, *Hsd17b2*, *Hsd17b7*, *LepR*) (S3 Table). In addition, two steroid hormone receptor genes, the estrogen receptor alpha gene *Esr1* and progesterone receptor gene *Pgr*, were up-regulated in *Bahd1*^*-/-*^ placentas, both in males and females embryos and at both gestational stages, as confirmed by Real time quantitative PCR (RT-qPCR) analysis (Fig 3C).

Groups of genes involved in the response to oxidative stress, skeletal system development and blood vessel development were also over-represented in *Bahd1*-KO placentas (S4 Table). Several of these genes are known to be involved in placental morphogenesis, such as *Gja1/Cx43*, *Cxcl14*, *Mmp2*, *Mmp14*, *Runx1* (up-regulated) and *Ada*, *Adm*, *Gcm1*, *Gjb5* (down-regulated). Altered expression of these genes in *Bahd1*-KO mice is consistent with an abnormal placentation. RT-qPCR analysis of a set of transcripts confirmed the transcriptome data (Fig 3C and 3D).

Several genes previously shown to be imprinted in the mouse during development or in the adult [21] were also up-regulated in *Bahd1*-KO placentas. Imprinted genes, expressed from only one of the parental alleles, play important functions during mammalian development, particularly in the placenta [22]. The parental conflict hypothesis of imprinting proposes that maternally− or paternally−expressed imprinted genes restrict or increase the energy expenditure, respectively [23]. We have previously shown that in human embryonic cells BAHD1 repressed *IGF2* and *IGF2AS* [12], which are imprinted in many tissues. BAHD1 deficiency did not affect *Igf2* expression in murine placenta, but, the paternally−expressed *Igf2as* and ten maternally−expressed putative imprinted (*Ampd3*, *Ano1*, *Dcn*, *Gatm*, *Htra3*, *Osbpl5*, *Qpct*, *Tfpi2*, *Tnfrsf23* and *Wt1*) were up-regulated in *Bahd1*-deficient E16.5 placentas, while one, *Gpr1*, was down-regulated. RT-qPCR assays confirmed these results using several other imprinted genes as negative controls (Fig 3E). Most of these genes are imprinted only in the placenta. However, determining placental specific imprinting can be confounded by maternally derived placental tissue and there is controversy over the imprinted status of some of these genes [24]. Strikingly, *Htra3*, *Tfpi2*, *Ampd3*, *Gatm*, *Osbpl5*, *Qpct* and *Wnt1* were also consistently up-regulated in *Bahd1*-deficient E18.5 placentas (S3 Table) and may be functionally implicated in the impaired placental growth.

### Steroid metabolism genes are also altered in *Bahd1*^-/-^ mouse MEFs and BAHD1-overexpressing human cells

*Bahd1*-KO fetuses exhibited a lower weight than *Bahd1*-WT. This phenotype could result from placental dysfunction but also from deregulation of BAHD1 target genes in intra-embryonic tissues. To address this point, we analyzed transcriptomes of mouse embryonic fibroblasts derived from *Bahd1*-WT and -KO embryos at stage E13.5 (n = 3 per genotype). The results of this analysis revealed 353 up-regulated and 139 down-regulated genes (FDR-BH < 0.05) in *Bahd1*-KO MEFs, when compared to *Bahd1*-WT MEFs (Fig 3A). We focused on up-regulated genes, as these likely contain those genes directly repressed by BAHD1. Strikingly, the most significant biological process associated with up-regulated genes in *Bahd1*-KO MEFs was sterol metabolism (Fig 3B). However, genes associated with this process were different to those affected in *Bahd1*-KO placentas (S4C Table), suggesting that BAHD1-dependent gene networks are tissue-specific even though they may have a common function.

We next assessed whether BAHD1 has a similar role in repressing sterol metabolism genes in human cells, by comparing transcriptomes of HEK293 cells stably overexpressing *BAHD1* (HEK-BAHD1) to that of parental isogenic cells (HEK-CT), in which the BAHD1 protein is undetectable [14]. This analysis identified 1148 transcripts (FDR-BH < 0.05) that were down-regulated in cells constitutively expressing the BAHD1 repressor. Once again, GO term classification analysis of these transcripts highlighted sterol metabolism as the most significant biological process (Fig 3B and S4D Table). Genes involved in lipid and hexose metabolism also grouped as significant clusters. Although biological functions of BAHD1-associated chromatin repressive complexes are likely to be cell type- and species-specific, and a transcriptome will identify direct and indirect gene expression changes, BAHD1 *bona fide* target genes may be identified by comparing different biological systems (Fig 3F). Overlapping HEK-BAHD1 and *Bahd1*-KO E18.5 placenta transcriptomes identified 107 potential BAHD1 targets (S5A Table and Fig 3F), the most significant gene groups where those involved in the regulation of hormone levels (*ALDH1A2*, *BACE2*, *CAMK2G*, *CRABP2*, *LY6E*, *SCARB1*, *SLC16A2*) and lipid biosynthetic processes (*ALDH1A2*, *CD81*, *EBP*, *ELOVL7*, *LASS4*, *LPL*, *LPCAT2*, *LTA4H*, *SCARB1*)(S4E Table). Overlapping HEK-BAHD1 and *Bahd1*-KO MEFs transcriptomes identified 44 potential BAHD1 target genes (S5B Table and Fig 3F), the most statistically significant group in term of biological processes including genes involved in sterol/steroid metabolism (*DHCR24*, *HMGCS1*, *LDLR*, *NSDHL*, *SC4MOL*, *SREBF2;* S4F Table). Only six genes were altered consistently between all the three transcriptome datasets (S5C Table). Using a tetracycline-inducible *BAHD1* HEK293 line (HPT-BAHD1 cells, [11]), we confirmed that induction of *BAHD1* expression (S3 Fig) is sufficient to repress five transcripts involved in lipid/steroid metabolism (*HMGCS1*, *LDLR*, *NSDHL*, *CRABP2* and *LASS4)* (Fig 3G). Of note, no imprinted gene was found in this analysis, indicating that the effect of the *Bahd1*-null mutation on this gene category is specific to the placenta. Altogether, these findings show that changes in BAHD1 levels alter expression of gene networks controlling steroid/lipid metabolism and hormone signaling pathways, which are key players in the development of the placenta and in control of energy in the body.

### BAHD1 co-purifies with MIER proteins, HDAC1/2 and several chromatin regulators

**Fig 4:**
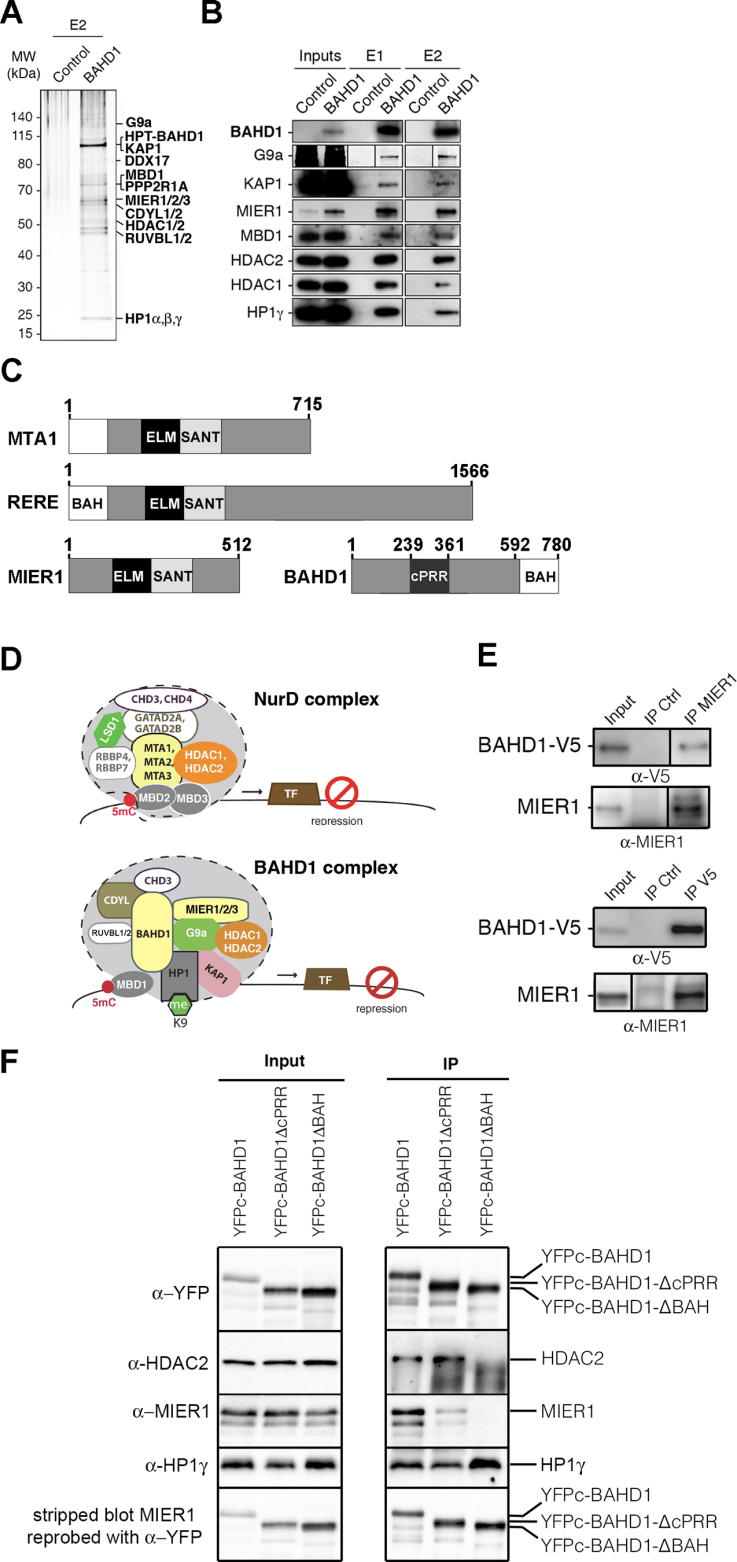
BAHD1 binds to MIER and HDACs. **A-B.** TAP-MS purification of the His_6_-Protein-C-BAHD1-associated complex. Solubilized chromatin extracts (“Inputs”) from HPT-CT cells (“Control”) or HPT-BAHD1 cells (“BAHD1”) were processed on anti–protein C affinity matrix (E1) and nickel-Sepharose (E2). Eluted fractions were separated by SDS-PAGE and analyzed by Mass Spectrometry. **A**. Silver staining of E2. **B**. Immunoblots of inputs E1 and E2, using antibodies against the specified proteins (for G9a, also see S4 Fig). **C.** Schematic representation of MTA1, RERE, MIER1 and BAHD1. ELM2, SANT, BAH domains, cPRR region and amino-acid numbers are indicated. **D.** Schematic diagrams of NurD and BAHD1 co-repressor complexes. Different protein paralogs can be part of distinct complexes. **E-F.** Nuclear extracts from HEK293-FT cells expressing BAHD1-V5 or YFPc-BAHD1, YFPc-BAHD1- ΔcPRR, YFPc-BAHD1- ΔBAH were used in immunoprecipitations (IP) assays with MIER1 or V5 antibodies or IgG control (**E**) or YFP antibodies. Vertical lines indicate cropping sites in original blots (see S5 Fig). (**F**). Eluted fractions were separated by SDS-PAGE and analyzed by immunoblot with the indicated antibodies (α-). In (F), the MIER1 blot was stripped and reprobed with YFP antibodies (see S5 Fig for a replicate experiment).

Efforts to perform chromatin immunoprecipitation (ChIP) of BAHD1 in murine or human cells using custom [12] or commercial BAHD1 antibodies repeatedly failed, preventing straightforward identification of genomic loci targeted by BAHD1 (BAHD1 ChIP is further discussed in S1 Text). To get further clues on BAHD1 function, we searched for novel BAHD1-associated proteins in cells stably expressing His_6_-Protein-C-tagged-BAHD1 (HPT-BAHD1) by using tandem affinity chromatography purification (TAP) and Mass spectrometry (MS), as described previously [11]. We found that ~10 proteins reproducibly co-purified with BAHD1 after two successive purification steps (Fig 4A and S6 Table): the mesoderm induction early response (MIER) proteins (MIER1, MIER2 and MIER3), HDAC1/2, HP1γ/β, KAP1, CDYL1, KAP1, PPP2R1A, RUVBL2 and DDX17. Other polypeptides were consistently detected in the first-step purification (HP1α, RUVBL1, CDYL2, DDX21, G9a, CHD3, S6 Table). Peptides matching with MBD1 were found in one TAP, in agreement with yeast two-hybrid, co-immunoprecipitation and GST pulldown assays that previously showed that BAHD1 interacts with MBD1 and HP1 [12]. Western blot analysis confirmed that BAHD1 co-purifies with MBD1 and HP1γ together with MIER1, HDAC1, HDAC2, KAP1 and the H3K9 KMT G9a (Fig 4B and S4 Fig). These data suggest that BAHD1 and MIER proteins are subunits of a macromolecular complex containing chromatin writers (e.g. G9a), readers (e.g. HP1α/β/γ, CDYL1/2, MBD1), erasers (e.g. HDAC1/2) and remodelers (e.g. CHD3, RUVBL1/2).

We noticed that BAHD1 and MIER1 displayed conserved domains found in scaffolding proteins of other HDAC1/2 complexes: the metastasis-associated protein (MTA) subunits of NuRD [8, 25] and RERE/atrophin-2, a transcriptional repressor belonging to a poorly characterized HDAC1/2 complex [26–28] (Fig 4C). BAHD1, MTA and RERE share a Bromo-Adjacent Homology (BAH) domain, which is known to promote protein-protein interactions and binding to nucleosomes [25, 29, 30], while MIER1, MTA and RERE proteins contain juxtaposed ELM2 and SANT domains that recruit HDACs [25]. Thus, BAHD1 and MIER1 could cooperate to fulfill a function similar to that of MTA and RERE in other HDAC1/2 complexes (Fig 4D). To investigate this hypothesis, we performed a series of co-immunoprecipitation experiments on HEK293-FT cells transiently expressing tagged-versions of BAHD1 from plasmid vectors, since endogenous BAHD1 is undetectable in HEK293 cells. Reciprocal co-IP assays with nuclear extracts confirmed association of endogenous MIER1 with V5-tagged BAHD1 in HEK293-FT cells and their co-immunoprecipitation with HDAC2 and HP1γ (Fig 4E and S5 Fig). Likewise, BAHD1 tagged with the fluorescent protein citrine (YFPc-BAHD1) pulled-down HDAC2, MIER1 and HP1γ (Fig 4F, lane 1).

BAHD1 harbors a N-terminal proline-rich region with the highest density of prolines found between residues 239 to 361, a region termed the cPRR [31], and a C-terminal BAH domain that interacts with the N-terminal tail of histone H3 [12] (Fig 4C). To assess the requirement of specific protein domains in BAHD1 for productive interaction with MIER and HDACs, we expressed truncated forms of YFPc-BAHD1 in HEK293-FT cells. As shown in Fig 4F and S5E Fig, the deletion mutant YFPc-BAHD1-ΔcPRR lacking residues 239 to 361 co-immunoprecipitated with HDAC2, MIER1 and HP1γ, as the full-length YFPc-BAHD1. In contrast, the deletion mutant YFPc-BAHD1-ΔBAH lacking residues 592 to 780 encompassing the BAH_624-780_ domain failed to pull-down HDAC2 and MIER1, while retaining the ability to bind HP1γ. We conclude that BAHD1 interaction with MIER1 and HDAC2 requires the integrity of the BAH domain of BAHD1.

**Fig 5:**
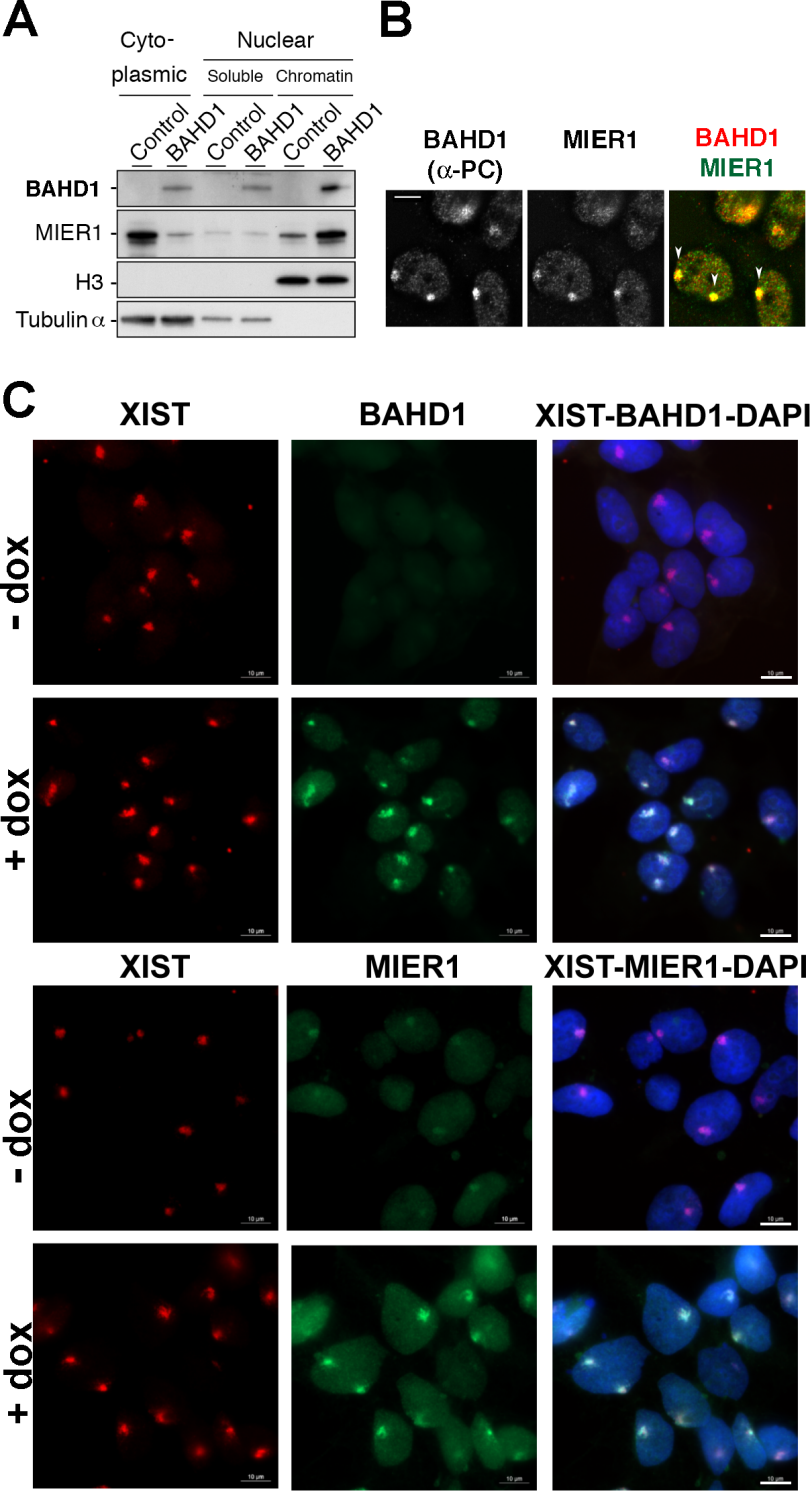
BAHD1 increases MIER1 nuclear translocation and recruitment to Xi. **A.** Cytoplasmic, nuclear soluble or chromatin extracts from HPT-control or HPT-BAHD1 cells induced 30 h with doxycyline were analyzed by immunoblotting with BAHD1, MIER1, histone H3 or Tubulin-α antibodies. H3 and Tubulin were used as loading controls. **B.** Colocalization of Protein-C tagged-BAHD1 and MIER1 on heterochromatic Xi (pointed with arrows on the merged image) was examined by IF with Protein-C and MIER1 antibodies. Bar, 5 μm. **C.** Localization of BAHD1 and MIER1 in HPT-BAHD1 cells induced or not for 30 h with doxycycline. Enrichment on Xi was determined by IF with BAHD1 (top panels) or MIER1 (bottom panels) antibodies combined with Xist RNA FISH (red). DNA was stained with DAPI (blue in the merged image). Bars: 10 μm.

We also found that varying BAHD1 amounts has an impact on MIER1 subcellular localization. First, MIER1 shifted from cytosolic to chromatin-bound fraction in response to induction of *BAHD1* expression in HPT-BAHD1 cells (Fig 5A). Second, microscopy experiments showed that BAHD1 overexpression increased MIER1 nuclear staining and induced enrichment of MIER1 at the heterochromatic inactive X chromosome (Xi) (Fig 5B and 5C). This effect was not due to changes in *MIER1* transcript levels (S3 Fig). The fact that BAHD1 levels dictate the localization of MIER1 strongly suggests that BAHD1 and MIER1 form a stable chromatin-bound complex. This hypothesis is also supported by results from the Heard laboratory [15] showing that BAHD1, MIER1/2, HDAC1/2 and G9a co-immunoprecipitate with CDYL, a protein recruited to Xi in mouse ES cells and also found in our TAP assays (S6 Table).

### Relationship between BAHD1 and metabolism-associated transcription factors

We propose that BAHD1 and MIER are co-repressors that, like MTA subunits of NurD complexes [9], exert their function through association with sequence-specific DNA binding transcription factors (TFs). With the hypothesis that such TFs should regulate the same gene networks as BAHD1, we used the Ingenuity Pathway Analysis Upstream Regulator software to predict TFs responsible for differential gene expression in BAHD1-deficient or -overexpressing cells. This analysis identified seven TFs that were consistently found as upstream regulators of a set of BAHD1-associated genes in all transcriptome datasets: ESR1, ESR2, EPAS1 (HIF2α), PPARG, FOS, TP53 and SP1 (S7 Table), the latter being previously reported to associate with BAHD1 [12] and MIER1 [32]. It is striking that these TFs have relevance both in placental development and regulation of genes involved in lipid/steroid metabolism ([33–36] and other references in S1 Text).

**Fig 6:**
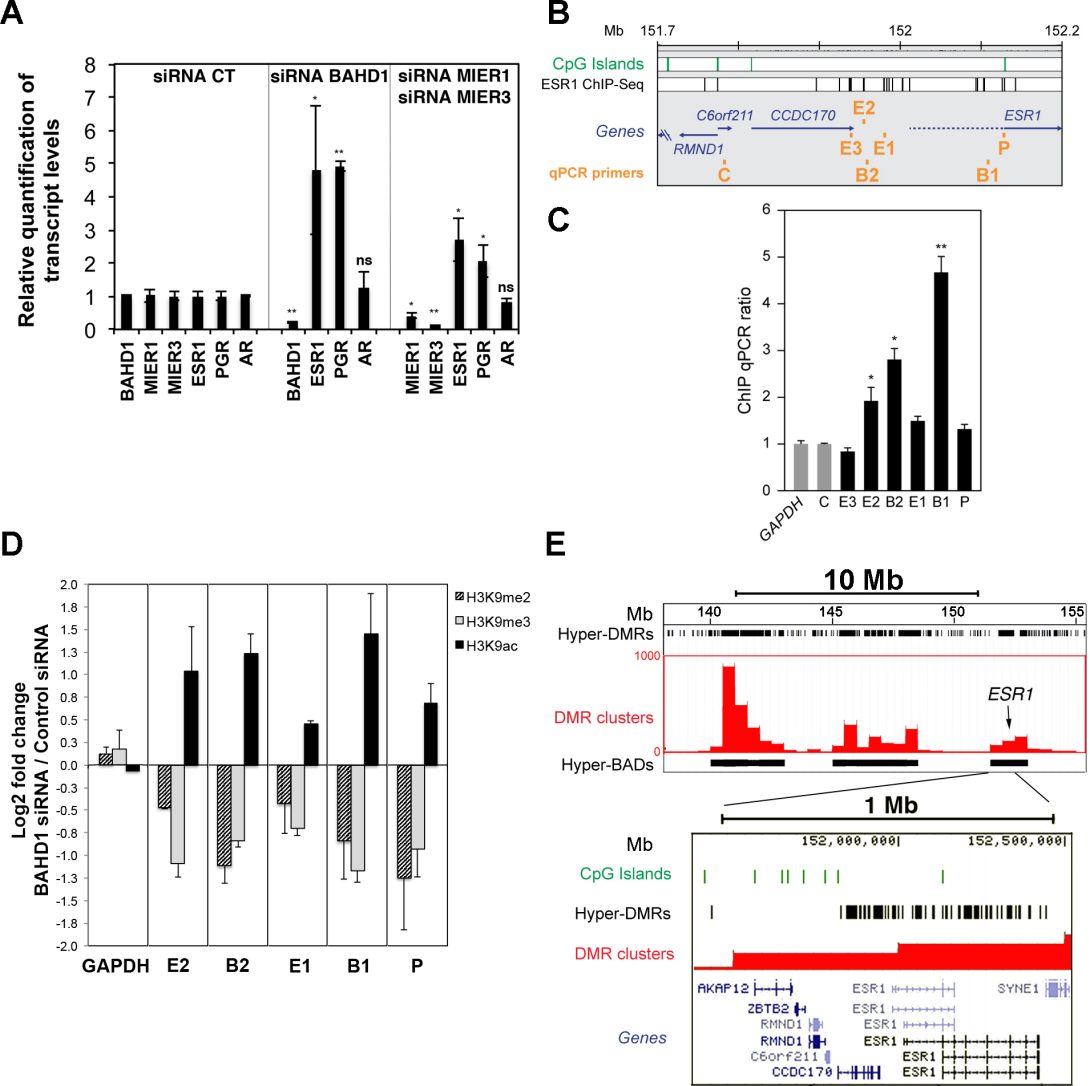
Depletion or overexpression of BAHD1 in HEK293 cells induce epigenetic changes at *ESR1*. **A. BAHD1 and MIER1/3 repress *ESR1* and *PGR***. HEK293-FT cells were transfected for 72 h with control or BAHD1, MIER1 or MIER3 siRNA. The levels of *BAHD1*, *MIER1*, *MIER3*, *ESR1*, *PGR* and *AR* transcripts were quantified by RT-qPCR. Data are expressed as mean ± SD (* *P*<0.05; ** *P*<0.005). **B.** Schematic representation of the proximal region of *ESR1* in chr6 of the human genome. **C.** BAHD1 binds the proximal region of *ESR1*. Amounts of DNA precipitated with BAHD1-TAP E1 eluates (see Fig 4) or with control TAP or inputs were quantified using qPCR with the primer sets indicated in B. The amount of DNA purified with BAHD1 was normalized to the amount precipitated in the control TAP, and to the *GAPDH* locus. Data are averages ± SD of qPCR triplicates, and representative of 2 independent TAP experiments. **D.** BAHD1 depletion alters the patterns of H3K9 acetylation and methylation at the *ESR1* locus. HEK293-FT cells were transfected with control or BAHD1 siRNA and enrichment of H3K9ac, H3K9me2 and H3K9me3 relative to IgG control at *ESR1* and *GAPDH* regions were estimated by ChIP-qPCR in BAHD1-depleted and control cells. The y-axis shows the relative fold change of ChIP enrichment in cells with BAHD1 siRNA over cells with control siRNA in Log2 ratios. Data are averages ± SD of two ChIP per antibody and representative of three biological replicates (see S6 Fig). **E.** Overexpression of BAHD1 induces widespread DNA methylation at the *ESR1* locus. Bisulfite-modified genomic DNA of control HEK-CT cells and isogenic HEK-BAHD1 cells overexpressing BAHD1 were sequenced and analyzed for their DNA methylation status, as described in Libertini et al. (2015) [14]. 300bp-regions with reproducible gain of methylation in HEK-BAHD1 compared to HEK-CT DNA in two BS-seq replicates (i.e hypermethylated BAHD1-DMRs) were binned into 0.5Mb windows highlighting clusters of hyper-DMRs (shown as red bars). Contiguous clusters define BAHD1-associated domains (“Hyper-BADs”, shown as black boxes). BAHD1-DMRs are represented by black vertical lines in the track “hyper-DMRs”. Three hypermethylated BADs of chr6 are shown on the top, with the position of *ESR1* indicated by an arrow. A 1 Mb region encompassing the *Esr1* locus is magnified below, showing the high density of hypermethylated BAHD1-DMRs on the whole locus. CpG islands are indicated in green. The position of transcripts is shown below.

Since ESR1 has been shown to bind MIER1 [37] and to cooperate with SP1 [38], we hypothesized that BAHD1-MIER could act as co-repressors for ESR1-mediated transcriptional regulation. Based on this assumption, BAHD1 should target the *ESR1* gene itself, because ESR1 autoregulates its own expression [39]. Accordingly, *ESR1* was up-regulated in *Bahd1*-KO placentas (Fig 3C), as well as in HEK293-FT cells depleted of BAHD1 with siRNA (Fig 6A). siRNA-mediated knockdown of individual MIER genes did not significantly change expression of *ESR1* in HEK293-FT cells, but combined knockdown of MIER1 and MIER3 (*MIER2* was undetectable) increased *ESR1* expression (Fig 6A). We also found that expression of *PGR* (encoding the progesterone receptor), which has an ESR1 binding site in its proximal region [40] significantly increased in BAHD1- and MIER1/3-knockdown cells (Fig 6A), whereas the androgen receptor transcript (*AR*) was unaffected. These results indicate that BAHD1 and MIER1/3 act as repressors for the steroid hormone receptor genes *ESR1* and *PGR*.

### *BAHD1* knockdown or overexpression induce epigenetic changes at the *ESR1* locus

Human *ESR1* forms a large and complex genetic unit that spans approximately 300 kb of chromosome 6 (chr6), of which 140 kb containing 8 protein-coding exons [41]. It is transcribed from at least seven promoters into multiple transcripts that vary in their 5’-UTRs and whose expression is tissue-specific. To find out whether the BAHD1-associated complexes targets *ESR1*, we analyzed the DNA captured with BAHD1 and its associated partners in the TAP assays by using real-time PCR with primers targeting the proximal region of *ESR1* at, or in the vicinity of ESR1-binding sites identified by ChIP-seq (Fig 6B, [40], ENCODE ChIP-seq data). Regions in *C6orf211* (Fig 6B) and *GAPDH* loci were used as controls. Three sites upstream of *ESR1* were identified as BAHD1 binding sites, when compared to input or control cell DNA (E2, B2, B1; Fig 6C). To determine whether any change in the chromatin structure occur as a consequence BAHD1 binding at these sites, we tested the effect of BAHD1 depletion on histone H3 modifications at lysine 9 (H3K9) by using a ChIP-qPCR assay. We observed a reproducible increase in histone H3K9 acetylation and decrease in H3K9 dimethylation (H3K9me2) and trimethylation (H3K9me3) in cells with siRNA−induced knockdown of *BAHD1* expression, relative to cells treated with control siRNA (Fig 6D and S6 Fig). This effect occurred at all sites of the *ESR1* proximal region surveyed by PCR, but not at the *GAPDH* control site. This result strongly suggests that a BAHD1-associated complex containing HDACs and KMTs contributes to the epigenetic silencing of *ESR1*.

H3K9 methylation is often linked to DNA methylation and several BAHD1-associated partners are known to interact with DNA methyltransferases [42–45]. To test whether BAHD1 level also affects DNA methylation patterns at *ESR1*, we exploited the DNA methylome datasets of HEK-CT and HEK-BAHD1 cells that we recently obtained by whole-genome bisulfite sequencing (BS-seq) [14]. In this study, we found that BAHD1 overexpression in HEK293 cells stimulates *de novo* DNA methylation in autosomes at ~80,000 regions that become hypermethylated when compared to control cells. These “BAHD1-DMRs” group into large (0.3–6.5 Mb) chromosomal domains, termed BAHD1-Associated Domains (BADs, [14]). We observed that one such BAD was present on chr6 in a large region containing the *ESR1* locus (Fig 6E). The high density of BAHD1-DMRs located at the *ESR1* proximal and gene body regions suggests that BAHD1 overexpression stimulates large-scale epigenetic changes. Taken together, these results provide evidence that BAHD1-associated complexes induce histone and DNA modifications that shape repressive chromatin structures.

## Discussion

### Developmental and physiological roles of BAHD1

The functions of BAHD1-mediated chromatin reorganization in developmental and physiological processes were hitherto unknown. Deletion of *Bahd1* in mice does not lead to embryonic lethality or anatomical malformations in fetuses. However, murine *Bahd1*-KO placentas are small and display histomorphological alterations, including reduced surface area of the labyrinthine zone and a thinner junctional zone and decidua. The lack of trophoblast glycogen cells is particularly striking, suggesting a failure in the differentiation program. The growth restriction of *Bahd1*-KO fetuses is likely to be secondary to defective placental nutrient exchange resulting from these placental abnormalities. In addition, as there is substantial deregulation of metabolic genes in *Bahd1-*KO MEFs, the perinatal death of *Bahd1-*KO pups likely results from metabolic defects in embryos. The rare mice surviving beyond birth are males with a lower weight than controls, associated with decreased fat mass and lower levels of circulating cholesterol, glucose and leptin. In light of our finding that the transcriptional co-repressor MIER1 is a key partner of BAHD1, it is striking that *Mier1* null mice also show decreased body weight and reduced levels of circulating glucose and cholesterol (data from The Mouse Phenotyping Consortium and The Wellcome Trust Sanger Institute, references in S1 Text). This phenocopy is a strong argument in favor of BAHD1 and MIER1 cooperating to control the expression of genes involved in energy metabolism in somatic tissues.

### BAHD1 and MIER proteins define a novel chromatin-repressive complex

Separate studies have shown that MIER1 binds to HDAC1/2, G9a and CDYL [27, 28, 46], while BAHD1 interacts with proteins involved in heterochromatin formation (e.g. HP1, MBD1) and its overexpression is sufficient to compact chromatin [12]. We now bring evidence that BAHD1 and MIER act in partnership within a novel histone deacetylase complex involved in gene silencing. First, BAHD1 co-purifies with MIER, HDAC1/2, G9a and CDYL in very different cellular models: human HEK293 cells, as shown here, and mouse ES cells [15]. Second, as in other subunits of HDAC1/2-associated complexes, MTAs of NuRD [8–10, 25](Fig 4D) and RERE [26–28] (Fig 4C), a BAH domain is present in BAHD1 and HDAC-interacting ELM2-SANT domains are present in MIER [27, 46]. We found that a truncation of the BAH domain in BAHD1 abrogates BAHD1 coimmunoprecipitation with MIER1 and HDAC2. From this, we propose that MTA, RERE and BAHD1-MIER define related macromolecular complexes of the “NuRD superfamily” and to name “BAHD1 complexes” those containing a BAHD1 subunit, as BAHD1 has no homolog or isoform. MIER1, MIER2 and MIER3 could be incorporated into distinct BAHD1 complexes leading to functional redundancy and/or context-dependent functions, as described for MTA1, MTA2 and MTA3. MIER1 is the most abundant partner of BAHD1 in HEK293 cells and its nuclear localization, including at the Xi, depends on BAHD1 expression levels. This translocation is likely to have functional consequences, as MIER1 nuclear/cytoplasmic distribution varies with cell type and stage of differentiation [47, 48]. *Bahd1-* and *Mier1*-KO adult mice share common phenotypes but a prenatal growth restriction is not reported for *Mier1*-KO; hence, the BAHD1-associated regulatory gene network in embryonic tissues likely involves other partners than MIER1.

### Regulation of metabolism gene networks by BAHD1

Genes altered in *Bahd1*-KO placentas or embryonic fibroblasts are mainly up-regulated compared to wild type controls, consistent with BAHD1 acting as a repressor. The most significant biological process associated with these genes, and with those repressed in BAHD1-overexpressing human HEK293 cells, is steroid metabolism. This functional convergence is striking and consistent with the observed hypocholesterolemia and hypolipidaemia in *Bahd*1-KO mice. We observed that genes associated with this biological function in *Bahd*1-KO MEFs and HEK-BAHD1 cells (e.g. *DHCR24*, *HMGCS1*, *LDLR*, *NSDHL*, *SREBF2*, *SC4MOL)* mostly differ with those in *Bahd1*-KO murine placentas (e.g. *Apoc3*, *Atp8b1*, *Cyp11A1*, *Insig2*, *Osbpl5*, *Pbx1*, *VldlR)*. Thus, like many other chromatin repressors [2], BAHD1 regulates distinct targets in different tissues and at different developmental stages. However, some of the BAHD1 *bona fide* target genes can be common to different biological systems, as exemplified by the steroid hormone receptor gene *ESR1*. BAHD1 binds the proximal region of human *ESR1* and represses *ESR1* both in human cells and murine placentas. In addition, changes in *BAHD1* expression levels in HEK293 cells alter the patterns of H3K9 modifications and DNA methylation at the *ESR1* locus, which is consistent with the interaction of BAHD1 with HDACs, KMTs, HP1 and MBD1 and the crosstalk between histone and DNA modifications [49].

It is worth mentioning that MTA1 [50] and SIN3a [39] have also been shown to represses *ESR1* in other cell types, indicating that different HDAC complexes control the epigenetic silencing of *ESR1*. Recruitment of BAHD1 at specific sites of the genome might rely on the combinatorial assembly of BAHD1-MIER subunits with transcription factors. The product of *ESR1* itself (ESR1/ERα) could be one such TF targeted by BAHD1 complexes because ESR1 autoregulates its own expression [39] and MIER1 has been shown to bind ESR1 [37]. In line with this hypothesis, ESR1 is predicted to control several genes differentially expressed in BAHD1-KO tissues, such as *PGR*. In addition, ESR1 cooperates with SP1 [38], a TF known to bind BAHD1 and MIER1 [12, 32], and which is also predicted to drive BAHD1-associated transcriptional changes. Our analysis also suggests that BAHD1 could act as a co-repressor with PPARγ and HIF2α, which like ESR1 are known to play roles in the regulation of energy metabolism and placental cell differentiation [33–36]. This raises the plausible hypothesis that the BAHD1-associated chromatin complex could act as a transcriptional co-repressor in synergy with different TFs in the context of placental functions and lipid metabolism.

### Role of BAHD1 in shaping the placental differentiation program

Placenta morphogenesis depends on the correct balance of cytotrophoblast proliferation and differentiation, into either syncytiotrophoblast involved in nutrient/gas exchange or invasive extravillous trophoblast involved in establishment of blood flow to the placenta. It is proposed that ESR1 controls the proliferation of estrogen-dependent cells, while ESR2 controls their maturation, hence trophoblast differentiation is associated with the transition from *Esr1* to *Esr2* expression [51, 52]. By maintaining unbalanced expression of *Esr1* at an inappropriate time of the gestation, BAHD1 deficiency could disturb trophoblast differentiation. *Pgr* and *Lepr* genes are also deregulated in *Bahd1*-KO placentas; this should affect progesterone and leptin signaling, also important in placental development [53, 54]. Therefore, BAHD1 could play a role in trophoblast differentiation, in particular in the formation of glycogen-producing cells, by controlling hormone signaling in a timely manner, particularly in the junctional zone, an important endocrine region. Knockdown of *Bahd1* in the mouse placenta results in up-regulation of several other genes that also have relevance to placental development. Among them, *Htra3* and *Tfpi2* are two confirmed imprinted genes expressed from the maternally inherited allele [24]. Maternally expressed genes have been proposed to limit maternal resource provision; thus up-regulation of such genes is consistent with a placental growth restriction observed upon BAHD1 deficiency. In fact, *HtrA3* and *Tfpi2* are highly transcribed in the placenta and involved in the regulation of endothelial function, trophoblast migration and invasion [55–58]. Further studies will be required to explore whether BAHD1 directly targets these genes and contributes to their imprinting.

### Roles of BAHD1 in diseases

With the importance of the placenta in the feto-maternal exchange processes, as well of steroid signaling in the body, alterations in the amounts or activity of BAHD1 may lead to various pathological processes. We previously identified a role for BAHD1 in infection of epithelial cells with the bacterial pathogen *Listeria monocytogenes* [11, 31]. The fact that BAHD1 is involved in placental function now opens the possibility that BAHD1 contributes to the fetoplacental step of listeriosis, as *L*. *monocytogenes* has a tropism for the placenta. More generally, a connection between BAHD1 and placenta-associated pathologies, such as Intrauterine Growth Restriction and Pre-Eclampsia, should be carefully examined. BAHD1-mediated regulation of *ESR1* is also enticing as ESR1 is a key regulator in a variety of biological processes and ESR1 deregulation has been implicated in several diseases, including breast cancer [59]. A shift from nuclear to cytoplasmic localization of MIER1 during breast cancer progression has been observed, suggesting that nuclear MIER1 contributes to the repression of genes involved in invasive breast carcinoma [37]. Similar to chromatin-repressive complexes Polycomb and NuRD [9, 60], BAHD1 could be implicated in the regulation of transcriptional events involved in diverse oncogenic pathways. Of note, insertion events in *BAHD1* and *MIER1* genes were identified in a screen for genes that cooperate with oncogenic KRAS (G12D) to accelerate tumorigenesis and promote progression in a mouse model of pancreatic ductal preneoplasia [61]. Our data also implicate BAHD1 in mammalian metabolic regulation and several BAHD1 candidate target genes have been associated with metabolic diseases in humans. For instance, genetic polymorphisms in *CRABP2* are associated with changes in plasma cholesterol levels [62], SNPs in *LPL* and *LASS4* are associated with dyslipidemia [63–65], changes in expression levels of *HMGCS1*, *LDLR* and *SC4MOL* correlated with obesity-related type 2 diabetes and cardiovascular diseases [66]. Together, these results indicate that dysfunction of BAHD1 complexes could promote aberrant epigenetic phenomena at the origin of different disorders. Detailed understanding of how and where BAHD1 complexes establish repressive chromatin states could be instrumental for the development of new strategies for selective treatment of metabolic disorders in the future.

## Methods

### Ethics statement

Mice were bred and maintained in the animal facilities of the Institut Clinique de la Souris (ICS, Illkirch, France) under pathogen-free conditions. The ICS facilities are licensed by the French Ministry of Agriculture (agreement #A67-218-37). All animal procedures were approved by the local ethical committee CREMEAS (registered under the reference “C2EA– 35”), and were supervised by M.F.C. and O.W. who are qualified in compliance with the European Community guidelines for laboratory animal care and use (2010/63/UE Directive).

### Cell lines, plasmids, antibodies, siRNAs and immunostaining

Human cell lines derive from HEK293 cells (ATCC CRL-1573): the HPT-BAHD1 inducible line and its isogenic HPT-control are described in [11]; the HEK-BAHD1 constitutive line and its isogenic HEK-CT control are described in [14]; HEK293-FT are from Invitrogen (ThermoFisher Scientific). Plasmid pcV5-BAHD1 (BUG2289), pYFPc (also named pEYFP-Citrine-N1, BUG2897), pYFPc-BAHD1 (BUG2897) and pYFPc-BAHD1-ΔBAH (BUG2740) are described in [12]. pYFPc-BAHD1ΔcPRR (BUG2897) is described in [31]. Antibodies were against BAHD1 (Abcam, 46573), MIER1 (Sigma, HPA019589), HDAC1 (Abcam, ab7028), HDAC2 (Abcam, ab7029), HP1γ (Euromedex, 2MOD-1G6-AS), KAP1 (Abcam, ab10483), G9a (MBL Cliniscience, D141-3), MBD1 (Abcam, ab3753), tubulin α (Santa Cruz, sc5546), H3K9me2 (Abcam ab1220), H3K9me3 (Abcam ab8898), H3K9ac (Upstate/Millipore 07–352), V5 and V5-HRP (Invitrogen R960-25, R961-25), GFP (Mouse anti-GFP Sigma/Roche 11814460001 used in IP and Rabbit anti-GFP Santa-Cruz sc-8334, used in WB, which both recognize YFPc) and control IgG mouse (Santa-Cruz sc-2025) and IgG rabbit (Santa-Cruz sc-2027). Fluorescent secondary antibodies were from Jackson ImmunoResearch or Molecular Probes, and HRP-conjugated secondary antibodies were from AbCys or Abcam (IgG Veriblot for IP, ab131368). siRNAs were purchased from Dharmacon (ThermoFisher Scientific) as follows: on-TARGETplus Non-targeting pool (D-001810-10-20), BAHD1 (L-020357-01), MIER1 (M-014201-02), MIER2 (M-023917-01), MIER3 (M-015618-01). Cells were transfected 72h with siRNA using Lipofectamine RNAimax (Life Technologies, Grand Island, NY) according to the manufacturer's instructions. Immunofluorescence and XIST FISH assays were as described in [12].

### Mice breeding and phenotyping

*Bahd1*^+/-^ mice have been described in [11]. Mice were bred and maintained in the animal facilities of the Institut Clinique de la Souris (ICS, Illkirch, France) under pathogen-free conditions. Throughout the experiment mice were housed in the same climate-controlled stable with a 12h/12h dark-light cycle and handled identically. For wild type and *Bahd1*^*-/-*^ production, *Bahd1*^+/-^ mice were mated and the day on which a vaginal plug was found was designated 0.5. Genotyping protocols are described in [11]. Knockout of *Bahd1* expression was verified by RT-qPCR on placenta, MEF and embryo liver samples. Phenotyping methods of *Bahd1* heterozygous and knockout mice and isolation of primary mouse embryonic fibroblasts are described in S1 Text. All the data are expressed as mean ± SE. Statistical analysis were performed using a one way ANOVA tests followed by a Fischer’s PLSD test with significance set at *P*<0.05. * *P*<0.05, ** *P*<0.01, *** *P*<0.0001.

### Microarray processing and analysis

Total RNAs were extracted from *Bahd1*^*+/+*^ *and Bahd1*^-/-^ placentas at E16.5 (n = 6/genotype) or E18.5 (n = 3/genotype), as well as from *Bahd1*^*+/+*^ *and Bahd1*^-/-^ MEFs (n = 3/genotype) and HEK-CT and HEK-BAHD1 cells (n = 3/cell line) using RNeasy Kit (Qiagen), treated TURBO DNA-freeTM kit (Ambion). RNA concentration and integrity were tested with RNA quality was monitored on Agilent RNA Pico LabChips (Agilent Technologies, Palo Alto, CA). 100 ng of RNA per sample were used as templates for the synthesis of hybridization probes for Affymetrix GeneChip Microarrays (Genechip HuGene 1.0 ST for HEK-CT and HEK-BAHD1 cells; Mouse gene 1.0 for E16.5 placentas; Mouse Exon 1.0 ST for E18.5 placentas and MEFs). Hybridization was carried out with biological replicates according to the expression analysis technical manual with wash and stain kit (Affymetrix). Gene-level expression values were derived from the CEL file probe-level hybridization intensities using the model-based Robust Multichip Average algorithm (RMA) [67]. RMA performs normalization, background correction and data summarization. An analysis is performed using the LPE test [68] and a *p*-value threshold of *p*<0.05 is used as the criterion for expression. The estimated false discovery rate (FDR) of this analyze was calculated using the Benjamini Hochberg approach in order to correct for multiple comparisons. Results were annotated using information provided by Affymetrix. Full data sets were reduced by discarding genes with “EST” and “unknown” annotation labels. To generate functional clusters of genes, we used the DAVID program (http://david.abcc.ncifcrf.gov, 2015 version; [20]) for selected gene sets according to gene ontology (GO) of biological process categories. To search for transcriptional regulators driving the differential gene expression changes we used the Ingenuity Pathway Analysis Upstream Regulator software [69]. Datasets have been deposited in Gene Expression Omnibus (GEO) and are accessible through accession number, as follows: GEO series GSE51868 (transcriptomes of HEK-CT and HEK-BAHD1); GSE53443 and GSE53442 (transcriptome of *Bahd1*-WT and *Bahd1*-KO placentas at E16.5 and at E18.5) and GSE73816 (transcriptome of *Bahd1*-WT and *Bahd1*-KO MEFs).

### RNA extraction and quantification of transcript levels

Total RNA from HEK293-CT and HEK-BAHD1 cells, HEK293-FT cells treated 72h with siRNAs, or from mouse placentas or MEFs was extracted using the RNeasy Kit (Qiagen), from three to six biological replicates. Genomic DNA was removed by treatment with TURBO DNA-freeTM kit (Ambion). cDNAs were generated from 1 to 2 μg total RNA using the RT2-HT first strand kit (Qiagen/SABiosciences). Quantitative Real-Time PCR was performed on Biorad MyiQ device (Biorad), using SsoFast Evagreen supermix (Biorad), as specified by the supplier. Each reaction was performed in triplicate. Data were analyzed by the ΔΔCt method. Target gene expression data were normalized to the relative expression of human *GAPDH* or mouse *Gapdh* (and *Hprt*, for imprinted genes) and *YWHAZ* was used as a control gene. Statistical significance of the difference in mean expression of genes was evaluated using the Student t test; a *P* value <0.05 was considered significant. Primer sets are provided in S1 Text.

### Tandem affinity purification of BAHD1-associated proteins and DNA, nuclear protein and chromatin immunoprecipitations

The TAP-TAG protocol to purify the partners of His_6_-Protein-C-tagged BAHD1 in HPT-BAHD1 cell is described in [11]. Modifications to this protocol, Mass spectrometry analysis and precipitation of the associated DNA are detailed in S1 Text. Immunoprecipitation (IP) of nuclear proteins was performed as described in [70] with the following modifications. Nuclear soluble and insoluble fractions were sonicated, mixed and incubated overnight at 4°C with 1–3μg of the indicated antibodies and then with Dynabeads Protein G (#10004D, Novex) at 4°C during 2h30. IPs were washed 4 times with washing buffer (Tris 20mM pH = 7,65, NaCl 150mM, 0,05% IGEPAL, 2,5% Glycerol, 0,5mM EDTA, 0,6mM DTT). Samples were resuspended in 1X Laemmli and boiled at 95°C for 10 min. Proteins were separated on 8–10% SDS-PAGE gels, transfered to nitrocellulose membrane, probed with primary and secondary antibodies and detected by chemiluminescence (SuperSignal West Femto Substrate #34094, ThermoFisher Scientific). Blots were visualized on Films (Amersham) or using a ChemiDoc MP Imaging system (Bio-Rad). ChIP of modified H3K9 were performed in three independent biological replicates, using the ChIP-IT Express Enzymatic Kit (Active Motif) according to manufacturers instructions. Briefly, HEK293-FT cells were grown in T150 flasks for 72h with either siRNA against BAHD1 or non-targeting control siRNA (up to 70–90% confluency). One flask was kept for RNA extraction and quantification of *BAHD1* knockdown by RT-qPCR. Cells from other flasks were harvested and cross-linked with a final concentration of 1% formaldehyde (Sigma-Aldrich) for 10 minutes. Fixation was stopped with 0.125 M glycine and cells were washed twice with ice-cold PBS. Collected cells were lysed and nuclei pellet were resuspended in shearing cocktail and incubated for digestion for 10 minutes at 37°C. Shearing efficiency was tested by agarose gel electrophoresis and DNA concentration was quantified with a Nanodrop 2000 (ThermoFisher Scientific) to normalize the quantity of chromatin per ChIP. For each ChIP, 3 μg of antibody were used. 10 μL of chromatin was kept as input and processed as ChIP samples. After washing and reverse crosslinking of precipitated samples, DNA was purified by two extractions with equal volumes of phenol:chloroform:isoamylalcohol (25:24:1, pH = 8), assisted by phase lock heavy gel tubes (5Prime), followed by ethanol precipitation. Pellets were washed once in 75% ethanol, then resuspended in 50 μL DNAse-free water. H3K9ac, H3K9me2 and H3K9me3 enrichment levels were measured by qPCR with primers matching in the *ESR1* locus and the non-target control *GAPDH* region (primer sets are provided in S1 Text). A standard curve was generated using 10%, 1%, 0.1% and 0.01% of input DNA. We first determined the fold enrichment of the ChIP sample relative to the IgG sample and then the effect of BAHD1 knockdown on H3K9 modifications was calculated as the ratio of enrichment in cells treated with BAHD1 siRNA to that in cells treated with control siRNA (presented in Fig 6 and S6 Fig as a Log2 ratio).

## Supporting Information

S1 Text Additional methods, references, results and discussion. (PDF)

S1 Fig Blood chemistry on samples collected from *Bahd1*-KO and *Bahd1*-WT mice. **A.** Blood sample analysis of 11–20 week-old *Bahd1*-KO and *Bahd1*-WT mice fasted for 4 hours (n = 5/genotype). **B.** Blood sample analysis of 18 month-old *Bahd1*-KO and *Bahd1*-WT mice fasted for 16 hours (n = 4/genotype). Data are expressed as the mean ± SE (* *P*<0.05; ** *P*<0.01).
(TIF)

S2 Fig Effects of the *Bahd1*-null mutation in the E16.5 placenta and fetal liver. **A.** Histological analysis and *Bahd1*^+/+^ and *Bahd1*^−/−^ E16.5 placentas with Periodic acid-Shiff (PAS) staining at two magnifications (10-fold and 16-fold). Clusters of vacuolated glycogen cells are abundant in WT placentas and severely reduced in *Bahd1*-KO placentas. Hatched lines delineate decidua basalis (Db) and junctional zone (Jz). **B.** Histological analysis and *Bahd1*^+/+^ and *Bahd1*^−/−^ E16.5 livers with hematoxylin and eosin (HE) staining. Magnification 20-fold. Squared regions highlight hepatic cells.
(TIF)

S3 Fig Expression of *BAHD1*, *IGF2* and *MIER1* transcripts in response to induction of *BAHD1* in HPT-BAHD1 cells. RT-qPCR quantification of transcript levels in HPT-BAHD1 cells relative to HPT-Control cells, grown 72h with doxycycline (n = 3 for each condition). Values are normalized by *GAPDH*. Data are expressed as mean ± SD (ns, non-significant; *** *P*<0.001).
(TIF)

S4 Fig Raw images for the western blots used in Fig 4B. Results of the double-affinity purification of HPC-tagged BAHD1 were validated by western blot analysis of inputs and eluates from the 1^st^ and 2^nd^ affinity purifications (E1 and E2) from chromatin extracts of HPT-BAHD1 cells (“BAHD1”) or HPT-control cells (Control). Membranes were often cut in two pieces to detect proteins of different molecular weight, enabling to check sample loading with a common antibody (α-) (e.g. HDAC1, HDAC2, histones H3 or H4). However, this was not always feasible due to the similar molecular weight of a number of proteins of interest, resulting in overlapping hybridization signals in the same region of membranes. We thus had to perform several loadings of the same samples on distinct gels. **A**. Raw images of blots detecting HPC-tagged BAHD1 with Protein C antibodies. H4 was used as a loading control for inputs (same membrane, cut horizontally and probed with H4 antibodies). **B.** Raw images of blots detecting G9a with G9a antibodies. In Fig 4B, E1 and E2 lanes were cut to follow the order of samples used when probing with the other antibodies. FT indicates the column flow through. HDAC2 was used as a loading control (same membrane, cut horizontally). **C.** Raw images of blots detecting KAP1 with KAP1 antibodies. HDAC1 was used as a loading control (same membrane, cut horizontally). **D.** Raw images of blots detecting MIER1 with MIER1 antibodies. HDAC2 was used as a loading control (same membrane, cut horizontally). **E.** Raw images of blots detecting MBD1 with MBD1 antibodies. **F.** Raw images of blots for HDAC1 and HDAC2 using HDAC1 and HDAC2 antibodies, respectively. HDAC1 and HDAC2 having the same size, two different blots were performed. **G.** Raw images of the blots detecting HP1γ with HP1γ antibodies.
(TIF)

S5 Fig Co-immunoprecipitation (IP) assays of BAHD1 with several partners. **A.** Raw images of western blots used in Fig 4E (top panel) before cropping. IP Ctrl corresponds to the use of control IgG in IP assays. After IP of BAHD1-V5 (using BAHD1 antibodies) or MIER1 (using MIER1 antibodies), the membrane was first hybridized with V5 antibodies for detection of BAHD1-V5 (upper panel), then stripped and re-blotted with MIER1 antibodies (lower panel). The intense bands at the bottom of the lower panel are heavy chains of rabbit IgG used for the IP, and detected with rabbit secondary antibodies. Note that the BAHD1 antibody is poorly efficient in immunoprecipitating BAHD1-V5 (top panel, 3^rd^ lane), which can explain why MIER1 is not detected in the BAHD1 IP (bottom panel, 3^rd^ lane). **B.** Raw images of western blots used in Fig 4E (bottom panel) before cropping. After IP of BAHD1-V5 using V5 antibodies, the membranes were probed with V5-HRP (top) or MIER1 (bottom) antibodies. The background signal, which is visible in the IP control, is due to cross-reactivity with IgG and protein G affinity matrix used for the IP. **C.** IPs with IgG Mouse (IP M Ctrl), V5, HP1γ, IgG Rabbit (IP R Crtl) or HDAC2 antibodies were loaded on a membrane that was cut and probed with V5-HRP antibodies (top) or HP1γ antibodies. Note that the V5 immunoblot is the same as in B, before cropping and with a longer exposure time. **D.** After IP with HDAC2 antibodies, the membranes were probed with HDAC2 (top) or MIER1 (bottom) antibodies. On the top, the IP control and IP HDAC2 samples were diluted 1/100 compared to other IPs, since the signal of immunoprecipitated HDAC2 is strong and colocalizes near IgG heavy chains. In C and D, HDAC2 copurifies with low amounts of BAHD1, MIER1 and HP1γ, which might be explained by the fact that HDAC2 belongs to many distinct macromolecular complexes. **E.** IP assays of YFPc-BAHD1 or truncated mutants in HEK293-FT cells. Nuclear extracts from HEK293-FT cells expressing YFPc-BAHD1, YFPc-BAHD1-ΔcPRR or YFPc-BAHD1-ΔBAH were used in IP assays with mouse YFP antibodies followed by immunoblotting with rabbit HDAC2, MIER1 or YFP antibodies or mouse HP1γ antibodies. The last lane shows the HDAC2 blot stripped and reprobed with YFP antibodies. This experiment is a replicate of Fig 4F, except that in Fig 4F we used IgG Veriblot secondary antibodies to minimize the detection of light-chains (IgG) of the HP1γ primary antibody.
(TIF)

S6 Fig BAHD1 depletion alters the patterns of H3K9 acetylation and methylation at the *ESR1* locus. HEK293-FT cells were transfected with control or BAHD1 siRNA in three independent biological replicates (**A, B, C**). We performed one (**A, B**) or two (**C**, left and right histograms) ChIP per antibody and respective mouse or rabbit control IgG, for each modified H3K9. Enrichment of H3K9ac, H3K9me2 and H3K9me3 relative to IgG control at *ESR1* and *GAPDH* regions were estimated by ChIP-qPCR in BAHD1-depleted and control cells (as in Fig 6). Histograms show the relative fold change of ChIP enrichment in cells with BAHD1 siRNA over cells with control siRNA in Log2 ratios. Results of replicate 3 (C) are shown in Fig 6D.
(TIF)

S1 Table *BAHD1* is expressed at a low level in different tissues. (PDF)

S2 Table Blood chemistry in *Bahd1* haplodeficient mice compared to wild type littermates. (PDF)

S3 Table Genes differentially expressed in *Bahd1*-KO compared to *Bahd1*-WT placentas at both E16.5 and E18.5 stages. (PDF)

S4 Table Biological processes associated with genes deregulated in *Bahd1*-KO murine placentas, *Bahd1*-KO MEFs and in human HEK-BAHD1 cells. (PDF)

S5 Table Overlap between *Bahd1*-KO MEFs, *Bahd1*-KO placentas (E18.5) and human HEK-BAHD1 cells transcriptome datasets. (PDF)

S6 Table Tandem-affinity-purification of BAHD1-associated partners. (PDF)

S7 Table Transcription factors predicted to regulate BAHD1-associated genes using the Ingenuity's Upstream Regulator Analysis. (PDF)
