# Supplementary material for: Role of the BAHD1 Chromatin-Repressive Complex in Placental Development and Regulation of Steroid Metabolism
Source: PLoS Genet. 2016 Mar 3;12(3):e1005898. doi: 10.1371/journal.pgen.1005898 (PMC4777444; doi:10.1371/journal.pgen.1005898)
Supplement: S1 Text — (PDF) [file pgen.1005898.s001.pdf]

S1 Text (Supplementary information text)

1. Supplementary Methods
2. Supplementary References
3. Supplementary Results: phenotyping of *Bahd1* heterozygous mice
4. Supplementary Discussion on BAHD1 ChIP

1. Supplementary Methods

1.1. Phenotyping of Bahd1-heterozygous adults
1.2. Phenotyping of Bahd1-knockout adults
1.3. Phenotyping of Bahd1-knockout embryos
1.4. Isolation of primary fibroblasts
1.5. Tandem affinity purification of the HPT-BAHD1-associated proteins and DNA
1.6. Quantitative RT-PCR and ChIP-PCR primers

1.1. Phenotyping of Bahd1-heterozygous adults. The study was performed on 34 mice, 18 *Bahd1*$^{+/-}$ heterozygous (8 males and 10 females) and 16 *Bahd1*$^{+/+}$ wild type littermates (8 males and 8 females). Mice were transferred in the phenotyping facility at the age of 7 weeks, were housed 3 per cage and fed with a standard Chow Diet (CD) (D04, Safe) up until the age of 14 weeks. Then, the diet was switched to a High Fat/High Carbohydrate diet (HFHCD) (RD 12492, Research Diet) for 16 weeks. One HET female died before reaching 30 weeks. Body weights (all mice) and body length (6 mice per group) were recorded once a week from the age of 7 to 30 weeks. General health and sensory-motor abilities were analyzed at the age of 8 weeks, followed by cardiac exploration by echocardiography one week later. Body composition (lean and fat mass, and free body fluid content) was evaluated on conscious mice by quantitative nuclear magnetic resonance (QNMR) at the age of 9 weeks and 23 weeks. Bone mineral density was assessed by dual X-Ray analysis (Dexascan) on anesthetized mice at the age of 28 weeks. An X-Ray analysis of the skeleton was performed at the same time. Blood was collected by retro orbital puncture under isoflurane anaesthesia for biochemistry, hematology and endocrine analysis at the age of 10 and 30 weeks. Histopathological analysis was performed at the age of 30 weeks. Paragenital adipose tissue, kidneys, heart, spleen and liver weights were recorded for 6 mice per group, and among these mice, three *Bahd1*$^{+/-}$ mice and one *Bahd1*$^{+/+}$ mice per gender were processed for histopathological analysis of tissues.

- <u>*General health and sensory-motor functions*</u>

The general health and basic sensory motor functions were evaluated using a modified SHIRPA protocol, providing an overview of physical appearance, body weight, body temperature, neurological reflexes and sensory abilities. To analyze motor function, we employed a Grip test, which measures the maximal muscle strength (g) using an isometric dynamometer connected to a grid (Bioseb). Mice were allowed to grip the grid either with the forepaws, or with both the forepaws and hindpaws, and then they were pulled backwards until they released the grid. Each mouse was submitted to 3 consecutive trials immediately after the modified SHIRPA procedure. The dynamometer recorded the maximal strength developed. Tests were made with the forepaws and the 4 paws.

- <u>*Body composition, Dexascan and X-Ray analysis*</u>

QNMR analysis was performed on mice at the age of 9 and 23-weeks. This procedure is used to give precise analysis of the body composition for fat content, lean tissues and free body fluid. The method uses the Minispec+ analyzer (Bruker) by Nuclear Magnetic Resonance (NMR). The test was conducted during light period on conscious fed mice. Dexascan and X-

Ray analyses were conducted the same day on mice anesthetized with ketamine-xylazine. Bone density scanning, also called dual-energy x-ray absorptiometry (DEXA) was used to measure bone density. The analysis was performed on a pDEXA Sabre from Norland. The analyzer separates bone from tissue, and within the tissue sample, lean and fat mass. A high-resolution radiograph of the mice was performed on a Faxitron Model MX-20. It consists with construction and analysis of digital X-ray pictures with respect to several bones from the head (zygomatic bone, maxilla, mandibles), teeth, scapulae, clavicle, ribs (number, shape, fusion), Pelvis, vertebrae (numbers, shape and potential fusion of cervical, thoracic, lumbar, pelvic, and caudal ones), limb bones (humerus, radius, ulna, femur, tibia…), joints, digits and syndactylism.

- *Echocardiography*

Echocardiography was performed at the age of 9 weeks to explore the cardiac function and anatomy. The following parameters were measured to assess: (*i*) the cardiac anatomy: left ventricular end-diastolic (EDD) and end systolic diameters (ESD), septal (SW) and posterior wall thickness (PW), left ventricular mass (LVM= 1.055x[(EDD+SW+PW)3-EDD3)]); (*ii*) the systolic function: left ventricular fractional shortening (FS) and ejection fraction(EF), and the cardiac output (CO); (*iii*) the diastolic function: the early and late ventricular mitral filling ratio (E/A), the mean deceleration time of the E wave (DTE), duration of A wave (DA) and the isovolumetric relaxation and contraction time (IVRT and IVCT).

- *Blood analysis*

Blood was collected by retro-orbital puncture under isoflurane anesthesia after 4 hours fasting on mice at the age of 10 and 30 weeks (sample from one WT female could not be analysed). A complete blood count was performed on an Advia 120 Vet (Siemens). Blood chemistry was performed on an OLYMPUS AU-400 automated laboratory workstation (Olympus France SA, Rungis, France) using commercial reagent (Olympus Diagnostica GmbH, Lismeehan, Ireland). The following parameters were measured: total cholesterol, HDL and LDL cholesterol, triglycerides, free fatty acids, glycerol and glucose. At the age of 30 weeks additional parameters were measured: total proteins, albumin, urea, creatinin, total bilirubin, bile acids, sodium (Na), potassium (K), chloride (Cl), calcium (Ca), phosphorus (P), magnesium (Mg), alanine aminotransferase (ALAT), alpha-amylase, Alkaline Phosphatase (ALP) and lactic dehydrogenase (LDH). Leptin and insulin levels were measured on a BioPlex analyser (BioRad) using Mouse Endocrine LINCOplex kit (MENDO-75K by Millipore). Adiponectin levels were measured by ELISA using the Quantikine Adiponectin/Acrp30 immunoassay kit (MRP300 R&D system).

- *Histopathology*

Tissues (liver, pancreas, distal ileum and colon, heart, lungs, kidneys, spleen, white paragenital adipose tissue and interscapular brown adipose tissue, and soleus and gastrocnemius muscles) from 3 males and 3 females *Bahd1*$^{+/-}$ and one male and one female *Bahd1*$^{+/+}$ mice were collected, fixed and stored in 10 % formalin and analyzed by hematoxylin and eosin staining.

- *Statistical Analysis*

All the data are expressed as mean ± SE. Statistical analysis were performed using a one way or two ways ANOVA and/or ANOVA repeated measure tests followed by a Fischer's PLSD test with significance set as at $p < 0\ .05$(*), $p < 0.01$(**), $p < 0.0001$ (***).

1.2. Phenotyping of Bahd1-knockout adults. The study was performed on 5 *Bahd1*$^{-/-}$ mice and 5 *Bahd1*$^{+/+}$ control littermates. Body weights and lengths were recorded at different time point between the age of 7 and 17 months. Body composition was evaluated by quantitative magnetic resonance (QNMR) on vigil mice at the age of 16 months. The method uses the

Minispec+ analyzer (Bruker) by Nuclear Magnetic Resonance (NMR). The test was conducted during light period on conscious fed mice. Blood was collected twice at one year of interval (on 7 months old mice after overnight fasting and on 18 months old mice after 4 hours fasting) by retro orbital puncture under isoflurane anaesthesia for the measurement of blood chemistry and blood endocrine parameters. Blood chemistry was performed on an OLYMPUS AU-400 automated laboratory workstation (Olympus France SA, Rungis, France) using commercial reagent (Olympus Diagnostica GmbH, Lismeehan, Ireland. Wako, enzymatic color test for FFA). The following parameters were measured: glucose, urea, creatinine, total proteins, albumin, total cholesterol, HDL cholesterol, triglycerides, free fatty acids (FFA), glycerol, aspartate amino transferase (ASAT) and alanine amino transferase (ALAT). Blood hormones were measured using commercial kits. Adiponectin levels were measured by ELISA using the Quantikine Adiponectin/Acrp30 immunoassay kit (MRP300 R&D system). Leptin and insulin levels were measured on a BioPlex analyser (BioRad) using Mouse Endocrine LINCOplex kit (MENDO-75K by Millipore). Glucagon, GIP, PYY and PP were measured on a BioPlex analyser (BioRad) using Mouse Gut hormone Panel kit (MGT-78K by Millipore). Statistical analysis was as above.

1.3. Phenotyping of Bahd1-knockout embryos. Pregnant females were killed by cervical dislocation. Fetuses were delivered by caesarean section at E16.5 (31 fetuses from 4 pregnant females) and E18.5 (25 fetuses from 3 pregnant females). At E16.5, fetuses were dissected in phosphate buffer saline (PBS). At E18.5, WT fetuses are all already viable: when gently manipulated, they normally start breathing after 5 minutes. The fetuses, kept on the bench under a warm lamp for one hour, were subjected to a brief “viability screen” aimed at determining: if they struggle to breath or remain lethargic, gasp for air or breath regularly, crawl or remain in fetal position, react or not to forceps pinching and if their skin color changes from cyanotic to pink. The body and the placental weights were determined and external defects were recorded. Fetuses were euthanized by a lethal dose of anesthetics (intraperitoneal injection of 30 ml of a ketamine/ xylasine mixture (ketamine: 520 mg/kg; xylasine: 78 mg/kg) and tail biopsies were taken for genotyping by PCR as described in Lebreton et al. (2011). Placenta area and diameter were measured in the Photoshop software (Image analyse menu). For transcriptome analysis at E18.5, the placentas of 25 fetuses were removed and genotypes were performed on tail biopsies of the fetuses (32% *Bahd1*$^{-/-}$, 50% *Bahd1*$^{+/-}$, 18% *Bahd1*$^{+/+}$). The placentas were placed in RNA later at 4°C, frozen in liquid nitrogen and stored at -80°C until RNA extraction. For transcriptome analysis at E16.5, placentas were collected from 57 fetuses and genotypes were performed on tail biopsies of the fetuses (20% *Bahd1*$^{-/-}$, 60% *Bahd1*$^{+/-}$, 20% *Bahd1*$^{+/+}$). The placentas were placed in RNA later at 4°C, frozen in liquid nitrogen and stored at -80°C until RNA extraction. Fetal sex determination was performed by macroscopical observation of the genital organs after liver dissection. For histology, placentas and livers of E16.5 embryos (n=3/genotype) and placentas of E18.5 embryos (n=4/genotype) were collected, fixed and stored in 10 % formalin or Bouin’s fixative until histological processing with hematoxylin & eosin (H&E), or periodic acid Schiff (PAS) staining (Merck, Germany). For PAS staining, 5μm-thick paraffin sections were prepared using standard procedures. Stained sections were digitalized using a slide scanner (Nanozoomer 2.0-HT , *Hamamatsu* , Japan).

1.4. Isolation of primary fibroblasts. Embryos at E13.5 were beheaded and each scissor-minced carcass was digested with trypsin (GibCO/Invitrogen 25200072) diluted half into PBS during 8 minutes at 37°C under agitation. Each embryo was processed independently. The supernatant was then filtered on 70 µm filter (BD Falcon) and digested again with trypsin

diluted 1/4 to obtain a single cell suspension. About 10 millions of cells were plated per 14 cm plate in fibroblast medium (DMEM 4.5 g glucose/L, 10% FCS, gentamicin). Medium was then renewed every 2 to 3 days. Cells were trypsinized just before reaching confluence and frozen in FCS 10% DMSO. To determine the genotype of the cell lines, part of each embryo head was digested in proteinase K containing buffer, and DNA was isolated with Phenol-chloroform extraction. Genotyping by PCR was as described in Lebreton et al. (2011). Primary MEFs were thawed once, amplified for one passage in order to increase the number of cells and to remove non-dividing contaminant cells then used for RNA extraction.

1.5. Tandem affinity purification of the HPT-BAHD1-associated proteins and DNA. We carried out a double-affinity purification of His_6-Protein-C-tagged BAHD1 (HPC-BAHD1) from HPT-BAHD1 cells, and a negative control purification from HPT-CT control cells, starting from 8 g of frozen cellular pellet per cell line. Two independent biological replicates (R1 and R2) were processed and compared to the results of the previous purification (Lebreton et al. 2011). Based on reproducibility and absence in control samples, only the most consistent hits were kept in the final Table S6.

Preparation of nuclear soluble and chromatin fractions and purification of HPC-BAHD1 associated complexes by two-step affinity chromatography using anti-Protein C Affinity Matrix (HPC4, Roche) for the binding of the protein C epitope and Ni-Sepharose High Performance (GE Healthcare) for the binding of the His_6-tag were as described in Lebreton et al. (2011). After overnight binding to the HPC4 resin, bound complexes were extensively washed, and then eluted with TEGN (TGN, 5 mM EGTA, pH 7.65). 200 μL of eluted fractions were used for DNA and protein purification, as described below (*HPC4 purification of DNA and proteins*). The remaining eluates were supplemented with 30 mM imidazole and further affinity-purified on Ni-sepharose. 10 μL of eluates from each purification steps were kept for checking inputs.

- *HPC4 purification of DNA and quantification of enrichement by qPCR.*

DNA was purified by two extractions with equal volumes of phenol:chloroform:isoamylalcohol (25:24:1, pH=8). The phenol phase and interphase from the first extraction were kept for protein extraction. Residual phenol was eliminated from the aqueous phase by extraction with chloroform, then DNA was precipitated by addition of 900 μL of ethanol, 100 μL of ammonium acetate 7.5 M and 0.2 μL of glycogen (20 μg/μL), incubation at -80°C for 30 min and centrifugation at 4°C, 20,000 x g for 15 min. The pellet was washed once in 100 μL of ethanol, then resuspended in 50 μL of pure water for inputs, 25 μL for ChIP samples. ChIP qPCR validation was performed in triplicates on ChIP DNA samples from R1 and R2 purifications. Target enrichments were normalized to the signals obtained with a primer pair hybridizing near *GAPDH*, and to the HPT-control sample.

- *HPC4 purification of proteins.*

Proteins were purified by adding 1.2 ml of pre-chilled methanol, 0.1 M ammonium acetate to each phenolic fraction. After 1 h of precipitation at -80°C, samples were centrifuged 4°C, 20,000 x g for 20. The pellet was washed once in cold methanol-ammonium acetate, once in acetone, dried out and resuspended in 50 μL Laemmli sample buffer 1x. (Fraction **E1**).

- *Nickel-sepharose purification.*

The second affinity column was processed as described in Lebreton et al. (2011). 10 μL of sample buffer 4x were added to 30 μL of eluted fractions (Fraction **E2**).

- *LC-MS-MS*

25 μL of E1 and E2 samples were loaded on a 4-15% Mini-protean TGX gel for brief electrophoresis (1 cm entry of the dye into the gel). After coloration with colloidal Coomassie staining, the whole complex was excised as a single and sent for analysis by microcapillary LC-MS-MS at the Harvard Medical School Taplin Biological Mass Spectrometry facility (https://taplin.med.harvard.edu), as described in Fritsch et al. (2010).

1.6. Quantitative RT-PCR and ChIP-PCR primers

RT-qPCR primers were from Quiagen (mouse *Gapdh*, PPM02946E; mouse *Bahd1*, PPM40834A; human *GAPDH,* PPH00150A; human *BAHD1,* PPH08374A; human *MIER1*, PPH14581B), published studies or designed as follows:

Mouse transcript	Forward sequence	Reverse sequence	Reference
Ywhaz	TAAATGGTCTGTCACCGTCT	GGAAATACTCGGTAGGGTGT	This work
Esr1	CCGCCTTCTACAGGTCTAAT	AGCCAGAGGCATAGTCATTG	This work
Pgr	GCTTGCATGATCTTGTGAAACAGC	GGAAATTCCACAGCCAGTGTCC	This work
Vldlr	CCACAGCAGTATCAGAAGTCAGTGT	CACCTACTGCTGCCATCACTAAGA	This work
Crabp2	TCAGCGTCCAGTGTTCTAGTTG	TCCAGTTGCCAGAAAAGTTAGG	This work
Cxcl14	GAAGATGGTTATCGTCACCACC	CGTTCCAGGCATTGTACCACT	This work
Lepr	GTCTTCGGGGATGTGAATGTC	ACCTAAGGGTGGATCGGGTTT	This work
Osbpl5	TTCTGGGCTGCGAAAATGAG	GTCAGATCCATTGCATAGCCTG	This work
Runx1	GCAGGCAACGATGAAAACTACT	GCAACTTGTGGCGGATTTGTA	This work
Mmp2	ACCTGAACACTTTCTATGGCTG	CTTCCGCATGGTCTCGATG	This work
Lass4	TACCCACATCAGACCCTGAAT	TGAAGTCCTTGCGTTTGACATC	This work
Lpl	GGGAGTTTGGCTCCAGAGTTT	TGTGTCTTCAGGGGTCCTTAG	This work
Aldh2	GACGCCGTCAGCAGGAAAA	CGCCAATCGGTACAACAGC	This work
Cxcl16	CCTTGTCTCTTGCGTTCTTCC	TCCAAAGTACCCTGCGGTATC	This work
Hrpt	CAGGCCAGACTTTGTTGGAT	TTGCGCTCATCTTAGGCTTT	[1]
Igf2	CGCTTCAGTTTGTCTGTTCG	GGGGTGGCACAGTATGTCTC	[1]
H19	TACCTGCCTCAGGAATCTGC	GTTGGCCATGAAGATGGATT	[1]
Cdkn1c	GAAGGACCAGCCTCTCTCG	ACGTTTGGAGAGGGACACC	[1]
Igf2r	GCCTTCAGATTCACAGCACA	TCATGCTTCTGTAACTTGTCATCA	[1]
Dlk1	GAAAGGACTGCCAGCACAAG	CACAGAAGTTGCCTGAGAAGC	[1]
Gtl2	GGACACACGGACACAGACA	TGTCCCACAGGAAATGTGCAA	[1]
Zac1	TTCGTCACCCTGGAGAAGTT	GGTCTGGAGGTGGTTCTTCA	[1]
Grb10	GAGCACGAAGTTTCCGCGCA	CTGGTTGGCTTCTTTGTTGTGG	[1]
Peg3	ACTCACCACTCCGTTGGAGAGTTT	TTTCTCTCCCACTTCGGCTCATGT	[1]
Peg10	GTGGCATCGCAGAGGAAT	GTGAGAGGGGCTTCACTCC	[1]
Snrpn	AGGTCGAGGTCCAGGTCAA	AATCCACCACAGGAACTTGC	[1]
Dcn	GTGTCATCTTCGAGTGGTGC	GTCTAGCAAGGTTGTGTCGG	This work
Gatm	TGATAGTGGGCAGAGCTGAAA	TCTCTTCGACCTCAGCAACA	This work
Htr3	ATGTGGTGGAGAAGATTGCG	GCACATTCCGGCCAAACA	This work
Tfpi2	TCCGTTCTTGGTCTCACTTCA	ACAAGGTCCTGCGTCCAAG	This work
Gpr1	CGCCCTTCTTCCAGTCTCC	TCCATGATGATCTTGTAGGTGAA	This work
**Human transcript**	**Forward sequence**	**Reverse sequence**	**Reference**
GAPDH	GTTCGACAGTCAGCCGCATC	GGAATTTGCCATGGGTGGA	This work
YWHAZ	ACTTTTGGTACATTGTGGCTTCAA	CCGCCAGGACAAACCAGTAT	This work
BAHD1	AGTGTTGCCCCTGTCTGTTA	GAGAGGAAATTCCAACTGGC	This work
ESR1	GGAGGGCAGGGGTGAA	GGCCAGGCTGTTCTTCTTAG	[2]
PGR	CGCGCTCTACCCTGCACTC	TGAATCCGGCCTCAGGTAGTT	This work
AR	CCTGGCTTCCGCAACTTACAC	GGACTTGTGCATGCGGTACTCA	This work
HMGCS1	CATTAGACCGCTGCTATTCTGTC	TTCAGCAACATCCGAGCTAGA	This work

LDLR	CAAAGTCTGCAACATGGCTAGAGA	GTTGTCCAAGCATTCGTTGGTC	[3]
NSDHL	GCGAGCCAATGAGAGACCAA	GAACCGCACCTGGGGATTAT	This work
CRABP2	AGCAGAAGCTCCTGAAGGGA	CCCATCGTTGGTCAGTTCTCT	This work
LASS4	TCGGTCCTGTACCACGAGTC	GCCTGATTAGCAGTGAGAGGTAG	This work
MIER2	CTCGTGAGAGGTCACAGAT	CCGACATGACCCTAGACAA	This work
MIER3	CTGCATGGACAGAAGAAGAA	ATGCTACACATTCAGCAACT	This work

ChIP-qPCR	**Forward sequence**	**Reverse sequence**	**Reference**
ESR1-E1	GAAATCCTTTTCCCCTCTGG	TGGTGCATAAGTGGGAATCA	[4]
ESR1-E2	ACCAAGGACGCTTGCTCTTA	ATTGAAGCCAACCCACAGTC	[4]
ESR1-E3	GAAACAGCCCCAAATCTCAA	TTGTAGCCAGCAAGCAAATG	[4]
ESR1-B1	CTGTGAAGTATGTCACTGGTAG	GAAGAACTACAAAACCCTGCTC	This work
ESR1-B2	GGCTGGGTTACATGGTAATAAG	GTCCTAAAGGTCCATCAATGTC	This work
ESR1-P	GGAGCATTTTGCAGAGGAAG	ATTTAGCAGCTGGGGGAACT	[4]
C6orf211	ACCTGTAATCCCAGCTACTC	GAGTACAACGGCACAATCTC	This work
GAPDH	CGCCTCTCAGCCTTTGAAAGAA	ACGCTTGGATGAAACAGGAGGA	This work

2. Supplementary references

2.1. References for primers
(as indicated in tables above)

1. Radford EJ, Isganaitis E, Jimenez-Chillaron J, Schroeder J, Molla M, Andrews S, et al. An unbiased assessment of the role of imprinted genes in an intergenerational model of developmental programming. PLoS Genet. 2012;8(4):e1002605.

2. Ariazi EA, Clark GM, Mertz JE. Estrogen-related receptor alpha and estrogen-related receptor gamma associate with unfavorable and favorable biomarkers, respectively, in human breast cancer. Cancer Res. 2002;62(22):6510-8.

3. Plosch T, Gellhaus A, van Straten EM, Wolf N, Huijkman NC, Schmidt M, et al. The liver X receptor (LXR) and its target gene ABCA1 are regulated upon low oxygen in human trophoblast cells: a reason for alterations in preeclampsia? Placenta. 2010;31(10):910-8.

4. Carroll JS, Meyer CA, Song J, Li W, Geistlinger TR, Eeckhoute J, et al. Genome-wide analysis of estrogen receptor binding sites. Nat Genet. 2006;38(11):1289-97.

2.2. References supporting a role for ESR1, ESR2, EPAS1 (HIF2α), PPARG, FOS, TP53 and SP1 in placental development and regulation of genes involved in lipid/steroid metabolism

Albrecht ED, Pepe GJ. Estrogen regulation of placental angiogenesis and fetal ovarian development during primate pregnancy. Int J Dev Biol. 2010;54(2-3):397-408.

Barak Y, Sadovsky Y, Shalom-Barak T. PPAR Signaling in Placental Development and Function. PPAR Res. 2008;2008:142082.

Bukovsky A, Caudle MR, Cekanova M, Fernando RI, Wimalasena J, Foster JS, et al. Placental expression of estrogen receptor beta and its hormone binding variant--comparison

with estrogen receptor alpha and a role for estrogen receptors in asymmetric division and differentiation of estrogen-dependent cells. Reprod Biol Endocrinol. 2003;1:36.

Fujimoto J, Nakagawa Y, Toyoki H, Sakaguchi H, Sato E, Tamaya T. Estrogen-related receptor expression in placenta throughout gestation. J Steroid Biochem Mol Biol. 2005;94(1-3):67-9.

Korkmaz C, Sakinci M, Akyol SN, Korgun ET, Ozogul C. Location of Proliferating Cell Nuclear Antigen and p53 Protein in Human First Trimester and Term Placenta. Anal Quant Cytol. 2013;35(6):335-43.

Maltepe E, Krampitz GW, Okazaki KM, Red-Horse K, Mak W, Simon MC, et al. Hypoxia-inducible factor-dependent histone deacetylase activity determines stem cell fate in the placenta. Development. 2005;132(15):3393-403.

Mauvais-Jarvis F. Estrogen and androgen receptors: regulators of fuel homeostasis and emerging targets for diabetes and obesity. Trends in endocrinology and metabolism: TEM. 2011;22(1):24-33.

Rangwala SM, Lazar MA. Peroxisome proliferator-activated receptor gamma in diabetes and metabolism. Trends Pharmacol Sci. 2004;25(6):331-6.

Rankin EB, Rha J, Selak MA, Unger TL, Keith B, Liu Q, et al. Hypoxia-inducible factor 2 regulates hepatic lipid metabolism. Molecular and cellular biology. 2009;29(16):4527-38.

Renaud SJ, Kubota K, Rumi MA, Soares MJ. The FOS transcription factor family differentially controls trophoblast migration and invasion. The Journal of biological chemistry. 2014;289(8):5025-39.

Vaiman D, Calicchio R, Miralles F. Landscape of transcriptional deregulations in the preeclamptic placenta. PloS one. 2013;8(6):e65498.

Solomon SS, Majumdar G, Martinez-Hernandez A, Raghow R. A critical role of Sp1 transcription factor in regulating gene expression in response to insulin and other hormones. Life Sci. 2008;83(9-10):305-12.

Wang X, Zhao X, Gao X, Mei Y, Wu M. A new role of p53 in regulating lipid metabolism. J Mol Cell Biol. 2013;5(2):147-50.

2.3. References for *Mier1*-KO mice phenotypes

- International Mouse Phenotyping Consortium
http://www.mousephenotype.org/data/genes/MGI:1918398

- Mouse Genome Informatics
http://www.informatics.jax.org/allele/genoview/MGI:5450019?counter=1#growth_size_body_id

3. Supplementary Results: phenotyping of *Bahd1* heterozygous mice

- <u>*General health, sensory-motor and cardiac functions*</u>

Bahd1$^{+/-}$ mice had good general health, normal body temperature and no obvious sign of sensory or vestibular function. They also showed normal grip strength. No physiologically relevant differences in cardiac anatomy, systolic function and diastolic function were observed between *Bahd1*$^{+/+}$ and *Bahd1*$^{+/-}$ mice in both genders.

- <u>*Body weight and body composition by QNMR*</u>

Bahd1$^{+/+}$ (WT “contol”) and *Bahd1*$^{+/-}$ (HET “mutant”) mice displayed no significant difference in body weight, body lean and fat content all over the study, both upon CD and HFHCD.

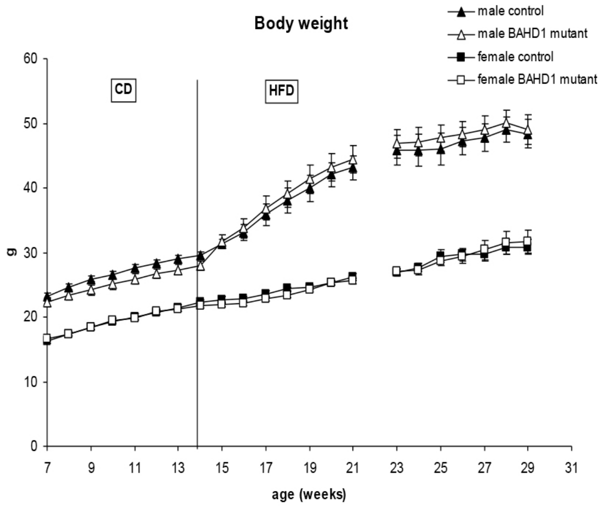

Body Weight

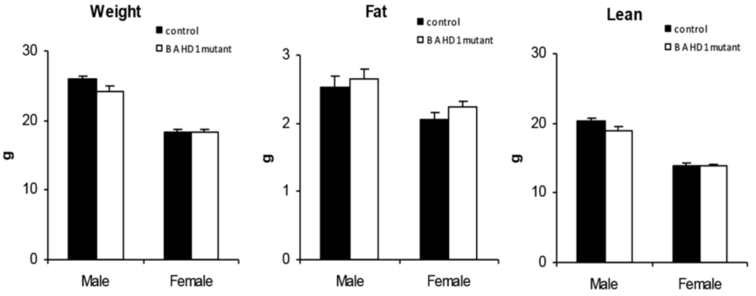

Body composition at 9 weeks of age (upon CD)

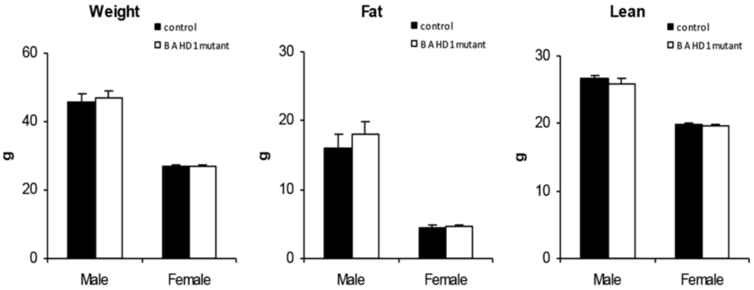

Body composition at 23 weeks of age (upon HFHCD)

- *Dexascan and X-Ray analysis*

Body length, bone area and bone mineral density (BMD) were comparable between HET and WT mice. X-Ray analysis did not revealed any morphological abnormality of the skeleton.

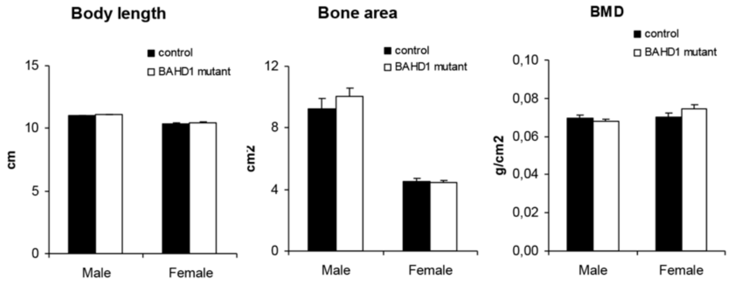

Dexascan analysis at 28 weeks of age

- *Blood analysis*

At the age of 10 weeks upon CD, male HET mice displayed significantly lower glucose levels than WT mice (see **Fig. 1A)**, as well as a slight increase in free fatty acids and in glycerol levels. No change was observed in female HET mice for these parameters. Cholesterol levels were comparable between HET and WT mice (see **Table S2A).**

At the age of 30 weeks, after 26 weeks of HFHCD, most of the clinical chemistry parameters were comparable between HET and WT mice. However, female KO mice displayed lower cholesterol levels and Mg and P levels than the WT mice (see **Fig. 1A, Table S2B**; of note WT #112 and HET #89 samples could not be analyzed).

In addition, insulin, leptin and adiponectin levels in HET and WT were comparable.

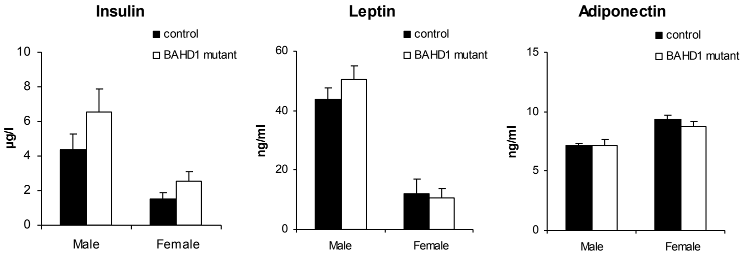

Insulin, leptin and adiponectin at 30 weeks of age.

Whatever the age or the diet, leukocytes, erythrocytes and platelets counts were comparable between HET and WT mice. The percentage of the different leukocytes lineage was not significantly modified in HET mice and no abnormalities were observed in the morphology of leukocytes and erythrocytes.

- *Necropsy and histopathology*

There was no significant macroscopic lesion between HET and WT mice. WT and HET male mice developed a slight hepatic steatosis in the liver mainly composed of macrovacuolization of mid zonal hepatocytes. The presence of hepatic steatosis in male mice is a normal finding upon high fat diet. Females are usually protected against development of this lesion and they usually display no or very little symptoms. Two females HET mice developed minimal hepatocytes degeneration (vacuolization) that should be interpreted with cautious. One HET mouse developed one minimal focus of inflammation in lungs but the number of animals is too low to be significant. We did not observe any other significant microscopic lesion in these mice. In conclusion, we did not detect any significant lesion in HET mice.

4. Supplementary Discussion on BAHD1 ChIP

Until now, the low expression of endogenous BAHD1 has prevented the validation of a ChIP-grade antibody to study BAHD1 binding sites on mammalian genomes. In a recently published work (Libertini et al. 2015), we present the results of a ChIP-seq analysis of the DNA that is pulled-down with ectopically His_6-ProteinC-tagged BAHD1 during affinity purification of protein partners. The results of this native ChIP-seq show that tagged-BAHD1 mainly binds to regulatory regions distant from promoters, such as interspersed repetitive DNA, satellites, enhancers and introns. This is consistent with our observation that when overexpressed, BAHD1 induces massive chromatin compaction (Bierne et al. 2009). In addition, BAHD1 complexes might promote looping interactions between distal DNA elements and target promoters. When intersecting ChIP-seq data with transcriptome analysis of HEK-BAHD1 cells, we found some BAHD1-occupancy sites mapping in regions on or near several BAHD1 candidate target genes. However, these sites were not at the exact same position in the two independent ChIP-seq replicates. This might be due to spreading of BAHD1 on chromatin. It is thus difficult to precisely map the regions where BAHD1 initially binds, at least in cells overexpressing tagged-BAHD1. We believe that searching for BAHD1 direct targets requires additional optimization steps in order to immunoprecipitate endogenous BAHD1 from formaldehyde-fixed cells.
