# Supplementary figures and images for: Role of the BAHD1 Chromatin-Repressive Complex in Placental Development and Regulation of Steroid Metabolism
Source: PLoS Genet. 2016 Mar 3;12(3):e1005898. doi: 10.1371/journal.pgen.1005898 (PMC4777444; doi:10.1371/journal.pgen.1005898)

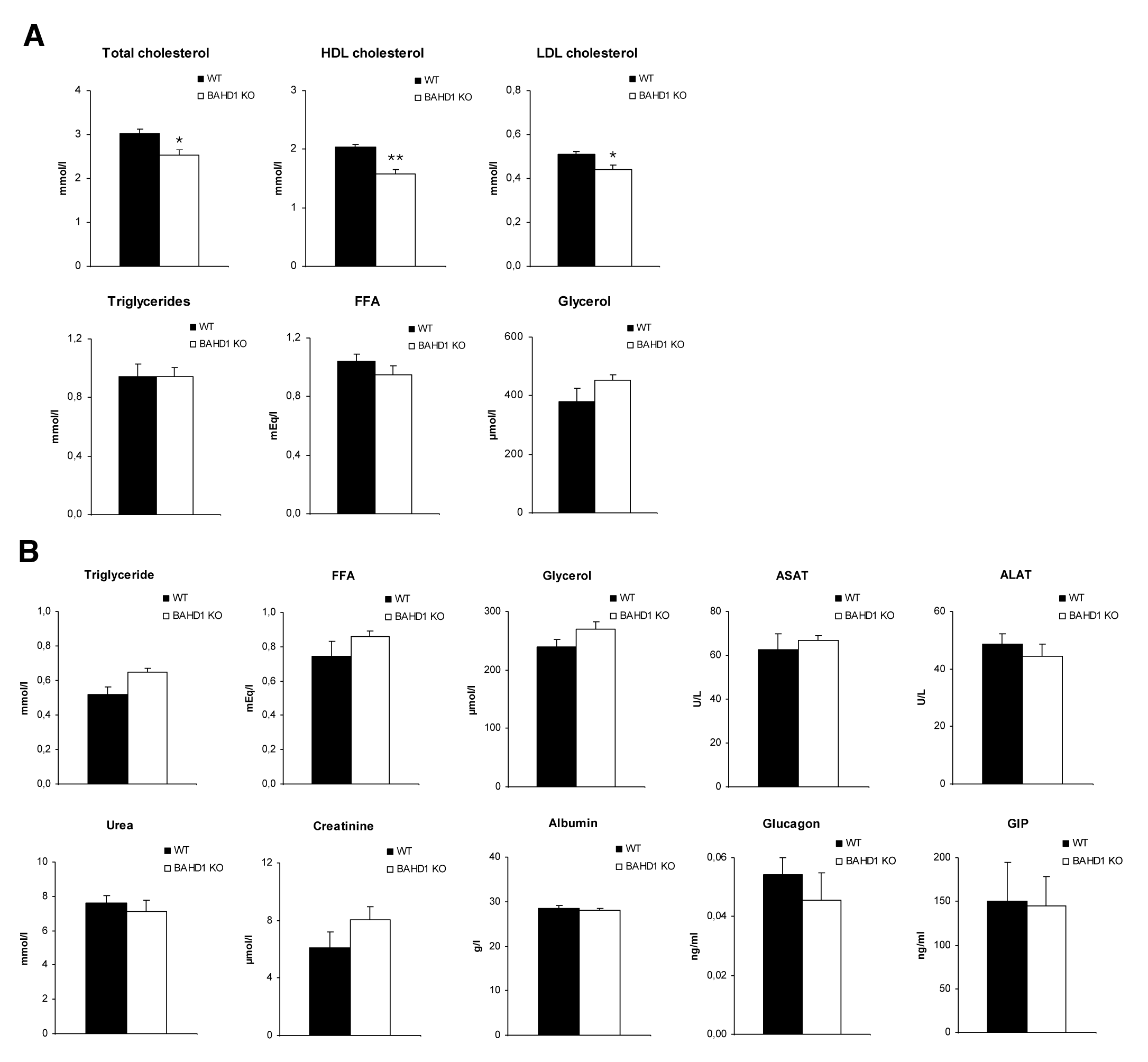

Supplement: S1 Fig — A. Blood sample analysis of 11–20 week-old Bahd1-KO and Bahd1-WT mice fasted for 4 hours (n = 5/genotype). B. Blood sample analysis of 18 month-old Bahd1-KO and Bahd1-WT mice fasted for 16 hours (n = 4/genotype). Data are expressed as the mean ± SE (* P<0.05; ** P<0.01). (TIF) [file pgen.1005898.s002.tif]

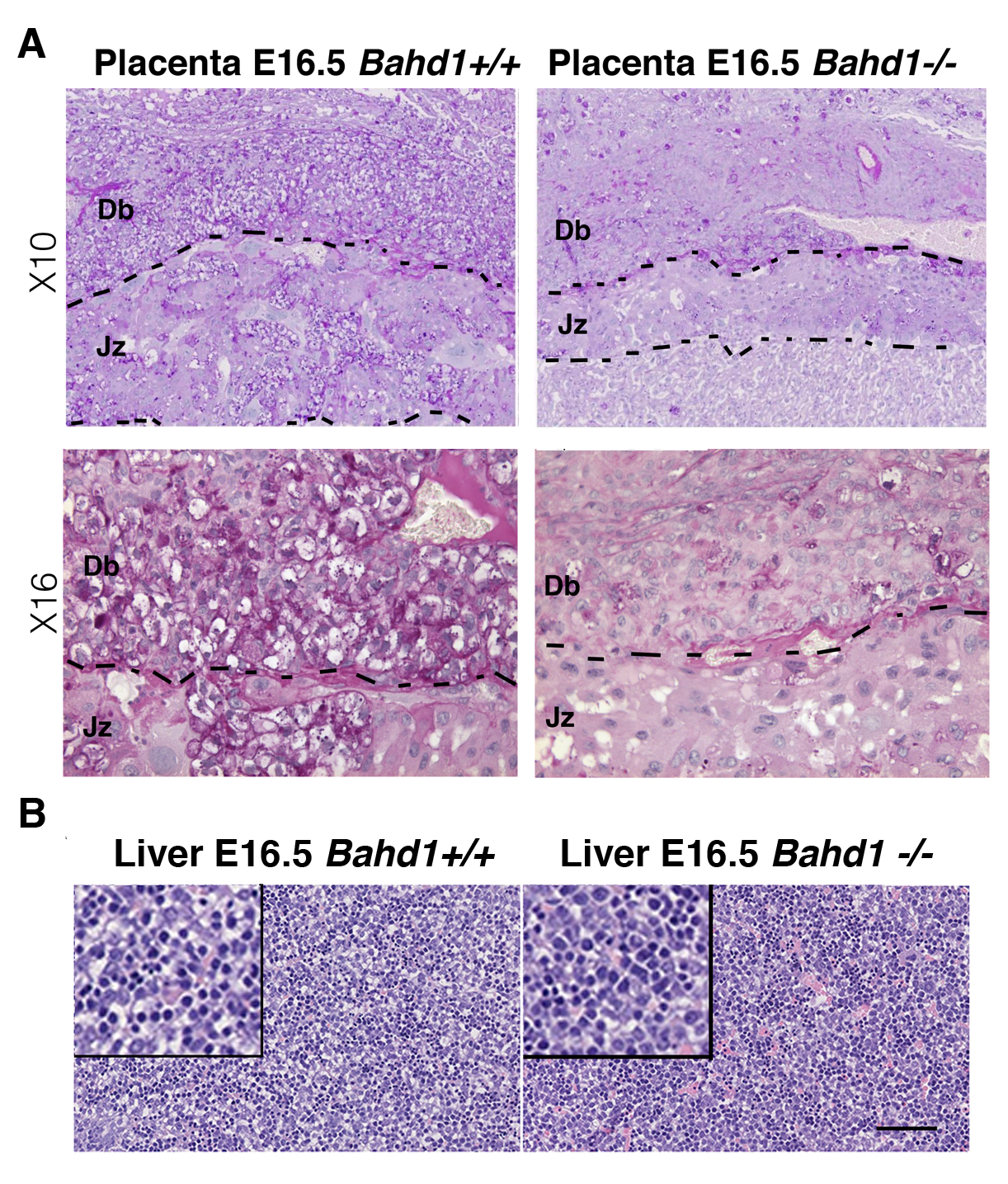

Supplement: S2 Fig — A. Histological analysis and Bahd1+/+ and Bahd1−/− E16.5 placentas with Periodic acid-Shiff (PAS) staining at two magnifications (10-fold and 16-fold). Clusters of vacuolated glycogen cells are abundant in WT placentas and severely reduced in Bahd1-KO placentas. Hatched lines delineate decidua basalis (Db) and junctional zone (Jz). B. Histological analysis and Bahd1+/+ and Bahd1−/− E16.5 livers with hematoxylin and eosin (HE) staining. Magnification 20-fold. Squared regions highlight hepatic cells. (TIF) [file pgen.1005898.s003.tif]

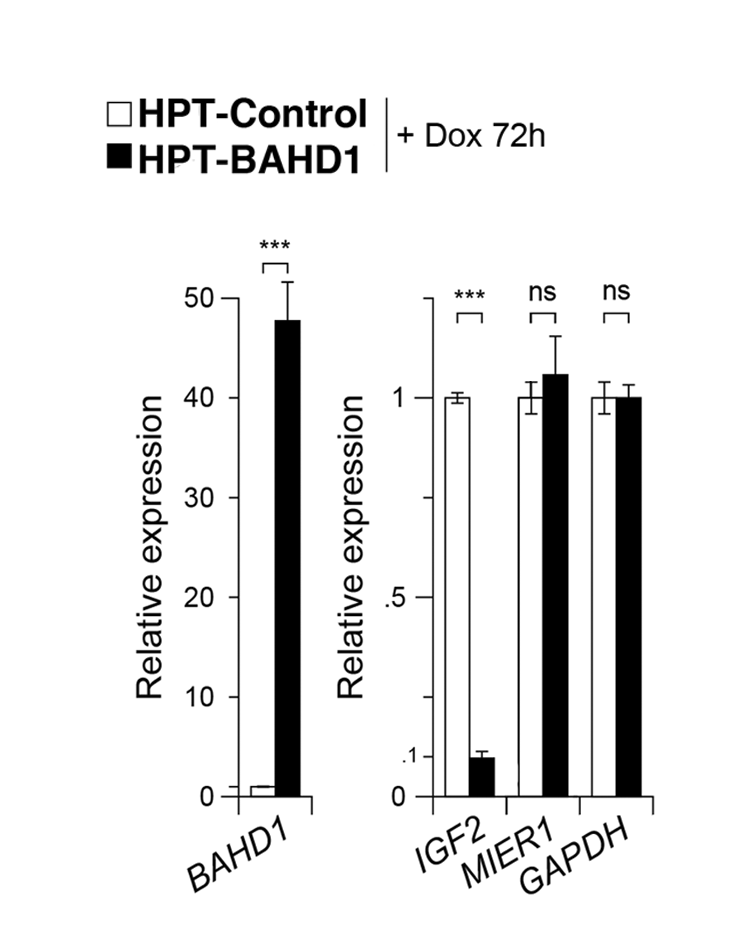

Supplement: S3 Fig — RT-qPCR quantification of transcript levels in HPT-BAHD1 cells relative to HPT-Control cells, grown 72h with doxycycline (n = 3 for each condition). Values are normalized by GAPDH. Data are expressed as mean ± SD (ns, non-significant; *** P<0.001). (TIF) [file pgen.1005898.s004.tif]

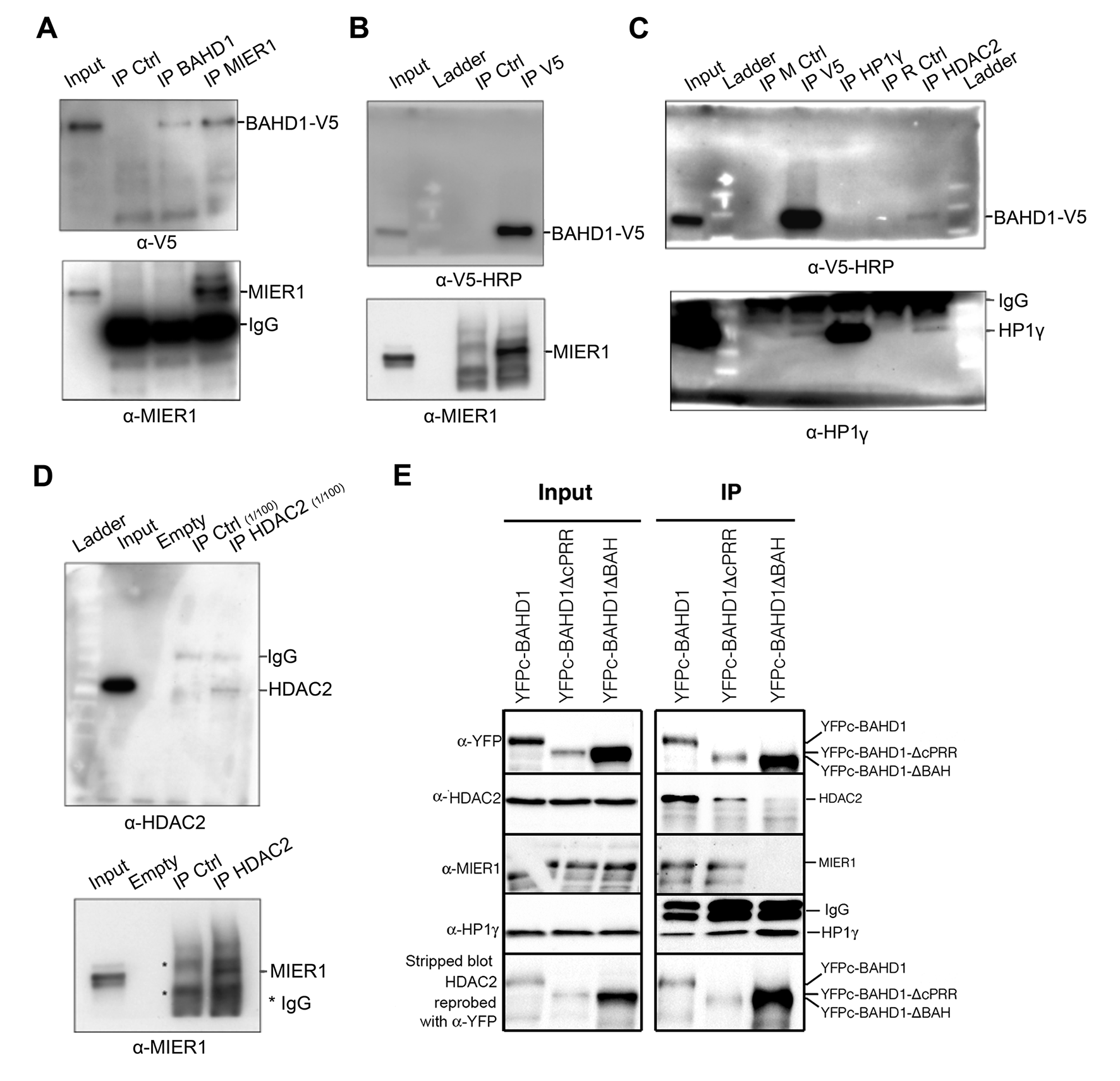

Supplement: S5 Fig — A. Raw images of western blots used in Fig 4E (top panel) before cropping. IP Ctrl corresponds to the use of control IgG in IP assays. After IP of BAHD1-V5 (using BAHD1 antibodies) or MIER1 (using MIER1 antibodies), the membrane was first hybridized with V5 antibodies for detection of BAHD1-V5 (upper panel), then stripped and re-blotted with MIER1 antibodies (lower panel). The intense bands at the bottom of the lower panel are heavy chains of rabbit IgG used for the IP, and detected with rabbit secondary antibodies. Note that the BAHD1 antibody is poorly efficient in immunoprecipitating BAHD1-V5 (top panel, 3rd lane), which can explain why MIER1 is not detected in the BAHD1 IP (bottom panel, 3rd lane). B. Raw images of western blots used in Fig 4E (bottom panel) before cropping. After IP of BAHD1-V5 using V5 antibodies, the membranes were probed with V5-HRP (top) or MIER1 (bottom) antibodies. The background signal, which is visible in the IP control, is due to cross-reactivity with IgG and protein G affinity matrix used for the IP. C. IPs with IgG Mouse (IP M Ctrl), V5, HP1γ, IgG Rabbit (IP R Crtl) or HDAC2 antibodies were loaded on a membrane that was cut and probed with V5-HRP antibodies (top) or HP1γ antibodies. Note that the V5 immunoblot is the same as in B, before cropping and with a longer exposure time. D. After IP with HDAC2 antibodies, the membranes were probed with HDAC2 (top) or MIER1 (bottom) antibodies. On the top, the IP control and IP HDAC2 samples were diluted 1/100 compared to other IPs, since the signal of immunoprecipitated HDAC2 is strong and colocalizes near IgG heavy chains. In C and D, HDAC2 copurifies with low amounts of BAHD1, MIER1 and HP1γ, which might be explained by the fact that HDAC2 belongs to many distinct macromolecular complexes. E. IP assays of YFPc-BAHD1 or truncated mutants in HEK293-FT cells. Nuclear extracts from HEK293-FT cells expressing YFPc-BAHD1, YFPc-BAHD1-ΔcPRR or YFPc-BAHD1-ΔBAH were used in IP assays with mouse YFP a [file pgen.1005898.s006.tif]

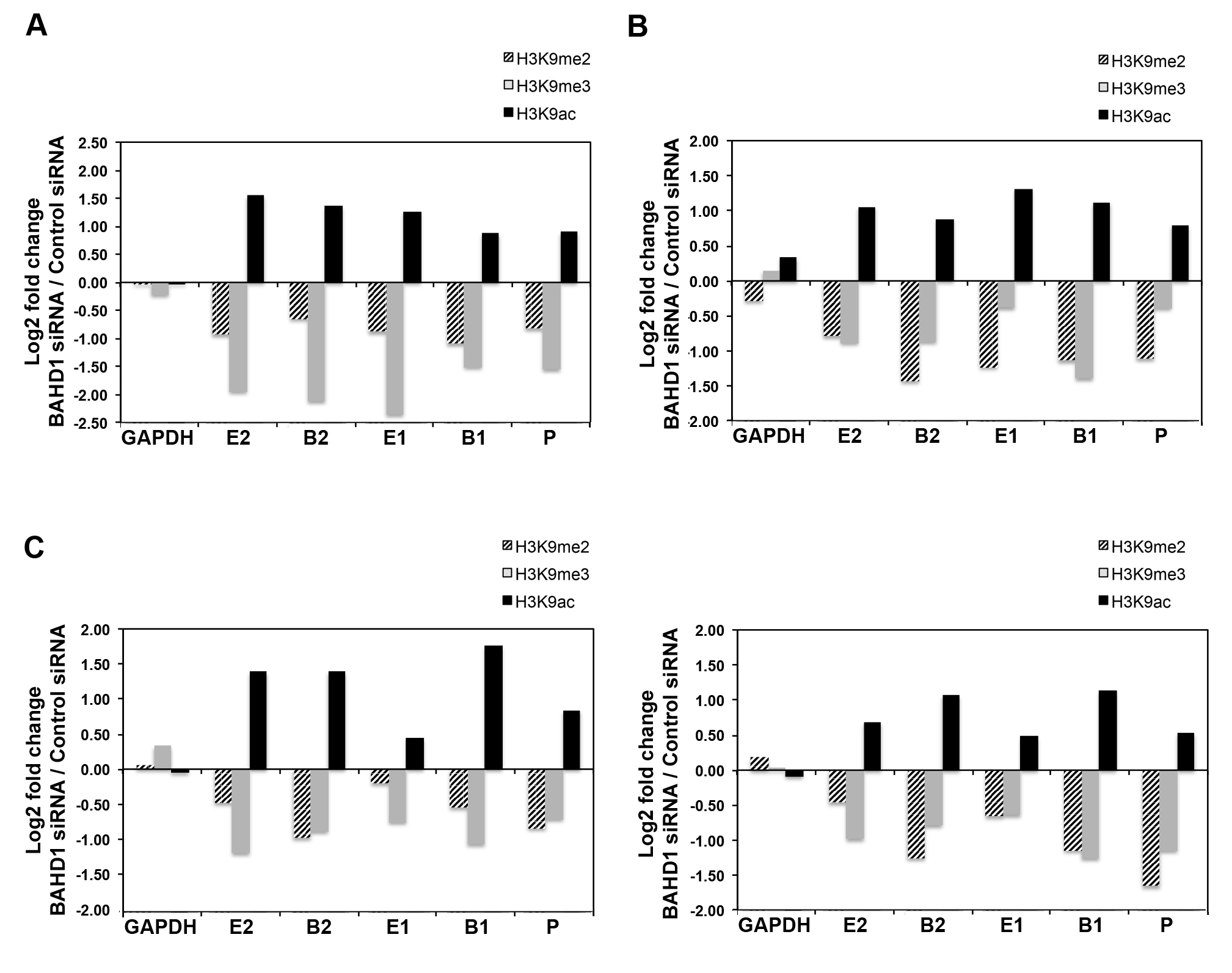

Supplement: S6 Fig — HEK293-FT cells were transfected with control or BAHD1 siRNA in three independent biological replicates (A, B, C). We performed one (A, B) or two (C, left and right histograms) ChIP per antibody and respective mouse or rabbit control IgG, for each modified H3K9. Enrichment of H3K9ac, H3K9me2 and H3K9me3 relative to IgG control at ESR1 and GAPDH regions were estimated by ChIP-qPCR in BAHD1-depleted and control cells (as in Fig 6). Histograms show the relative fold change of ChIP enrichment in cells with BAHD1 siRNA over cells with control siRNA in Log2 ratios. Results of replicate 3 (C) are shown in Fig 6D. (TIF) [file pgen.1005898.s007.tif]
