# Supplementary material for: Role of the BAHD1 Chromatin-Repressive Complex in Placental Development and Regulation of Steroid Metabolism
Source: PLoS Genet. 2016 Mar 3;12(3):e1005898. doi: 10.1371/journal.pgen.1005898 (PMC4777444; doi:10.1371/journal.pgen.1005898)
Supplement: S1 Table — (PDF) [file pgen.1005898.s008.pdf]

Table S1. BAHD1 is expressed at a low levels in different tissues.

The NextBio Body Atlas (https://www.nextbio.com) provides a rank-ordered analysis of gene expression across all normal tissues available in the Body Atlas biosets are drawn from all available RNA expression studies that used Affymetrix U133 Genechip© Arrays for human studies.
Data corresponding to 128 human tissues were incorporated from 1,067 arrays.
BAHD1 mRNA levels were compared to that of a selection of referenced genes
(either classical housekeeping genes or tissue-specific genes or genes selected because of the range in which they fell).

Gene	Lowest tissue expression observed	Highest tissue expression observed	Fold-Difference
ASCL2	68	138	2
RASAL2	106	638	6
BAHD1	521	1840	3.5
CDC73	160	2330	14.6
HPRT1	433	7870	18.2
VDR	390	10100	25.9
PPIF	652	11800	18.1
NRCAM	184	18700	101.6
RPL32	10100	86100	8.5
ALB	103	111000	1077.7
UBB	21700	166000	7.7
GAPDH	6520	166000	25.5

e database.
