# Supplementary material for: Role of the BAHD1 Chromatin-Repressive Complex in Placental Development and Regulation of Steroid Metabolism
Source: PLoS Genet. 2016 Mar 3;12(3):e1005898. doi: 10.1371/journal.pgen.1005898 (PMC4777444; doi:10.1371/journal.pgen.1005898)
Supplement: S2 Table — (PDF) [file pgen.1005898.s009.pdf]

Table S2. Blood chemistry in *Bahd1*-haplodeficient mice compared to wild type littermates.

Mean values and significant differences are highligted in grey and pink boxes, respectively.

(T. Chol.: total cholesterol; HDL: high-density lipoprotein; LDL: low-density lipoprotein; TG: triglycerides; FFA: free fatty acids
Na:Sodium; K: potassium; Cl: chloride; Ca: calcium; P: phosphorus; LDH: lactacte dehydrogenase;
ALAT: alanine amino transferase; ASAT: aspartate amino transferase; ALP: alkaline phosphatase)

Table S2A. Blood chemistry on 14 week-old *Bahd1+/-* and *Bahd1 +/+* mice fed regular chow diet.

sexe and genotype	mouse #	Glucose mmol/l	T. Chol. mmol/l	HDL Chol. mmol/l	LDL Chol. mmol/l	TG mmol/l	FFA mEq/l	Glycerol µmol/l	Adiponectin µg/ml	Insulin µg/l
	77	20.4	2.21	1.52	0.37	0.68	1.12	283	5.23	3.30
	78	18.0	2.50	1.64	0.42	0.99	1.21	311	4.93	0.53
	86	17.3	2.72	1.60	0.57	0.77	0.99	252	4.67	2.07
Male	87	22.4	2.81	1.69	0.67	1.03	0.97	256	4.96	0.93
Bahd1+/+	88	22.4	2.50	1.58	0.48	0.85	1.04	292	7.34	2.03
	96	22.0	2.20	1.51	0.44	1.15	1.31	363	5.41	6.95
	106	18.8	2.32	1.47	0.44	1.00	1.30	358	7.20	4.01
	107	21.3	2.37	1.51	0.47	1.04	1.26	362	5.70	5.47
	61	13.2	2.85	1.85	0.48	0.96	1.35	448	6.23	1.18
	69	14.1	2.17	1.31	0.29	1.15	1.95	524	6.51	3.66
	75	22.4	2.15	1.38	0.41	0.80	1.21	320	7.16	2.97
Male	76	19.3	2.48	1.72	0.41	0.82	1.23	316	6.67	< 0,04
Bahd1+/-	84	12.0	2.98	1.80	0.66	1.00	1.29	445	6.17	1.45
	85	14.6	2.42	1.46	0.51	0.97	1.35	435	7.76	1.44
	95	15.1	2.53	1.53	0.52	1.00	1.38	409	5.30	2.96
	114	16.4	2.43	1.60	0.41	0.84	1.22	297	7.54	2.44
**mean Male WT**		**20.3**	**2.45**	**1.57**	**0.48**	**0.94**	**1.15**	**310**	**5.68**	**3.16**
sem Male WT		0.7	0.08	0.03	0.03	0.06	0.05	16	0.36	0.79
**mean Male HET**		**15.9**	**2.50**	**1.58**	**0.46**	**0.94**	**1.37**	**399**	**6.67**	**2.30**
sem Male HET		1.2	0.10	0.07	0.04	0.04	0.09	28	0.28	0.34
t-test (p)		*0.0073*	*0.7205*	*0.8305*	*0.6836*	*0.9576*	*0.0408*	*0.0161*	*0.0509*	*0.3616*
	93	14.5	2.34	1.34		0.62	1.18	323	9.49	0.72
	94	15.2	2.10	1.29	0.42	0.76	1.33	406	8.77	1.15
	99	20.2	2.45	1.43	0.56	0.82	1.16	304	6.61	0.41
Female	101	20.5	1.91	1.11	0.40	0.66	1.13	307	9.64	4.48
Bahd1+/+	102	12.8	2.32	1.23	0.63	0.76	1.16	352	9.56	2.10
	103	16.4	2.33	1.34	0.55	0.79	1.07	312	9.22	5.22
	109	14.5	2.39	1.25	0.66	0.93	1.30	353	7.47	1.07
	112	16.7	1.98	1.19	0.42	0.84	1.36	382	6.20	1.72
	89	15.1	1.86	0.99	0.57	0.67	1.24	354	9.39	3.44
	90	17.3	2.09	1.15	0.56	0.78	1.01	284	9.25	3.26
	91	17.6	2.34	1.39	0.50	0.76	1.02	275	10.57	4.78
	92	13.6	2.11	1.25	0.46	0.79	1.40	417	10.73	< 0,04
Female	98	18.6	2.05	1.18	0.47	0.99	1.42	379	8.80	3.77
Bahd1+/-	100	16.1	2.18	1.23	0.46	0.71	1.25	357	10.69	2.68
	104	16.7	2.08	1.24	0.49	0.76	1.19	315	5.43	1.16
	108	19.5	1.81	1.06	0.43	0.58	1.00	280	9.01	4.49
	110	16.3	2.18	1.17	0.58	0.87	1.17	312	6.74	1.45
	111	19.1	2.31	1.28	0.61	0.70	1.19	315	5.46	1.41
**mean Female WT**		**16.3**	**2.23**	**1.27**	**0.52**	**0.77**	**1.21**	**342**	**8.37**	**2.11**
sem Female WT		1.0	0.07	0.04	0.04	0.03	0.04	13	0.50	0.63
**mean Female HET**		**17.0**	**2.10**	**1.19**	**0.51**	**0.76**	**1.19**	**329**	**8.61**	**2.94**
sem Female HET		*0.6*	*0.05*	*0.04*	*0.02*	*0.04*	*0.05*	*15*	*0.64*	*0.43*
t-test (p)		*0.5586*	*0.1679*	*0.1427*	*0.8662*	*0.8226*	*0.7265*	*0.5159*	*0.7836*	*0.2938*

Table S2B. Blood chemistry on 30 week-old *Bahd1+/-* and *Bahd1+/+* mice fed regular chow diet for 14 weeks followed by high fat/high carbohydrat diet for 16 weeks.

sexe and genotype	mouse #	Glucose mmol/l	T. Chol. mmol/l	HDL Chol. mmol/l	LDL Chol. mmol/l	TG mmol/l	FFA mEq/l	Glycerol µmol/l	Adiponectin µg/ml	Insulin µg/l	Urea mmol/l	Na mmol/l	K mmol/l	Cl mmol/l	T. proteins g/l	Albumin g/l	Ca mmol/l	P mmol/l	Mg mmol/l	T. bilirubin µmol/l	LDH U/l	ALAT U/l	ALP U/l	a-Amylase U/l	Bile acids µmol/l	Creatinine µmol/l	Leptin ng/ml
	77	24.6	5.24	3.64	1.36	0.82	1.05	347	6.81	2.34	6.4	145	4.3	108	50	27	2.25	2.92	0.82	1.8	517	81	56	827	0.9	6.4	42.30
	78	21.6	7.03	4.52	2.01	0.90	1.26	422	6.93	4.83	7.2	146	5.3	111	54	29	2.29	2.88	0.87	2.0	879	160	73	862	2.9	7.8	41.29
	86	insufficient	insufficient	insufficient	insufficient	insufficient	insufficient	insufficient	4.31	4.28	insufficient	146	4.3	112	insufficient	insufficient	2.51	insufficient	0.93	insufficient	559	insufficient	insufficient	insufficient	insufficient	insufficient	outlier
Male	87	19.4	4.89	3.47	1.26	1.25	1.40	457	7.29	7.61	5.4	146	4.4	108	52	27	2.38	2.90	0.89	1.8	842	89	80	811	1.9	6.9	51.25
Bahd1+/+	88	17.0	5.43	3.70	1.41	0.90	1.29	461	7.41	8.22	6.8	146	5.0	110	54	28	2.31	2.58	0.85	1.5	787	112	78	870	5.7	7.4	53.66
	96	19.1	5.06	3.51	1.24	0.87	1.24	414	8.09	2.55	9.7	142	5.0	106	52	27	2.35	3.37	0.93	1.4	624	78	50	861	4.2	8.8	50.34
	106	17.1	4.00	2.89	0.93	0.97	1.12	310	7.11	0.94	8.5	147	4.4	112	48	25	2.24	3.02	0.91	2.1	737	53	45	735	9.2	7.2	24.07
	107	21.3	3.80	2.70	0.78	1.53	1.29	396	6.51	4.01	7.4	146	4.0	111	50	26	2.29	2.86	0.91	2.0	514	41	47	677	2.2	7.5	44.47
	61	15.4	5.18	3.71	1.20	0.97	1.24	404	5.86	7.44	6.8	147	4.8	108	51	28	2.35	2.89	0.90	2.0	694	99	51	808	1.2	7.3	59.39
	69	20.3	4.25	3.07	0.93	1.28	1.17	404	9.20	3.92	6.8	143	4.5	111	49	27	2.31	2.75	0.80	2.2	501	63	38	773	1.0	6.4	60.82
	75	15.7	5.53	3.78	1.37	1.37	1.72	492	6.59	9.32	6.6	149	4.6	111	51	28	2.30	2.37	0.81	2.5	639	140	57	736	1.9	7.6	63.04
Male	76	20.1	6.65	4.34	1.89	1.24	1.40	455	7.48	9.83	5.6	147	4.9	109	53	29	2.32	2.63	0.80	2.0	769	162	77	710	2.4	7.9	46.89
Bahd1+/-	84	16.6	5.01	3.48	1.22	0.97	1.33	428	7.42	4.03	6.2	145	4.7	109	50	27	2.34	2.94	0.94	1.6	1185	198	48	822	2.0	7.1	46.83
	85	21.0	4.77	3.26	1.28	0.88	1.23	376	8.31	1.53	6.9	148	4.2	112	50	26	2.33	2.43	0.88	2.0	426	47	52	2215	7.2	5.9	29.51
	95	insufficient	3.18	2.25	0.67	0.60	insufficient	insufficient	insufficient	insufficient	insufficient	151	4.9	113	insufficient	insufficient	insufficient	4.00	insufficient	1.5	882	79	42	640	insufficient	10.2	insufficient
	114	13.1	6.03	3.97	1.62	1.03	1.51	475	5.32	9.92	7.3	149	5.4	109	54	30	2.44	3.89	0.89	1.6	862	142	72	807	1.8	8.8	47.92
**mean Male WT**		**20.0**	**5.06**	**3.49**	**1.28**	**1.03**	**1.24**	**401**	**6.81**	**4.35**	**7.3**	**145.50**	**4.58**	**109.75**	**51.43**	**27.00**	**2**	**2.93**	**0.89**	**1.8**	**682.4**	**87.71**	**61.29**	**806.14**	**3.85**	**7.43**	**44**
sem Male WT		1.0	0.40	0.22	0.15	0.10	0.04	21	0.39	0.90	0.5	0.53	0.16	0.77	0.84	0.49	0	0.09	0.01	0.1	52.2	14.92	5.76	27.78	1.07	0.29	4
**mean Male HET**		**17.4**	**5.08**	**3.48**	**1.27**	**1.04**	**1.37**	**433**	**7.17**	**6.57**	**6.6**	**147.38**	**4.75**	**110.25**	**51.14**	**27.86**	**2**	**2.99**	**0.86**	**1.925**	**744.8**	**116.25**	**54.63**	**938.88**	**2.49**	**7.65**	**51**
sem Male HET		1.1	0.38	0.23	0.13	0.09	0.07	16	0.51	1.28	0.2	0.89	0.12	0.62	0.67	0.51	0	0.22	0.02	0.12	84.7	18.57	4.83	183.57	0.81	0.48	4
t-test (p)		*0.1199*	*0.9848*	*0.9816*	*0.9538*	*0.9512*	*0.1357*	*0.2430*	*0.5819*	*0.1706*	*0.2177*	*0.0913*	*0.4022*	*0.6217*	*0.7950*	*0.2472*	*0.7126*	*0.8311*	*0.2675*	*0.4473*	*0.5407*	*0.2614*	*0.3880*	*0.5162*	*0.3323*	*0.7068*	*0.2689*
	93	15.4	3.08	2.19	0.72	0.67	1.50	398	9.64	0.44	8.8	146	4.2	112	52	30	2.32	3.00	0.94	2.1	487	29	78	588	4.7	9.3	6.04
	94	16.9	3.53	2.57	0.76	0.62	1.34	381	10.76	0.55	8.6	149	4.2	113	52	30	2.40	3.59	1.00	2.2	363	30	80	732	2.7	9.2	34.59
	99	16.5	2.71	1.84	0.63	0.94	1.51	458	8.92	2.95	7.0	148	3.6	113	53	29	2.37	2.88	0.83	1.8	524	37	50	580	1.5	9.1	17.93
Female	101	12.5	2.60	1.59	0.79	0.22	0.42	168	8.34	0.67	6.6	151	3.6	115	54	25	2.41	3.61	0.92	2.5	426	24	41	444	5.8	10.4	2.66
Bahd1+/+	102	16.3	2.95	2.04	0.71	0.55	1.05	332	9.31	1.44	6.5	145	4.0	112	51	29	2.37	2.57	0.84	2.9	598	40	80	582	9.2	8.0	6.37
	103	13.9	2.76	1.87	0.68	0.30	0.76	243	10.47	1.83	7.7	149	4.4	115	50	28	2.34	3.74	0.97	1.7	699	22	57	504	2.6	10.5	4.67
	109	16.2	2.76	1.89	0.64	0.62	1.27	329	7.83	2.61	7.7	148	3.6	115	51	27	2.33	2.79	0.96	2.1	418	27	63	563	2.9	9.1	outlier
	112	out of range	insufficient	insufficient	insufficient	insufficient	insufficient	insufficient	insufficient	insufficient	out of range	out of range	out of range	out of range	out of range	out of range	out of range	out of range	out of range	out of range	insufficient	insufficient	insufficient	insufficient	out of range	out of range	insufficient
	89	dead	dead	dead	dead	dead	dead	dead	dead	dead	dead	dead	dead	dead	dead	dead	dead	dead	dead	dead	dead	dead	dead	dead	dead	dead	dead
	90	18.9	2.25	1.39	0.61	0.91	1.35	384	6.96	0.94	6.3	143	3.4	111	46	25	2.26	2.50	0.84	2.5	412	33	54	511	2.4	8.4	2.00
	91	17.2	2.17	1.49	0.46	0.76	1.29	390	6.74	1.43	8.5	147	3.6	114	49	28	2.34	2.57	0.83	1.8	508	38	56	466	4.1	8.3	5.88
	92	13.5	2.08	1.35	0.45	0.44	1.04	305	insufficient	4.03	7.1	146	4.5	115	50	28	2.33	2.28	0.89	1.5	1600	206	56	507	2.2	9.5	3.84
Female	98	17.1	3.37	2.27	0.77	0.83	1.41	388	8.93	0.98	8.3	146	4.0	112	54	30	2.39	2.66	0.86	1.8	404	28	65	527	2.8	8.5	21.62
Bahd1+/-	100	16.2	2.70	1.65	0.73	0.77	1.34	389	8.92	2.57	6.5	144	4.4	111	53	28	2.34	2.54	0.93	1.4	1851	224	60	475	3.1	9.5	1.33
	104	16.8	2.59	1.80	0.56	0.57	1.33	389	9.88	0.53	8.4	148	4.3	113	50	29	2.41	3.29	0.91	1.5	438	21	59	502	5.4	8.5	17.97
	108	19.6	2.89	2.08	0.59	1.01	1.55	476	10.00	5.48	7.8	144	3.6	111	53	28	2.39	1.99	0.74	1.8	358	26	69	609	1.5	7.6	20.61
	110	16.7	2.76	1.94	0.63	0.67	1.33	373	9.25	4.35	7.7	146	3.6	114	52	29	2.35	2.02	0.75	2.3	418	19	63	548	1.8	7.6	3.55
	111	13.8	2.64	1.81	0.57	1.10	2.19	583	9.17	2.37	7.8	153	3.9	118	55	30	2.43	2.90	0.83	2.1	427	23	58	522	1.8	9.8	19.50
**mean Female WT**		**15.4**	**2.91**	**2.00**	**0.70**	**0.56**	**1.12**	**330**	**9.32**	**1.50**	**7.6**	**148.00**	**3.95**	**113.57**	**51.86**	**28.29**	**2**	**3.17**	**0.92**	**2.2**	**502.14**	**29.86**	**64.14**	**570.43**	**4.19**	**9**	**12.04**
sem Female WT		0.6	0.12	0.12	0.02	0.09	0.15	37	0.40	0.38	0.3	0.76	0.13	0.53	0.51	0.68	0	0.18	0.02	0.2	43.88	2.48	5.94	33.48	0.99	0	5.01
**mean Female HET**		**16.6**	**2.61**	**1.75**	**0.60**	**0.78**	**1.43**	**409**	**8.73**	**2.52**	**7.6**	**146.33**	**3.91**	**113.22**	**51.33**	**28.33**	**2**	**2.53**	**0.84**	**1.9**	**712.89**	**68.67**	**60.00**	**518.56**	**2.77**	**9**	**10.70**
sem Female HET		0.7	0.13	0.10	0.04	0.07	0.11	26	0.44	0.58	0.3	0.99	0.13	0.78	0.94	0.50	0	0.14	0.02	0.1	192.94	27.76	1.62	14.07	0.42	0	2.96
t-test (p)		*0.2099*	*0.1190*	*0.1426*	*0.0319*	*0.0652*	*0.1136*	*0.0941*	*0.3404*	*0.1919*	*0.9220*	*0.2226*	*0.8305*	*0.7325*	*0.6600*	*0.9547*	*0.9012*	*0.0113*	*0.0278*	*0.1168*	*0.3623*	*0.2425*	*0.4642*	*0.1423*	*0.1728*	*0.0955*	*0.8089*
