# Supplementary material for: Role of the BAHD1 Chromatin-Repressive Complex in Placental Development and Regulation of Steroid Metabolism
Source: PLoS Genet. 2016 Mar 3;12(3):e1005898. doi: 10.1371/journal.pgen.1005898 (PMC4777444; doi:10.1371/journal.pgen.1005898)
Supplement: S3 Table — (PDF) [file pgen.1005898.s010.pdf]

Table S3: Genes differentially expressed in *Bahd1*-KO placentas compared to *Bahd1*-WT placentas at both E16.5 and E18.5 stages

**Up-regulated genes**	
**GENE_SYMBOL**	**Name**
A2M	alpha-2-macroglobulin
ABP1	amiloride binding protein 1 (amine oxidase, copper-containing)
ACOX3	acyl-Coenzyme A oxidase 3, pristanoyl
ADAMTS2	a disintegrin-like and metallopeptidase (reprolysin type) with thrombospondin type 1 motif, 2
ADAMTS5	similar to a disintegrin-like and metalloprotease (reprolysin type) with thrombospondin type 1 motif, 5 (aggrecanase-2)
ADM	adrenomedullin
AES	amino-terminal enhancer of split
AIF1L	allograft inflammatory factor 1-like
AMPD3	adenosine monophosphate deaminase 3
ANGPTL2	angiopoietin-like 2
ANO1	anoctamin 1, calcium activated chloride channel
ANTXR1	anthrax toxin receptor 1
ANXA8	annexin A8
ANXA9	annexin A9
APOC3	apolipoprotein C-III
AQP1	aquaporin 1
ARL4D	ADP-ribosylation factor-like 4D; hypothetical protein LOC100044157
ASRGL1	asparaginase like 1
ATP6V0D2	ATPase, H+ transporting, lysosomal V0 subunit D2
ATP8B1	ATPase, class I, type 8B, member 1
B3GALNT1	UDP-GalNAc:betaGlcNAc beta 1,3-galactosaminyltransferase, polypeptide 1
BMP8A	bone morphogenetic protein 8a
BNIP3	BCL2/adenovirus E1B interacting protein 3
BTBD3	BTB (POZ) domain containing 3
C1QA	complement component 1, q subcomponent, alpha polypeptide
CCND2	cyclin D2
CD109	CD109 antigen
CD55	CD55 antigen
CDH13	cadherin 13
CDKN1A	cyclin-dependent kinase inhibitor 1A (P21)
CDO1	cysteine dioxygenase 1, cytosolic
CFLAR	CASP8 and FADD-like apoptosis regulator pseudogene; CASP8 and FADD-like apoptosis regulator
CHST11	carbohydrate sulfotransferase 11
CHST12	carbohydrate sulfotransferase 12
CLDN1	claudin 1
CLDN5	claudin 5
CLEC2D	C-type lectin domain family 2, member d
COCH	coagulation factor C homolog (Limulus polyphemus)
CPE	carboxypeptidase E; similar to carboxypeptidase E
CRABP1	cellular retinoic acid binding protein I
CRABP2	cellular retinoic acid binding protein II
CTSK	cathepsin K
CTSO	cathepsin O
CTSS	cathepsin S
CXCL14	chemokine (C-X-C motif) ligand 14
CYP11A1	cytochrome P450, family 11, subfamily a, polypeptide 1
DCAF4	WD repeat domain 21
DEGS2	degenerative spermatocyte homolog 2 (Drosophila), lipid desaturase
DNAJC18	DnaJ (Hsp40) homolog, subfamily C, member 18
DTNA	dystrobrevin alpha
ECHDC2	enoyl Coenzyme A hydratase domain containing 2
EGLN3	EGL nine homolog 3 (C. elegans)
EMCN	endomucin
EMILIN2	elastin microfibril interfacer 2
EPDR1	ependymin related protein 1 (zebrafish)
ERV3	endogenous retroviral sequence 3
ESR1	estrogen receptor 1 (alpha)
FABP4	fatty acid binding protein 4, adipocyte
FAM135B	family with sequence similarity 135, member B
FAM174B	family with sequence similarity 174, member B
FAM20C	family with sequence similarity 20, member C
FBLN2	fibulin 2
FCER1G	Fc receptor, IgE, high affinity I, gamma polypeptide
FMO2	flavin containing monooxygenase 2
FSTL1	follistatin-like 1
FZD1	frizzled homolog 1 (Drosophila)
GADD45G	growth arrest and DNA-damage-inducible 45 gamma
GALC	galactosylceramidase
GATM	glycine amidinotransferase (L-arginine:glycine amidinotransferase)
GDPD1	glycerophosphodiester phosphodiesterase domain containing 1
GGTA1	glycoprotein galactosyltransferase alpha 1, 3
GJA1	gap junction protein, alpha 1
GKN2	gastrokine 2
GLIPR1	GLI pathogenesis-related 1 (glioma)
GNG11	guanine nucleotide binding protein (G protein), gamma 11
GPIHBP1	GPI-anchored HDL-binding protein 1
GPNMB	glycoprotein (transmembrane) nmb
GPX8	glutathione peroxidase 8 (putative)
GRAMD1B	GRAM domain containing 1B
GRAP	GRB2-related adaptor protein
GSDMD	gasdermin D
GZMC	granzyme C
GZMD	granzyme D
GZMG	granzyme G
H2-Q6	histoompatibility 2, Q region locus 6
H2-T22	histocompatibility 2, T region locus 22
HAND2	heart and neural crest derivatives expressed transcript 2
HBA-X	hemoglobin X, alpha-like embryonic chain in Hba complex
HBB-Y	similar to beta-globin; hemoglobin Y, beta-like embryonic chain
HOXA11	homeo box A11

HOXD10	homeo box D10
HPGD	hydroxyprostaglandin dehydrogenase 15 (NAD)
HPSE	heparanase
HSPB7	heat shock protein family, member 7 (cardiovascular)
HSPB8	heat shock protein 8
HTRA1	HtrA serine peptidase 1
HTRA3	HtrA serine peptidase 3
IFITM3	interferon induced transmembrane protein 3
IGFBP3	insulin-like growth factor binding protein 3
INMT	indolethylamine N-methyltransferase
INPP5A	inositol polyphosphate-5-phosphatase A
INSIG2	insulin induced gene 2
JAM2	junction adhesion molecule 2
KLK15	kallikrein related-peptidase 15
LBP	lipopolysaccharide binding protein
LCN2	lipocalin 2
LY6G6C	lymphocyte antigen 6 complex, locus G6C
MASP1	mannan-binding lectin serine peptidase 1
MATN2	matrilin 2
MFAP5	microfibrillar associated protein 5
MGP	matrix Gla protein
MLPH	melanophilin
MMP11	matrix metallopeptidase 11
MMP14	matrix metallopeptidase 14 (membrane-inserted)
MMP2	matrix metallopeptidase 2
MMP23	matrix metallopeptidase 23
MRGPRG	MAS-related GPR, member G
MUSTN1	musculoskeletal, embryonic nuclear protein 1
MXRA8	matrix-remodelling associated 8
MYO5A	myosin VA
NAMPT	nicotinamide phosphoribosyltransferase
NBL1	neuroblastoma, suppression of tumorigenicity 1
NMB	neuromedin B
NPR2	natriuretic peptide receptor 2
NPY	neuropeptide Y
OSBPL5	oxysterol binding protein-like 5
P2RX4	purinergic receptor P2X, ligand-gated ion channel 4
PADI2	peptidyl arginine deiminase, type II; similar to peptidyl arginine deiminase, type II
PAM	peptidylglycine alpha-amidating monooxygenase
PAMR1	peptidase domain containing associated with muscle regeneration 1
PBX1	pre B-cell leukemia transcription factor 1
PDLIM3	PDZ and LIM domain 3
PGR	progesterone receptor
PHLDA3	pleckstrin homology-like domain, family A, member 3
PI15	peptidase inhibitor 15
PKNOX2	Pbx/knotted 1 homeobox 2
PLA1A	phospholipase A1 member A
PLEKHO1	pleckstrin homology domain containing, family O member 1
PLEKHO2	pleckstrin homology domain containing, family O member 2
PLXND1	plexin D1
PPP2R5A	similar to protein phosphatase 2, regulatory subunit B (B56)
PQLC3	PQ loop repeat containing
PRAP1	proline-rich acidic protein 1
PRL8A2	prolactin family 8, subfamily a, member 2
PRSS35	protease, serine, 35
PSMB9	proteasome (prosome, macropain) subunit, beta type 9 (large multifunctional peptidase 2)
PTGS1	prostaglandin-endoperoxide synthase 1
PTRH1	peptidyl-tRNA hydrolase 1 homolog (S. cerevisiae)
QPCT	glutaminyl-peptide cyclotransferase (glutaminyl cyclase)
RAB31	RAB31, member RAS oncogene family
RAB32	RAB32, member RAS oncogene family
RAMP1	receptor (calcitonin) activity modifying protein 1
RAMP3	receptor (calcitonin) activity modifying protein 3; similar to receptor activity modifying protein 3
RASGRP3	RAS, guanyl releasing protein 3
RGS2	regulator of G-protein signaling 2
RHO	rhodopsin
RHOJ	ras homolog gene family, member J
RIMKLB	ribosomal modification protein rimK-like family member B; similar to mKIAA1238 protein
RRM1	ribonucleotide reductase M1
RUNX1	runt related transcription factor 1
S100A4	hippocampus abundant transcript-like 1; S100 calcium binding protein A4
SCGB1A1	secretoglobin, family 1A, member 1 (uteroglobin)
SCMH1	sex comb on midleg homolog 1
SERPING1	serine (or cysteine) peptidase inhibitor, clade G, member 1
SETD7	SET domain containing (lysine methyltransferase) 7
SEZ6L	seizure related 6 homolog like
SFRP4	secreted frizzled-related protein 4
SFRP5	secreted frizzled-related sequence protein 5
SGK1	serum/glucocorticoid regulated kinase 1
SGPL1	sphingosine phosphate lyase 1
SIRPA	signal-regulatory protein alpha
SLC25A29	solute carrier family 25 (mitochondrial carrier, palmitoylcarnitine transporter), member 29
SLC44A1	solute carrier family 44, member 1
SLC6A12	solute carrier family 6 (neurotransmitter transporter, betaine/GABA), member 12
SLCO5A1	solute carrier organic anion transporter family, member 5A1
SLPI	secretory leukocyte peptidase inhibitor
SMOC2	SPARC related modular calcium binding 2
SORBS2	sorbin and SH3 domain containing 2
SPHK1	sphingosine kinase 1
SPON1	spondin 1, (f-spondin) extracellular matrix protein
SPP1	secreted phosphoprotein 1
SQRDL	sulfide quinone reductase-like (yeast)
SRGN	serglycin
SWAP70	SWA-70 protein
SYNE2	synaptic nuclear envelope 2
TACC1	transforming, acidic coiled-coil containing protein 1

TCP11L2	t-complex 11 (mouse) like 2
TDO2	tryptophan 2,3-dioxygenase
TFF3	trefoil factor 3, intestinal
TFPI2	tissue factor pathway inhibitor 2
THY1	thymus cell antigen 1, theta
TLR4	toll-like receptor 4
TMEM100	transmembrane protein 100
TMEM150A	transmembrane protein 150
TMEM50A	transmembrane protein 50A
TNFRSF11B	tumor necrosis factor receptor superfamily, member 11b (osteoprotegerin)
TNFRSF1B	tumor necrosis factor receptor superfamily, member 1b
TNFSF9	tumor necrosis factor (ligand) superfamily, member 9
TREM2	triggering receptor expressed on myeloid cells 2
TRP53INP1	transformation related protein 53 inducible nuclear protein 1
TSPAN5	tetraspanin 5
TXNIP	thioredoxin interacting protein
TYROBP	TYRO protein tyrosine kinase binding protein
UGCG	UDP-glucose ceramide glucosyltransferase
USP1	ubiquitin specific peptidase 1; predicted gene 5841
USP53	ubiquitin specific peptidase 53
VASN	vasorin
VEZF1	vascular endothelial zinc finger 1
VLDLR	very low density lipoprotein receptor
WEE1	WEE 1 homolog 1 (S. pombe)
WT1	similar to Wilms tumor homolog; Wilms tumor 1 homolog
XDH	xanthine dehydrogenase
XPA	xeroderma pigmentosum, complementation group A
ZFP36L1	zinc finger protein 36, C3H type-like 1

**Down-regulated genes GENE_SYMBOL**	**Name**
ADA	adenosine deaminase
ALDH1A3	aldehyde dehydrogenase family 1, subfamily A3
BAHD1	Bromo adjacent homology domain protein 1
BHLHE41	basic helix-loop-helix family, member e41
CAR2	carbonic anhydrase 2
CCR1L1	chemokine (C-C motif) receptor 1-like 1
ETS2	E26 avian leukemia oncogene 2, 3' domain
FABP6	fatty acid binding protein 6, ileal (gastrotropin)
FGG	fibrinogen gamma chain
FHL3	four and a half LIM domains 3
FSTL3	follistatin-like 3
GCM1	glial cells missing homolog 1 (Drosophila)
GHRH	growth hormone releasing hormone
GJB5	gap junction protein, beta 5
GPD2	glycerol phosphate dehydrogenase 2, mitochondrial
GPR1	G protein-coupled receptor 1
HGD	homogentisate 1, 2-dioxygenase
HSD17B2	hydroxysteroid (17-beta) dehydrogenase 2
HSD17B7	hydroxysteroid (17-beta) dehydrogenase 7
HSPA5	heat shock protein 5
LEPR	leptin receptor
LGALS9	lectin, galactose binding, soluble 9
MFSD2A	major facilitator superfamily domain containing 2
MUC1	mucin 1, transmembrane
NOSTRIN	nitric oxide synthase trafficker
NPPC	natriuretic peptide precursor type C
NUAK1	NUAK family, SNF1-like kinase, 1
PGA5	pepsinogen 5, group I
PITRM1	pitrilysin metallepetidase 1
PKLR	pyruvate kinase liver and red blood cell
PLAT	plasminogen activator, tissue
PRL3C1	prolactin family 3, subfamily c, member 1
PRL7A1	prolactin family 7, subfamily a, member 1
PRL7C1	prolactin family 7, subfamily c, member 1; predicted gene 2544
PSTPIP1	proline-serine-threonine phosphatase-interacting protein 1
PYGL	liver glycogen phosphorylase
SERPINB9C	serine (or cysteine) peptidase inhibitor, clade B, member 9c
SH3KBP1	SH3-domain kinase binding protein 1
ST6GAL1	beta galactoside alpha 2,6 sialyltransferase 1
STRA6	stimulated by retinoic acid gene 6
TFRC	transferrin receptor
TMEM54	transmembrane protein 54
TNFRSF9	tumor necrosis factor receptor superfamily, member 9
TUBB3	tubulin, beta 3; tubulin, beta 3, pseudogene 1
UBA6	ubiquitin-like modifier activating enzyme 6
UPP1	uridine phosphorylase 1
