# Supplementary material for: Role of the BAHD1 Chromatin-Repressive Complex in Placental Development and Regulation of Steroid Metabolism
Source: PLoS Genet. 2016 Mar 3;12(3):e1005898. doi: 10.1371/journal.pgen.1005898 (PMC4777444; doi:10.1371/journal.pgen.1005898)
Supplement: S4 Table — (PDF) [file pgen.1005898.s011.pdf]

Table S4. Biological processes associated with genes deregulated in *Bahd1*-KO murine placentae, *Bahd1*-KO MEFs and in human HEK-BAHD1 cells.
Analysis of clustering of differentially expressed genes into GO (Biological processes) with the DAVID program version 6.7 2015 (http://david.abcc.ncifcrf.gov)
The 10 most significant biological functions and associated group of genes are shown.
Genes involved in steroid/lipid metabolism are highlighted in yellow

A- Functional clustering of genes deregulated in *Bahd1*-KO murine placentas at E16.5

GO	Term	*P* Value	- Log10 (P value)	Count	Genes	FDR
GO:0008202	steroid metabolic process	1.47E-06	5.83	16	OSBPL5, HSD17B2, CYP11A1, LDLR, LEPR, HMGCS1, CYP17A1, HMGCS2, INSIG2, APOE, APOC3, ATP8B1, PBX1, HSD17B7, VLDLR, FABP6	2.49E-03
GO:0006979	response to oxidative stress	1.94E-06	5.71	12	TXNIP, XPA, GCLC, CYP11A1, GATM, APOE, PTGS1, GPX3, CLU, GPX8, MMP14, PRNP	3.29E-03
GO:0001501	skeletal system development	9.26E-06	5.03	20	SGPL1, HEXA, HOXA11, TCFAP2A, MGP, NPR2, MMP14, MMP2, HOXD10, AHSG, AES, INSIG2, CHST11, HOXA10, PBX1, RUNX1, GPNMB, IGFBP3, SPP1, BMP8A	1.57E-02
GO:0008203	cholesterol metabolic process	1.47E-05	4.83	10	LDLR, INSIG2, HMGCS2, CYP11A1, APOE, LEPR, APOC3, HMGCS1, HSD17B7, VLDLR	2.49E-02
GO:0001568	blood vessel development	1.56E-05	4.81	18	SGPL1, EMCN, CDX2, LEPR, SPHK1, GJA1, ANPEP, MMP14, MMP2, WT1, THY1, ZFP36L1, MYO18B, HAND2, APOE, LOX, VEZF1, PLXND1	2.64E-02
GO:0001944	vasculature development	2.13E-05	4.67	18	SGPL1, EMCN, CDX2, LEPR, SPHK1, GJA1, ANPEP, MMP14, MMP2, WT1, THY1, ZFP36L1, MYO18B, HAND2, APOE, LOX, VEZF1, PLXND1	3.62E-02
GO:0016125	sterol metabolic process	3.21E-05	4.49	10	LDLR, INSIG2, HMGCS2, CYP11A1, APOE, LEPR, APOC3, HMGCS1, HSD17B7, VLDLR	5.44E-02
GO:0019835	cytolysis	9.67E-05	4.01	6	LYZ2, GZMD, GZME, GZMC, GZMF, GZMG	1.64E-01
GO:0006508	proteolysis	1.19E-04	3.93	41	MASP1, PAMR1, UBA6, ANPEP, MMP23, MMP2, USP53, HTRA1, PITRM1, LONRF3, PGA5, HTRA3, PRSS35, KLK15, SRGN, PLAT, CFLAR, SPSB1, USP1, GZMD, GZME, SERPING1, GZMC, CTSS, MMP14, GZMF, MMP11, PSMB9, GZMG, QPCT, C1QA, CD55, CTSK, RNF7, CPE, CTSO, ZRANB1, PAPPA2, ADAMTS2, PMEPA1, ADAMTS5	2.01E-01
GO:0008219	cell death	1.75E-04	3.76	25	CLU, EGLN3, BNIP3, TNFRSF11B, TNFRSF1B, BOK, HAND2, TRP53INP1, PHLDA3, SRGN, CFLAR, SGPL1, SGK1, LYZ2, GZMD, FAM188A, GZME, GZMC, GZMF, GZMG, TNS4, RNF130, RNF7, GADD45G, SH3KBP1	2.97E-01

B- Functional clustering of genes deregulated in *Bahd1*-KO murine placentas at both E16.5 and E18.5 stages

GO	Term	*P* Value	- Log10 (P value)	Count	Genes	FDR
GO:0001501	skeletal system development	2.35E-05	4.63	16	SGPL1, HOXA11, MGP, NPR2, MMP14, MMP2, HOXD10, AES, INSIG2, CHST11, PBX1, RUNX1, GPNMB, IGFBP3, SPP1, BMP8A	3.96E-02
GO:0008202	steroid metabolic process	2.85E-05	4.55	11	OSBPL5, INSIG2, CYP11A1, HSD17B2, LEPR, APOC3, ATP8B1, PBX1, HSD17B7, FABP6, VLDLR	4.79E-02
GO:0051605	protein maturation by peptide bond cleavage	4.85E-05	4.31	8	C1QA, CD55, MASP1, CPE, SERPING1, MMP14, ADAMTS2, SRGN	8.15E-02
GO:0060348	bone development	6.56E-05	4.18	10	INSIG2, HOXA11, MGP, NPR2, GPNMB, MMP14, IGFBP3, MMP2, BMP8A, SPP1	1.10E-01
GO:0048584	positive regulation of response to stimulus	1.06E-04	3.98	12	C1QA, CD55, NPY, MASP1, FCER1G, FABP4, TLR4, SERPING1, LBP, H2-Q6, ADA, THY1	1.78E-01
GO:0006508	proteolysis	1.35E-04	3.87	32	MASP1, PAMR1, UBA6, MMP23, MMP2, USP53, HTRA1, PITRM1, HTRA3, PGA5, PRSS35, KLK15, SRGN, PLAT, CFLAR, USP1, GZMD, SERPING1, GZMC, CTSS, MMP14, MMP11, PSMB9, GZMG, QPCT, C1QA, CD55, CTSK, CPE, CTSO, ADAMTS2, ADAMTS5	2.26E-01
GO:0032101	regulation of response to external stimulus	1.48E-04	3.83	9	TNFRSF1B, NPY, FCER1G, FABP4, TLR4, SERPING1, LBP, SCGB1A1, ADA	2.48E-01
GO:0050727	regulation of inflammatory response	1.93E-04	3.71	7	TNFRSF1B, FCER1G, FABP4, SERPING1, LBP, SCGB1A1, ADA	3.25E-01
GO:0050778	positive regulation of immune response	1.95E-04	3.71	10	C1QA, CD55, MASP1, FCER1G, TLR4, SERPING1, LBP, H2-Q6, ADA, THY1	3.28E-01
GO:0001568	blood vessel development	2.91E-04	3.54	13	SGPL1, EMCN, LEPR, SPHK1, GJA1, MMP14, MMP2, WT1, THY1, ZFP36L1, HAND2, PLXND1, VEZF1	4.88E-01

C- Functional clustering of genes up-regulated in Bahd1-KO mouse embryonic fibroblasts

GO	Term	*P* Value	- Log10 (P value)	Count	Genes	FDR
GO:0016125	sterol metabolic process	3.65E-09	8.44	14	SOAT1, SC5D, LDLR, MVD, HMGCR, CH25H, PCTP, HMGCS1, PMVK, HSD17B7, DHCR24, SREBF2, SC4MOL, NSDHL	6.23E-06
GO:0016126	sterol biosynthetic process	4.48E-09	8.35	10	SC5D, MVD, HMGCR, CH25H, HMGCS1, PMVK, HSD17B7, SC4MOL, NSDHL, DHCR24	7.64E-06
GO:0001763	morphogenesis of a branching structure	2.86E-08	7.54	16	BMP4, IL6, FGF7, NRP1, FLT1, PLXNA1, CSF1, NPNT, EDN1, SRF, CCL11, NOTCH1, SFRP1, ADM, BCL2, AREG	4.87E-05
GO:0042325	regulation of phosphorylation	8.87E-08	7.05	23	BMP4, BCL10, CAV1, IL6, LYN, PDGFA, HMGCR, CSF1, EDN1, MET, TNFSF15, HGF, LATS2, SPRY4, TRIB1, LIF, PRKAR2B, PRKAR2A, EREG, BCL2, SPRED3, PPP1R14A, HTR2A	1.51E-04
GO:0008203	cholesterol metabolic process	1.28E-07	6.89	12	SOAT1, LDLR, MVD, HMGCR, CH25H, PCTP, HMGCS1, PMVK, HSD17B7, SREBF2, NSDHL, DHCR24	2.19E-04
GO:0051174	regulation of phosphorus metabolic process	1.70E-07	6.77	23	BMP4, BCL10, CAV1, IL6, LYN, PDGFA, HMGCR, CSF1, EDN1, MET, TNFSF15, HGF, LATS2, SPRY4, TRIB1, LIF, PRKAR2B, PRKAR2A, EREG, BCL2, SPRED3, PPP1R14A, HTR2A	2.91E-04
GO:0019220	regulation of phosphate metabolic process	1.70E-07	6.77	23	BMP4, BCL10, CAV1, IL6, LYN, PDGFA, HMGCR, CSF1, EDN1, MET, TNFSF15, HGF, LATS2, SPRY4, TRIB1, LIF, PRKAR2B, PRKAR2A, EREG, BCL2, SPRED3, PPP1R14A, HTR2A	2.91E-04
GO:0022612	gland morphogenesis	8.61E-07	6.07	12	CCL11, BMP4, NOTCH1, IL6, CAV1, FGF7, NRP1, PLXNA1, SFRP1, BCL2, CSF1, AREG	1.47E-03
GO:0008284	positive regulation of cell proliferation	1.13E-06	5.95	21	KLF5, IL6, FGF7, CCL2, LYN, PDGFA, CSF1, CLU, EDN1, BEX1, LIFR, LIF, CD38, NOTCH1, EREG, ADM, BCL2, HIPK2, HBEGF, MYC, HTR2A	1.93E-03
GO:0006694	steroid biosynthetic process	1.38E-06	5.86	11	SC5D, MVD, HMGCR, CH25H, HMGCS1, LSS, PMVK, HSD17B7, SC4MOL, NSDHL, DHCR24	2.35E-03

D- Functional clustering of genes down-regulated in human cells overexpressing BAHD1 (HEK-BAHD1)

GO	Term	*P* Value	- Log10 (P value)	Count	Genes	FDR
GO:0055114	oxidation reduction	1.57E-08	7.81	74	STEAP3, SEPX1, CYP24A1, PXDN, LDHA, CYP2J2, CYP2S1, PYROXD1, SNCA, PRDX4, ALDH1L2, HIBADH, GLDC, SC4MOL, FDFT1, PLOD3, AKR7A2, SRD5A3, LOX, DHTKD1, SARDH, DUS1L, LOXL1, NQO2, DHCR24, CTBP1, PCYOX1L, ACOXL, MICAL2, CYB5A, CBR4, MOSC1, GRHPR, DECR1, GMPR, MOXD1, PYCR1, PYCR2, SQLE, ALDH1B1, ASPHD2, HSD11B2, MECR, MDH2, ME1, ACADSB, SORD, ALDH18A1, CYP51A1, GLUD1, IFI30, RSAD1, GPD1L, ALDH1A2, ALDH1A3, DHCR7, IDH2, FASN, DHODH, PNPO, HSD17B4, NSDHL, CHDH, PTGR1, SCD, MAOB, BCKDHB, FADS2, MSRB2, BLVRA, CYBA, AKR1B1, ALDH2, PHGDH	2.84E-05
GO:0016125	sterol metabolic process	2.54E-07	6.60	22	SREBF1, EBP, LIPA, LDLR, CYP51A1, RXRA, HMGCS1, ABCA2, LDLRAP1, SREBF2, SC4MOL, FDFT1, SQLE, APOE, DHCR7, INSIG1, SCARB1, IDI1, MBTPS1, DHCR24, NSDHL, CLN6	4.61E-04
GO:0008203	cholesterol metabolic process	1.05E-06	5.98	20	SREBF1, EBP, LDLR, CYP51A1, RXRA, HMGCS1, ABCA2, LDLRAP1, SREBF2, FDFT1, SQLE, APOE, DHCR7, INSIG1, SCARB1, IDI1, MBTPS1, DHCR24, NSDHL, CLN6	1.90E-03
GO:0006631	fatty acid metabolic process	1.20E-06	5.92	31	ACADSB, CPT2, CYP2J2, SNCA, PEX5, MIF, SC4MOL, PRKAR2B, ACSL1, ELOVL5, ELOVL2, FASN, GNPAT, ELOVL7, HSD17B4, ACSL4, LPL, PTGR1, LIPA, ACOXL, PDPN, SCD, EPHX2, FADS2, LYPLA2, DECR1, PECI, CPT1A, ACSM3, LTA4H, MECR	2.18E-03
GO:0008610	lipid biosynthetic process	4.46E-06	5.35	41	CYP51A1, HMGCS1, MIF, FDFT1, SC4MOL, ALDH1A2, B3GNT5, ELOVL5, DHCR7, PIGH, ELOVL2, PEMT, FASN, ELOVL7, SCARB1, ETNK2, PCYT2, AGPAT2, DHCR24, NSDHL, B4GALNT1, LPL, EBP, SCD, FADS2, ACLY, CDS1, LPCAT2, LPCAT3, PIGO, LPCAT4, ACSM3, LASS4, SQLE, CD81, MBOAT2, HSD11B2, DPM3, LTA4H, IDI1, MECR	8.09E-03
GO:0031344	regulation of cell projection organization	1.16E-05	4.93	18	LZTS1, PTPRF, MAP1B, ITGA2, RHOQ, CDH4, SLIT2, EPHB2, NRCAM, PRKCQ, LINGO1, SEMA4F, APOE, ROBO2, NEFL, DBN1, ARHGDIA, NEFM	2.11E-02
GO:0044271	nitrogen compound biosynthetic process	1.24E-05	4.91	40	ATP5D, MOCOS, ME1, BCAT1, ADCY1, ALDH18A1, SRM, ADCY7, ASS1, GLUD1, SNCA, UROS, RSAD1, ATP5G1, TGFB1, ADA, GOT2, AKT1, SRR, ATP8B1, DHODH, CDA, SULT1A2, ETNK2, PRTFDC1, PCBD1, PADI2, CPS1, ATP13A2, MMAB, APRT, NME4, PYCR1, ATP6V1C2, PYCR2, GLUL, ATP9A, PHGDH, SLC25A15, PRPS2	2.24E-02
GO:0019318	hexose metabolic process	1.66E-05	4.78	28	PHKA2, LDHA, SORD, PFKFB4, PFKFB3, SLC37A4, HK2, HIBADH, AKT1, PPP1R1A, DHTKD1, MYC, PDK1, PDK2, GMDS, PDK3, PFKP, GYG1, PFKM, CPS1, CPT1A, GPI, SLC25A10, GFPT2, PGM1, GAA, POFUT1, MDH2	3.02E-02
GO:0010033	response to organic substance	2.37E-05	4.62	70	ADCY1, LDLR, ADCY7, SNCA, RHOQ, TGFB1, GOT2, AKT1, PRKAR2B, CCNE1, GSTM3, AES, CD44, GSN, APOE, LOX, GNG4, MYC, EGFR, KCNMA1, RXRA, MFGE8, PTPRU, LPIN1, CDK5, GNAL, PRKCQ, GLUL, KRT19, CCND1, SDC1, GRB10, CCND2, SQLE, HSPB1, HSD11B2, ABAT, RYR2, CTSC, GNB4, CA2, LY6G6D, ME1, ENPP1, HMGCS1, UROS, ABCA2, CDH1, COMT, GNG12, AK3L1, TRIB1, ALDH1A2, ACSL1, TAP2, PEMT, SRR, SCARB1, NEFL, MAP1B, BCKDHB, ITGA2, CPS1, SREBF2, RERG, CXCL16, PLCG2, ALDH2, IGFBP2, BMP7	4.31E-02
GO:0016053	organic acid biosynthetic process	2.92E-05	4.53	24	BCAT1, LPL, ALDH18A1, ASS1, GLUD1, SCD, UROS, FADS2, MIF, SC4MOL, ACSM3, GOT2, PYCR1, PYCR2, GLUL, ELOVL5, ELOVL2, SRR, FASN, PHGDH, LTA4H, ELOVL7, MECR, AGPAT2	5.30E-02

E- Functional clustering of genes down-regulated in human cells overexpressing BAHD1 (HEK-BAHD1) and up-regulated in *Bahd1*-KO placentas at E18.5

GO	Term	*P* Value	- Log10 (P value)	Count	Genes	FDR
GO:0010817	regulation of hormone levels	2.85E-04	3.54	7	SLC16A2, ALDH1A2, LY6E, BACE2, CAMK2G, CRABP2, SCARB1	4.57E-01
GO:0008610	lipid biosynthetic process	6.85E-04	3.16	9	LPL, ALDH1A2, EBP, LASS4, CD81, ELOVL7, SCARB1, LTA4H, LPCAT2	1.09E+00
GO:0001655	urogenital system development	4.26E-03	2.37	5	SGPL1, ALDH1A2, SFRP1, HOXA11, ADAMTS1	6.62E+00
GO:0048705	skeletal system morphogenesis	4.54E-03	2.34	5	SGPL1, HOXA11, PRRX1, MMP2, TGFB1	7.05E+00
GO:0006631	fatty acid metabolic process	6.66E-03	2.18	6	LPL, PRKAR2B, PTGR1, ELOVL7, LTA4H, CPT1A	1.02E+01
GO:0006643	membrane lipid metabolic process	1.26E-02	1.90	4	SGPL1, ST6GALNAC4, LASS4, CERK	1.84E+01
GO:0035113	embryonic appendage morphogenesis	1.53E-02	1.82	4	ALDH1A2, HOXA11, CRABP2, PRRX1	2.19E+01
GO:0030326	embryonic limb morphogenesis	1.53E-02	1.82	4	ALDH1A2, HOXA11, CRABP2, PRRX1	2.19E+01
GO:0040008	regulation of growth	1.64E-02	1.79	7	NOV, SGPL1, HTRA2, HTRA1, CXCL16, GAS6, TGFB1	2.33E+01
GO:0001822	kidney development	1.98E-02	1.70	4	SGPL1, ALDH1A2, HOXA11, ADAMTS1	2.75E+01

F- Functional clustering of genes down-regulated in human cells overexpressing BAHD1 (HEK-BAHD1) and up-regulated in *Bahd1*-KO MEFs

GO	Term	*P* Value	- Log10 (P value)	Count	Genes	FDR
GO:0016125	sterol metabolic process	5.71E-06	5.24	6	LDLR, HMGCS1, DHCR24, SREBF2, SC4MOL, NSDHL	8.39E-03
GO:0008203	cholesterol metabolic process	8.93E-05	4.05	5	LDLR, HMGCS1, DHCR24, SREBF2, NSDHL	1.31E-01
GO:0016126	sterol biosynthetic process	9.81E-05	4.01	4	HMGCS1, DHCR24, SC4MOL, NSDHL	1.44E-01
GO:0008202	steroid metabolic process	1.59E-04	3.80	6	LDLR, HMGCS1, DHCR24, SREBF2, SC4MOL, NSDHL	2.34E-01
GO:0006694	steroid biosynthetic process	1.36E-03	2.87	4	HMGCS1, DHCR24, SC4MOL, NSDHL	1.97E+00
GO:0006695	cholesterol biosynthetic process	2.03E-03	2.69	3	HMGCS1, DHCR24, NSDHL	2.95E+00
GO:0055114	oxidation reduction	5.46E-03	2.26	7	PCYOX1L, MICAL2, ALDH1A3, ASPHD2, DHCR24, SC4MOL, NSDHL	7.73E+00
GO:0010033	response to organic substance	9.69E-03	2.01	7	PRKAR2B, KRT19, LDLR, HMGCS1, MYC, TRIB1, SREBF2	1.33E+01
GO:0019318	hexose metabolic process	1.32E-02	1.88	4	PFKFB3, GFPT2, HK2, MYC	1.77E+01
GO:0005996	monosaccharide metabolic process	1.94E-02	1.71	4	PFKFB3, GFPT2, HK2, MYC	2.50E+01
GO:0009719	response to endogenous stimulus	1.99E-02	1.70	5	PRKAR2B, KRT19, LDLR, HMGCS1, SREBF2	2.56E+01
GO:0051146	striated muscle cell differentiation	2.17E-02	1.66	3	TNNT2, KRT19, ADAM12	2.75E+01
