# Supplementary material for: Role of the BAHD1 Chromatin-Repressive Complex in Placental Development and Regulation of Steroid Metabolism
Source: PLoS Genet. 2016 Mar 3;12(3):e1005898. doi: 10.1371/journal.pgen.1005898 (PMC4777444; doi:10.1371/journal.pgen.1005898)
Supplement: S5 Table — (PDF) [file pgen.1005898.s012.pdf]

Table S5. Overlap between transcriptome datasets from *Bahd1*-KO MEFs, *Bahd1*-KO placentas (E18.5) and human HEK-BAHD1 cells.

GENE_SYMBOL	Name
**A- List of genes up-regulated in murine *Bahd1*-KO placentas (E18.5) and down-regulated in human HEK-BAHD1 cells**	
ABCA2	ATP-binding cassette, sub-family A (ABC1), member 2
ABR	active BCR-related gene
ACSS1	acyl-CoA synthetase short-chain family member 1
ADAM12	ADAM metallopeptidase domain 12
ADAMTS1	ADAM metallopeptidase with thrombospondin type 1 motif, 1
ADD3	adducin 3 (gamma)
AES	amino-terminal enhancer of split
ALDH1A2	aldehyde dehydrogenase 1 family, member A2
ALDH2	aldehyde dehydrogenase 2 family (mitochondrial)
ATHL1	ATH1, acid trehalase-like 1 (yeast)
ATP8B1	ATPase, class I, type 8B, member 1
BACE2	beta-site APP-cleaving enzyme 2
BNIP3	BCL2/adenovirus E1B 19kDa interacting protein 3
BRE	brain and reproductive organ-expressed (TNFRSF1A modulator)
CAMK2G	calcium/calmodulin-dependent protein kinase II gamma
CASK	calcium/calmodulin-dependent serine protein kinase (MAGUK family)
CCDC3	coiled-coil domain containing 3
CCND2	cyclin D2
CD81	CD81 molecule
CD99L2	CD99 molecule-like 2
CERK	ceramide kinase
CHCHD10	coiled-coil-helix-coiled-coil-helix domain containing 10
CHST12	carbohydrate (chondroitin 4) sulfotransferase 12
CPT1A	carnitine palmitoyltransferase 1A (liver)
CRABP1	cellular retinoic acid binding protein 1
CRABP2	cellular retinoic acid binding protein 2
CXCL16	chemokine (C-X-C motif) ligand 16
DBNDD2	dysbindin (dystrobrevin binding protein 1) domain containing 2
DPYSL2	dihydropyrimidinase-like 2
EBP	emopamil binding protein (sterol isomerase)
EFHD1	EF-hand domain family, member D1
EHD3	EH-domain containing 3
ELOVL7	ELOVL family member 7, elongation of long chain fatty acids (yeast)
EMP2	epithelial membrane protein 2
ENPP2	ectonucleotide pyrophosphatase/phosphodiesterase 2
FBLN5	fibulin 5
GALNT11	UDP-N-acetyl-alpha-D-galactosamine:polypeptide N-acetylgalactosaminyltransferase 11 (GalNAc-T11)
GAS6	similar to growth arrest-specific 6; growth arrest-specific 6
GEM	GTP binding protein overexpressed in skeletal muscle
GLDC	glycine dehydrogenase (decarboxylating)
GLTP	glycolipid transfer protein; glycolipid transfer protein pseudogene 1
GMPR	guanosine monophosphate reductase
GPRC5B	G protein-coupled receptor, family C, group 5, member B
GPT2	glutamic pyruvate transaminase (alanine aminotransferase) 2
GSTT2	glutathione S-transferase theta 2B (gene/pseudogene); glutathione S-transferase theta 2
HOXA11	homeobox A11
HSPB8	heat shock 22kDa protein 8
HTRA1	HtrA serine peptidase 1
HTRA2	HtrA serine peptidase 2
IL17RD	interleukin 17 receptor D
ITGB5	integrin, beta 5
KIRREL	kin of IRRE like (Drosophila)
LASS4	LAG1 homolog, ceramide synthase 4
LPCAT2	lysophosphatidylcholine acyltransferase 2
LPL	lipoprotein lipase
LTA4H	leukotriene A4 hydrolase
LY6E	lymphocyte antigen 6 complex, locus E
MAL2	mal, T-cell differentiation protein 2
MCAM	melanoma cell adhesion molecule
MMP2	matrix metallopeptidase 2 (gelatinase A, 72kDa gelatinase, 72kDa type IV collagenase)
MSC	musculin (activated B-cell factor-1)
MYO1D	myosin ID
NBL1	neuroblastoma, suppression of tumorigenicity 1
NDRG2	NDRG family member 2
NFIX	nuclear factor I/X (CCAAT-binding transcription factor)
NOV	nephroblastoma overexpressed gene
NRGN	neurogranin (protein kinase C substrate, RC3)
OTUB2	OTU domain, ubiquitin aldehyde binding 2
PADI2	peptidyl arginine deiminase, type II
PLLP	plasma membrane proteolipid (plasmolipin)
PRKAR2B	protein kinase, cAMP-dependent, regulatory, type II, beta
PRPS2	phosphoribosyl pyrophosphate synthetase 2
PRRX1	paired related homeobox 1
PRSS12	protease, serine, 12 (neurotrypsin, motopsin)
PTGR1	prostaglandin reductase 1
RASA3	RAS p21 protein activator 3
RGS19	regulator of G-protein signaling 19
SCARB1	scavenger receptor class B, member 1

SCMH1	sex comb on midleg homolog 1 (Drosophila)
SERPING1	serpin peptidase inhibitor, clade G (C1 inhibitor), member 1
SFRP1	secreted frizzled-related protein 1
SGPL1	sphingosine-1-phosphate lyase 1
SLC16A2	solute carrier family 16, member 2 (monocarboxylic acid transporter 8)
SLC44A1	solute carrier family 44, member 1
SLC6A6	solute carrier family 6 (neurotransmitter transporter, taurine), member 6
SOX4	SRY (sex determining region Y)-box 4
SPON1	spondin 1, extracellular matrix protein
ST6GALNAC4	ST6 (alpha-N-acetyl-neuraminyl-2,3-beta-galactosyl-1,3)-N-acetylgalactosaminide alpha-2,6-sialyltransferase 4
STEAP3	STEAP family member 3
SULF2	sulfatase 2
TC2N	tandem C2 domains, nuclear
TCP11L2	t-complex 11 (mouse)-like 2
TGFB1	transforming growth factor, beta 1
TIMP1	TIMP metallopeptidase inhibitor 1
TMEM109	transmembrane protein 109
TMEM144	transmembrane protein 144
TMEM159	transmembrane protein 159
TMEM98	similar to transmembrane protein 98; transmembrane protein 98
TMOD1	tropomodulin 1
TRIP10	thyroid hormone receptor interactor 10
TSHZ2	teashirt zinc finger homeobox 2
TSHZ3	teashirt zinc finger homeobox 3
TSPAN15	tetraspanin 15
TSPAN2	tetraspanin 2
UBE2E2	ubiquitin-conjugating enzyme E2E 2 (UBC4/5 homolog, yeast)
UCK2	uridine-cytidine kinase 2
UST	uronyl-2-sulfotransferase
**B-List of genes up-regulated in murine *Bahd1*-KO MEFs and down-regulated in human HEK-BAHD1 cells**	
ADAM12	ADAM metallopeptidase domain 12
ADORA2B	hypothetical LOC100131909; adenosine A2b receptor
ALDH1A3	aldehyde dehydrogenase 1 family, member A3
ASPHD2	aspartate beta-hydroxylase domain containing 2
COTL1	coactosin-like 1 (Dictyostelium)
DHCR24	24-dehydrocholesterol reductase
FAM129B	family with sequence similarity 129, member B
FLOT1	flotillin 1
GALNTL4	UDP-N-acetyl-alpha-D-galactosamine:polypeptide N-acetylgalactosaminyltransferase-like 4
GAS6	similar to growth arrest-specific 6; growth arrest-specific 6
GFPT2	glutamine-fructose-6-phosphate transaminase 2
GJB2	gap junction protein, beta 2, 26kDa
HK2	hexokinase 2 pseudogene; hexokinase 2
HMGCS1	3-hydroxy-3-methylglutaryl-Coenzyme A synthase 1 (soluble)
HPCAL1	hippocalcin-like 1
HSPA12A	heat shock 70kDa protein 12A
ITGA5	integrin, alpha 5 (fibronectin receptor, alpha polypeptide)
ITGA8	integrin, alpha 8
KRT19	keratin 19
LDLR	low density lipoprotein receptor
MCAM	melanoma cell adhesion molecule
MICAL2	microtubule associated monoxygenase, calponin and LIM domain containing 2
MYC	v-myc myelocytomatosis viral oncogene homolog (avian)
NSDHL	NAD(P) dependent steroid dehydrogenase-like
PCOLCE2	procollagen C-endopeptidase enhancer 2
PCYOX1L	prenylcysteine oxidase 1 like
PDE8B	phosphodiesterase 8B
PDLIM1	PDZ and LIM domain 1
PFKFB3	6-phosphofructo-2-kinase/fructose-2,6-biphosphatase 3
PRKAR2B	protein kinase, cAMP-dependent, regulatory, type II, beta
PTER	phosphotriesterase related
RGS16	regulator of G-protein signaling 16
SC4MOL	sterol-C4-methyl oxidase-like
SERINC2	serine incorporator 2
SFRP1	secreted frizzled-related protein 1
SLC2A1	solute carrier family 2 (facilitated glucose transporter), member 1
SREBF2	sterol regulatory element binding transcription factor 2
SYT17	synaptotagmin XVII; synaptotagmin VII
TBC1D1	TBC1 (tre-2/USP6, BUB2, cdc16) domain family, member 1
TNNT2	troponin T type 2 (cardiac)
TRIB1	tribbles homolog 1 (Drosophila)
TSPAN12	tetraspanin 12
TSPAN2	tetraspanin 2
TSPAN7	tetraspanin 7
**C- List of genes up-regulated in murine *Bahd1*-KO MEFs and placentas (E18.5) and down-regulated in human HEK-BAHD1 cells**	
ADAM12	a disintegrin and metallopeptidase domain 12 (meltrin alpha)
GAS6	growth arrest specific 6
MCAM	melanoma cell adhesion molecule
PRKAR2B	protein kinase, cAMP dependent regulatory, type II beta
SFRP1	secreted frizzled-related protein 1
TSPAN2	tetraspanin 2
