# Supplementary material for: Role of the BAHD1 Chromatin-Repressive Complex in Placental Development and Regulation of Steroid Metabolism
Source: PLoS Genet. 2016 Mar 3;12(3):e1005898. doi: 10.1371/journal.pgen.1005898 (PMC4777444; doi:10.1371/journal.pgen.1005898)
Supplement: S6 Table — (PDF) [file pgen.1005898.s013.pdf]

Table S6. Tandem affinity purification of BAHD1-associated partners.

Number of unique peptides for a protein identified by mass spectrometry in independent TAP assays of ProteinC-His6-tagged BAHD1 (HPT-BAHD1) in human HEK293 cells.
The same procedure was performed with control cells expressing the empty vector ("Control")

Dark pink: above 4 peptides
Pink: 1 to 4 peptides

			Tandem affinity purification and mass spectrometry										
			**Elution 1 (PC)**			**Elution2 (NI++)**				**Elution 1 (PC)**		**Elution 2 (NI++)**	
			Purification 2011 **(a)**	This work	This work	Purification 2011 **(a)**	This work	This work		This work	This work	This work	This work
**UniProt_Accession**	**UniProt Protein Name**	**Gene Symbol**	**HPT-BAHD1**	**HPT-BAHD1**	**HPT-BAHD1**	**HPT-BAHD1**	**HPT-BAHD1**	**HPT-BAHD1**	**TOP HIT (b)**	**HPT-Control**	**HPT-Control**	**HPT-Control**	**HPT-Control**
Q8TBE0	Bromo adjacent homology domain-containing 1	**BAHD1**	**22**	**41**	**33**	**26**	**39**	**22**	**BAHD1**				
Q8N108	Mesoderm induction early response protein 1	**MIER1**	**124**	**26**	**15**	**16**	**22**	**7**	**MIER1**				
Q7Z3K6	Mesoderm induction early response protein 3	**MIER3**	**2**	**13**	**7**	**7**	**8**	**3**	**MIER3**				
Q8N344	Mesoderm induction early response protein 2	**MIER2**		**1**	**1**		**1**	**1**	**MIER2**				
Q92769	Histone deacetylase 2	**HDAC2**		**11**	**7**	**13**	**10**	**2**	**HDAC2**				
Q13547	Histone deacetylase 1	**HDAC1**		**12**	**11**	**6**	**9**	**4**	**HDAC1**		**1**		
Q13185	Chromobox protein homolog 3	**HP1γ (c)**	**10**	**8**	**10**	**19**	**8**	**5**	**HP1γ**		**2**		
P83916	Chromobox protein homolog 1	**HP1β (c)**	**22**	**10**	**7**		**8**	**2**	**HP1β**				
P45973	Chromobox protein homolog 5	**HP1α (c)**	**17**	**7**	**5**		**3**				**2**		
Q9Y232	Chromodomain Y-like protein	**CDYL1**	**50**	**15**	**3**	**2**	**3**		**CDYL1**				
Q8N8U2	Chromodomain Y-like protein 2	**CDYL2**	**6**	**1**	**2**								
Q13263	Transcription intermediary factor 1-beta	**KAP1/TRIM28**		**12**	**6**	**1**	**4**	**2**	**TRIM28/KAP1**	**3**	**1**		
P30153	Serine/threonine-protein phosphatase 2A 65 kDa regulatory subunit A alpha isoform	**PPP2R1A**		**9**	**5**		**1**	**1**	**PPP2R1A**				
Q9Y230	RuvB-like 2	**RUVBL2**		**7**	**5**	**2**		**2**	**RUVBL2**				
Q9Y265	RuvB-like 1	**RUVBL1**		**4**	**6**			**2**					
Q92841	Probable ATP-dependent RNA helicase DDX17	**DDX17**	**4**	**1**	**7**	**8**		**1**	**DDX17**		**2**		
Q9NR30	Nucleolar RNA helicase 2 (Gu)	**DDX21/Gu (c)**	**4**	**11**	**13**	**3**				**6**			
Q96KQ7	Histone-lysine N-methyltransferase EHMT2	**G9a/EHMT2**	**48**	**3**									
Q12873	Chromodomain-helicase-DNA-binding protein 3	**CHD3**	**49**	**2**									
Q9UIS9	Methyl-CpG-binding domain protein 1	**MBD1 (c)**	**11**										

(a) Purification was performed in a previous study (Lebreton et al., 2011).
(b) Proteins that were pulled-down at least twice in the Elution 2 and absent in the control.
(c) Also found in a yeast-two hybrid screen of BAHD1 using a human placenta cDNA library (Bierne et al., 2009)
