# Supplementary material for: Role of the BAHD1 Chromatin-Repressive Complex in Placental Development and Regulation of Steroid Metabolism
Source: PLoS Genet. 2016 Mar 3;12(3):e1005898. doi: 10.1371/journal.pgen.1005898 (PMC4777444; doi:10.1371/journal.pgen.1005898)
Supplement: S7 Table — (PDF) [file pgen.1005898.s014.pdf]

Table S7. Transcription factors predicted to regulate BAHD1-associated genes using the Ingenuity's Upstream Regulator Analysis.

Ingenuity Pathway Analysis was used to determine the top Upstream Regulators associated with genes up-regulated in *Bahd1*-KO placentas (E16.5 and E18.5) and MEFs and down-regulated in BAHD1-overexpressing HEK293 cells.

The lists of the top 100 upstream regulators identified in each transcriptome dataset were subsequently crossed highlighting seven transcription factors consistently predicted to regulate a set of genes ("Target Molecules in dataset") in all datasets.

The p-value indicates the significance of the overlap between the genes targeted by the upstream regulator in the IPA database and the experimental dataset.

Upstream Regulator in Mouse Placenta E16.5 transcriptome	Molecule Type	p-value of overlap	03/09/2015
**TP53**	transcription regulator	9,61E-14	A2M,ANTXR1,ANXA8/ANXA8L1,APOE,BNIP3,BOK,CCND2,CDKN1A,CHST12,CLU,ESR1,FABP4,FBLN2,FSTL1,GADD45G,GATM,GLIPR1,GPX3,ID3,IGFBP3,MMP2,NAMPT,P2RX4,PADI2,PCBP4,PHLDA3,PMEPA1,PQLC3,PRNP,PTGS1,RRM1,RRM2,RUNX1,S100A4,SAT1,SCMH1,SGK1,SGPL1,SPHK1,SPP1,SQRDL,TDO2,THY1,TIMP2,TNFRSF1B,TNFSF9,VASN,ZFP36L1
**FOS**	transcription regulator	2,67E-08	A2M,CFLAR,CRABP2,CYP17A1,FABP4,GJA1,HPGD,HSPB8,LY6K,MMP2,PGR,PMEPA1,RUNX1,S100A8,SIRPA,SLPI,SPP1,UGCG,XDH
**ESR2**	ligand-dependent nuclear receptor	1,62E-06	APOE,CDKN1A,CYP11A1,CYP17A1,HPGD,MMP14,MMP2,PGR,SFRP4,SPP1,SUSD4,TIMP2
**SP1**	transcription regulator	1,91E-06	APOE,CCND2,CD55,CDKN1A,CEBPD,CYP17A1,ESR1,GJA1,HPSE,IGFBP3,JAM2,MMP14,MMP2,PGR,SPP1,TFPI2,UGCG
**ESR1**	ligand-dependent nuclear receptor	5,15E-06	APOE,AQP1,CD55,CDKN1A,CPE,CRABP2,CTSS,CYP11A1,CYP17A1,ESR1,KCNK6,LBP,PGR,RAMP3,SGK1,SPP1,TACC1,TNFRSF11B,TXNIP
**EPAS1**	transcription regulator	1,66E-05	ADM,BNIP3,C1QA,EGLN3,FBLN2,GJA1,IGFBP3,LOX,SPHK1
**PPARG**	ligand-dependent nuclear receptor	2,37E-05	APOE,ARL4D,CDKN1A,CRABP2,CXCL14,EGLN3,FABP4,HMGCS2,IGFBP3,IGFBP6,INSIG2,MMP14,S100A8,SPP1,TLR4
**Upstream Regulator in Mouse Placenta E18.5 transcriptome**	**Molecule Type**	**p-value of overlap**	**Target molecules in dataset**
**TP53**	transcription regulator	2,48E-26	A2M,ACSL3,ACTN1,ADD3,ADRB2,AK1,ALDH1A2,ALDH9A1,ANTXR1,ANXA8/ANXA8L1,APOBEC1,AR,AXIN2,BHLHE40,BNIP3,CARHSP1,CASP4,CASP8,CAV2,CCND2,CCNG2,CDC25A,CDC42EP3,CDKN1A,CHMP4C,CHST12,COL5A2,CP,CTSF,DBP,DCK,DKK1,DKK3,DUSP1,EIF4G3,ELF4,ENG,ENPP2,EPHX1,ESR1,FABP4,FBLN2,FGF2,FGFBP1,FHL1,FOS,FOSL1,FSTL1,GADD45G,GAS6,GATM,GDA,GLIPR1,HBEGF,HDAC2,HDAC9,HSPH1,HYAL1,IGF1R,IGFBP3,IRF7,ITGB5,KCNN4,KRT14,MAN2A1,MAP3K8,MCAM,MCM6,MET,MMP2,MPZL2,NAMPT,NDRG2,NEO1,NOX4,NPNT,P2RX4,PADI2,PCDH7,PHLDA3,PIGF,PLA2G16,PLOD2,PML,PQLC3,PRDM1,PRDX6,PTGDS,PTGS1,PTGS2,PTPN1,PVRL3,ROBO1,RRM1,RUNX1,S100A4,SCMH1,SCP2,SEMA3C,SGK1,SGPL1,SLC2A12,SLC6A6,SMC4,SNRK,SPHK1,SPP1,SQRDL,TDO2,TGFA,TGFB1,TGFB2,TGFBI,TGFBR2,THBS1,THY1,TMEM43,TNFAIP2,TNFRSF1B,TNFSF9,TSPO,UMPS,VASN,VIM,ZFP36L1
**ESR1**	ligand-dependent nuclear receptor	8,89E-18	ABCC5,ABLIM1,AHNAK,AQP1,AR,CAV2,CCL2,CCNG2,CD55,CDKN1A,CLIC3,CP,CPE,CRABP2,CTSS,CYP11A1,DDX21,EDN1,ENPP2,ERBB2,ESR1,FGFR1,FOS,FOSL1,FST,GAL,GREM1,ICOSLG/LOC102723996,IFI44,IGF1R,LBP,MALL,NR1D1,OTUB2,PGR,PON3,PRSS23,PTGS2,RAMP3,RARA,RGS19,SAMHD1,SCARB1,SEMA3B,SGK1,SLC2A12,SMC4,SP100,SPP1,SULF1,TACC1,TC2N,TEK,TFF1,TGFA,TM4SF1,TMOD1,TNFAIP2,TNFRSF11B,TXNIP,VAV3,VIM
**FOS**	transcription regulator	2,98E-16	A2M,ADAM12,C3AR1,CCL2,CD244,CERS4,CFLAR,CRABP2,DIO2,FABP4,FIGF,FOS,FOSL1,GJA1,GKN1,HPGD,HSPB8,KCNN4,LARGE,LPL,MET,MMP2,MPZL2,NCF2,NFIL3,PGR,PRDM1,PTGS2,RARA,RARG,RUNX1,S100A9,SEMA3B,SEMA3E,SEMA6D,SFRP2,SIRPA,SLPI,SOCS3,SPP1,SULF2,TGFB1,TIMP1,UGCG,VAV3,VIM,WNT4,WNT5A,XDH
**SP1**	transcription regulator	2,45E-14	ADAMTS1,AEBP1,AR,B4GALT5,CCL2,CCND2,CD55,CDKN1A,CYBRD1,ENG,ESR1,FGF2,FOSL1,GJA1,HPSE,IGF1R,IGFBP1,IGFBP3,JAM2,LPL,MECP2,MET,MMP14,MMP2,MYCN,NCF2,PGR,PLA2G4A,PRKAR2B,PROCR,PTGS2,PTN,PTPN1,RECK,SCARB1,SLC2A12,SLC2A3,SPP1,TFF1,TFPI2,TGFB1,TGFB2,TGFBR2,TIMP1,TSPO,UGCG,VIM,ZEB2
**ESR2**	ligand-dependent nuclear receptor	1,12E-10	AR,ARNT2,CCL2,CDKN1A,CHST15,CYP11A1,DDAH1,ENPP2,FOS,HPGD,IGF1R,MMP14,MMP2,NDRG2,OCLN,PGR,PTGS2,RARA,SCARB1,SCUBE1,SFRP4,SOCS3,SPP1,TCHHL1,TFF1,TGFA,TIMP1,VAV3
**PPARG**	ligand-dependent nuclear receptor	4,18E-10	ABCG1,ACSL5,ADRB2,ARL4D,CAV2,CCL2,CDKN1A,CRABP2,CXCL14,EGLN1,EGLN3,EPHX1,ETS1,FABP4,FABP5,FOSL1,FST,IGFBP1,IGFBP3,INSIG2,LPL,MMP14,NOX4,NR1D1,OLR1,PDK4,PRDM1,PTGS2,RBP4,SAP18,SCARB1,SCNN1G,SCP2,SPP1,SPRR1A,TCF4,TGFBR2,TLR4,VAMP5,VIM
**EPAS1**	transcription regulator	1,38E-07	ADM,ALDOC,ARG1,AXL,BNIP3,C1QA,CYBRD1,EDN1,EGLN3,FBLN2,FHL1,FOS,GJA1,IGFBP3,ITGB3,KDR,SPHK1,TEK,TGFA
**Upstream Regulator in Mouse Embryonic Fibroblasts transcriptome**	**Molecule Type**	**p-value of overlap**	**Target molecules in dataset**
**TP53**	transcription regulator	8,73E-18	ABCG2,ACTN1,ANXA8/ANXA8L1,AREG,BCL2,BCL3,BHLHE40,CAV1,CKB,CLU,CNN1,CRYAB,CSF1,DHCR24,DUSP4,F11R,FHL1,FOSL1,GAS6,HBEGF,HK2,HMGCR,HMGCS1,HSPA1A/HSPA1B,IER3,IL6,INHBA,KRT18,LATS2,LIF,LSS,MCAM,MET,MVD,MYC,NOTCH1,NPNT,NRP1,PDGFA,PDLIM1,PFKFB3,PHLDA1,PIM1,PLAUR,PRC1,PRKAG2,PRKAR2A,PTGS1,PTGS2,SELP,SERPINE1,SLC2A1,SMURF1,SORBS1,TINAGL1,TNFAIP2,UNC5B,VCL
**SP1**	transcription regulator	4,55E-12	B4GALT5,BCL2,BMP4,CAV1,CCL2,CD55,CRYAB,FLT1,FOSL1,HAS2,HGF,HK2,HMGCR,LDLR,MET,MYC,PDGFA,PIM1,PLAU,PLAUR,PRKAR2B,PTGS2,SERPINE1,SLC2A1,SLC7A1,TRIB1
**EPAS1**	transcription regulator	1,05E-10	ADM,AREG,AXL,CAV1,CKB,EDN1,FHL1,FLT1,HMGCS1,ITGB3,LDLR,NOTCH1,PFKFB3,SERPINE1,SLC2A1
**PPARG**	ligand-dependent nuclear receptor	5,07E-10	ABCG2,CAV1,CCL17,CCL2,CTPS1,CXCL14,FOSL1,HGF,HK2,IL6,KRT20,LDLR,MGLL,OLR1,PCTP,PFKFB3,PRKG2,PTGS2,SERPINE1,SLC2A1,SORBS1,SPRR1A,VCAM1
**FOS**	transcription regulator	6,80E-09	ADAM12,ALDH1A3,CADM1,CCL2,CFLAR,EREG,FOSL1,MET,MMP10,MYC,NGF,NSDHL,PHLDA1,PTGS2,S100A3,SEMA3E,SERPINB2,SERPINE1,SMURF1,TSLP,VCAM1
**ESR2**	ligand-dependent nuclear receptor	1,20E-07	BCL2,BMP4,CAV1,CCL2,EREG,HSD17B7,KRT20,MEGF10,MYC,MYCL,PDGFA,PTGIS,PTGS2,TAC1
**ESR1**	ligand-dependent nuclear receptor	1,37E-07	ABCG2,AREG,BCL2,BMP4,CAV1,CCL2,CD55,CKB,EDN1,EREG,FOSL1,GJB2,HMGCR,IER3,IL6,KRT19,LDLR,MYC,PTGS2,RAMP3,SERPINE1,TM4SF1,TNFAIP2
**Upstream Regulator in Human HEK-BAHD1 transcriptome**	**Molecule Type**	**p-value of overlap**	**Target molecules in dataset**
**TP53**	transcription regulator	2,59E-16	ABAT,ABCC1,ACLY,ADA,ADD3,AKR1B1,AKT1,ALDH1A2,ANKH,ANXA6,APOE,ASS1,ATG10,BID,BNIP3,CCND1,CCND2,CCNE1,CDH1,CDH3,CHST12,CKMT1A/CKMT1B,COL2A1,COL4A1,CTSB,CYB5A,CYFIP2,CYP51A1,DBI,DDIT4,DHCR24,DHCR7,DUSP2,EGFR,ENPP2,FASN,FDFT1,FKBP4,FN1,GAS6,GLUL,GPD1L,GSN,H2AFX,H2AFY2,HK2,HMGCS1,HSPB1,IDH2,IFI30,IGFBP2,IL4R,INPP4A,IRF8,ITGA2,ITGB5,KCNG1,KCNMA1,LDHA,LPIN1,LTBP1,MCAM,MDH2,ME1,MGMT,MMP2,MOCOS,MSRB1,MYC,NCK2,NDRG1,NDRG2,NEIL2,NID2,NPTX1,PADI2,PBK,PDIA6,PDK1,PDLIM1,PFKFB3,PFKM,PFKP,PLA2G16,PMEPA1,PPM1F,PRKCB,PRNP,PROM1,PSEN2,PTPRU,SAT1,SCMH1,SERPINB6,SERPINE2,SFN,SGPL1,SGTB,SLC2A1,SLC6A6,SQLE,SREBF1,STARD4,TAP1,TAP2,TGFB1,TIMP2,TMSB10/TMSB4X,TPD52L1,TRAP1,TSPAN6,ULK2,XPNPEP1
**ESR1**	ligand-dependent nuclear receptor	9,22E-07	ABCA3,ADORA1,APOE,ASS1,CA2,CCND1,CCNE1,CDH1,COMT,CRABP2,CTSB,DDIT4,DECR1,DUSP9,EFEMP1,ENPP2,FAM64A,FKBP4,GJB2,HLA-B,HLA-C,IGFBP2,ITGA6,KRT19,LDLR,LGALS3BP,LTBP1,MAP1B,MMD,MYC,NRCAM,OTUB2,PBK,PDCD4,PRKCD,RGS19,SCARB1,SLC13A4,SLC7A2,SLC7A5,TC2N,TMOD1,WNT11,XK
**EPAS1**	transcription regulator	3,73E-06	BNIP3,CKMT1A/CKMT1B,CYP51A1,EGFR,FASN,HLA-DRB3,HMGCS1,LDLR,LOX,MIF,NDRG1,PFKFB3,SLC11A2,SLC29A1,SLC2A1,SLC6A8,SLC7A5,SREBF1
**SP1**	transcription regulator	1,23E-05	ACSL1,ADAMTS1,ANKH,APOE,CCND1,CCND2,CDH1,COL2A1,CR2,CYP51A1,EGFR,FN1,HK2,HMGA1,HSD11B2,ITGA2,KCNQ2,LDLR,LIPA,LPL,MAOB,MMP2,MYC,NDRG1,PDK1,PRKAR2B,PROM1,SCARB1,SLC11A2,SLC2A1,SREBF1,TGFB1,TIMP1,TRIB1
**FOS**	transcription regulator	1,70E-05	ABCC1,ADAM12,ADD2,ALDH1A3,CA2,CCND1,CCNE1,CD44,CERS4,CMTM4,CRABP2,FASN,GM2A,HLA-B,HSPB8,ITGA6,LGALS3BP,LPL,LRP8,LTBP1,MMP2,MYC,NPTX1,NSDHL,PDPN,PMEPA1,RXRA,SCD,SULF2,TGFB1,TIMP1,WNT11
**ESR2**	ligand-dependent nuclear receptor	5,43E-05	APOE,BID,CCND1,CDH1,CES1,CNNM1,ENPP2,GM2A,ITGA2,LTBP1,MMP2,MYC,NDRG2,NID2,RASSF2,SCARB1,SUSD4,THBS4,TIMP1,TIMP2
**PPARG**	ligand-dependent nuclear receptor	2,17E-04	ABCA3,ACLY,ACSL1,ACSL4,AGPAT2,APOE,CA2,CCND1,CDH1,CES1,CPT2,CRABP2,FASN,FN1,HK2,INSIG1,LDLR,LPL,NDRG1,PFKFB3,PLAC1,PLIN2,PTPRF,SCARB1,SCD,SDC1,SLC2A1,SREBF1,UCP2,VAMP8
